# Setting priorities for ageing research in Africa: A systematic mapping review of 512 studies from sub-Saharan Africa

**DOI:** 10.7189/11.15002

**Published:** 2021-06-26

**Authors:** Michael E Kalu, Blessing U Ojembe, Olayinka Akinrolie, Augustine C Okoh, Israel I Adandom, Henrietta C Nwankwo, Michael S Ajulo, Chidinma A Omeje, Chukwuebuka O Okeke, Ekezie M Uduonu, Juliet C Ezulike, Ebuka M Anieto, Diameta Emofe, Ernest C Nwachukwu, Michael C Ibekaku, Perpetual C Obi, Emerging Researchers and Professionals in Ageing-African Network

**Affiliations:** 1 Emerging Researchers & Professionals in Ageing – African Network, Nigeria (www.erpaan.org); 2 School of Rehabilitation Science, McMaster University, Hamilton Ontario Canada; 3 Department of Health, Ageing & Society, McMaster University, Hamilton Ontario Canada; 4 Applied Health Science Program, University of Manitoba, Winnipeg, Canada; 5 Physiotherapy Department, Cedacrest Hospitals, Abuja, Nigeria; 6 Faculty of Health Science, University of Lethbridge, Lethbridge, Alberta, Canada; 7 Department of Medical Rehabilitation, College of Medicine, University of Nigeria, Nigeria; 8 Department of Medical Rehabilitation, Nnamdi Azikiwe University, Awka, Nigeria; 9 Department of Social Work, University of Nigeria, Nsukka, Nigeria; 10 Department of Social and Behavioural Sciences, City University of Hong Kong, Kowloon, Hong Kong; 11 Department of Health and Rehabilitation Sciences, University of Cape Town, Cape Town, South Africa; 12 Physiotherapy Department, Humanity Hospital Effurun, Delta State, Nigeria; 13 Physiotherapy Department, Enugu State University of Science and Technology Teaching Hospital-Parklane, Enugu, Nigeria; 14 Department of Physiotherapy, University of Benin Teaching Hospital, Benin city, Nigeria; 15 Physiotherapy Unit, Peak Wellness Centre, Abuja, Nigeria

## Abstract

**Background:**

In 2040, the older population's growth rate in sub-Saharan Africa (SSA) will be faster than those experienced by developed nations since 1950. In preparation for this growth, the National Institute on Aging commissioned the National Academies' Committee on Population to organize a workshop on advancing aging research in Africa. This meeting provided a platform for discussing some areas requiring improvement in aging research in SSA regions. We believed that conducting a systematic review of peer-reviewed articles to set priorities for aging research in SSA is warranted. Therefore, this article is the first in a Four-Part series that summaries the types and trends of peer-reviewed studies in SSA.

**Methods:**

This systematic mapping review followed the *Search-Appraisal-Synthesis-Analysis* Framework. We systematically searched multiple databases from inception till February 2021 and included peer-reviewed articles conducted with/for older adults residing in SSA. Conventional content analysis was employed to categorize studies into subject-related areas.

**Results:**

We included 512 studies (quantitative = 426, qualitative = 71 and mixed-method = 15). Studies were conducted in 32 countries. Quantitative studies included were observational studies: cross-sectional (n = 250, 59%), longitudinal (n = 126, 30%), and case-control (n = 12, 3%); and experimental studies: pre-post design (n = 4, 1%), randomized control trial (RCT, n = 12, 3%); and not reported (n = 21, 5%). Fifteen qualitative studies did not state their study design; where stated, study design ranged from descriptive (n = 14, 20%), ethnography (n = 12, 17%), grounded theory (n = 7, 10%), narrative (n = 5, 7%), phenomenology (n = 10, 14%), interpretative exploratory (n = 4, 6%), case studies (n = 4, 6%). Of the 15 mixed-method studies, seven did not state their mixed-method design. Where stated, design includes concurrent (n = 1), convergent (n = 1), cross-sectional (n = 3), informative (n = 1), sequential exploratory (n = 1) and retrospective (n = 2). Studies were classified into 30 (for quantitative studies) and seven (for qualitative and mixed-method) subject-related areas. HIV/AIDs-related and non-communicable diseases-related studies were the most predominant subject-related areas. No studies explored the transdisciplinary co-production of interventions.

**Conclusions:**

There are glaring gaps in ageing research in SSA, especially mixed-methods and RCTs. A large number of studies focused on HIV/AIDs and non-communicable disease-related studies. National and international funding agencies should set up priority funding competitions for transdisciplinary collaborations in ageing research.

With increasing life expectancy, older people’s population is projected to grow worldwide, including the world's developing regions [1]. In 1990, there were 23 million older people aged 60 years or over in sub-Saharan Africa (SSA), and it has increased to 46 million over the space of 15 years and will increase to 161 million by 2050 [1]. Presently, the growth rate of older adults in developed regions of the world is faster than that experienced in the sub-Saharan region. However, in 2040, older adults' growth rate in SSA will be faster than those experienced by developed nations since 1950 [2]. Generally, older people aged 60 years or over comprised five percent of the overall population in the SSA in 2015; although, there are some countries where the proportions of older persons were much higher. More than 15 and 11 percent of Mauritius and Seychelles' populations were aged 60 years or over, respectively [1].

Although the population of the older adults in the SSA seems not to be the primary concern of several member nations in this region, evidence has demonstrated the impact of these growing populations on the country's economy, health care system, educational system and even workforce [3]. The labour force participation of older persons in Africa is the highest globally [4]. In Africa, 52% of men and 33% of women, aged 65 years and older, were active in the labour force in 2015 compared to only 10% of men and 6% percent of women in Europe and 38% of men and 17% of women aged 65 years in Caribbean region [5]. In most SSA countries, a higher percentage of caregivers are older people; this keeps them active in the labour force. For instance, in Zimbabwe and Namibia, an estimated 60% of orphaned children were cared for by their grandmother [6]. Additionally, the SSA's health care systems are confronted with a growing burden of illness due to non-communicable diseases (NCDs) associated with old age [6]. Hence the need to set priorities for ageing research and care in the SSA regions.

The United Nations has advocated for more evidence/data to understand the status and the needs of older persons in the SSA. For instance, in 2004, the National Institute on Aging commissioned the National Academies’ Committee on Population to organize a workshop on advancing ageing research in Africa [7]. This eight-member panel commissioned 12 papers that focus on the changing demographics in SSA, the demographic impact of the HIV epidemic on older people, formal and informal social security systems, health, measurement, the impact of social pensions, the situation of older people in urban areas, living arrangements, and policy. While this meeting provided a platform for discussing some areas that require improvement in ageing research, they only selected some papers. Hence the need to conduct a comprehensive systematic review aimed at mapping research conducted in SSA into subject areas; this will help understand the areas that needed further research.

The World Health Surveys and the follow-up on Global Aging (SAGE) through its Wave I & II has improved data availability on health and related social issues of ageing in three SSA countries: Ghana, South-Africa, and Uganda [8]. There are also national or regional longitudinal studies in ageing, for instance, Ibadan Longitudinal study in Ageing. Alongside individual studies conducted in SSA countries, we believed that understanding the types and trends of evidence of Ageing Research in the SSA is warranted. We aim to scope the literature, provide summaries of research types in SSA, and provide recommendations on areas that need more research. This study revisits previous ageing research priorities set by the eight-member panel in 2014 by conducting a systematic mapping review of published peer-reviewed articles in the SSA. This review is divided into four series:

***Part I*** summarises the types and trends of ageing research in SSA and provided specific recommendations regarding the types of ageing research in SSA.
***Part II*** will report the quality of the included studies (quantitative, qualitative and mixed-method).
***Part III*** will review the longitudinal studies of Ageing in Africa and describe the impact of longitudinal studies in Ageing on the ageing research in SSA.
***Part IV*** will present a set of ageing research priorities in SSA developed by stakeholders (older adults, family caregivers, professionals-academicians and non-academicians) through a modified Delphi process.

This paper: Part 1 summarises (a) the types and trends of ageing research in SSA and (b) provides updated specific recommendations for ageing research in SSA.

## METHODS

This study was a systematic mapping review – a subtype of a review that characterized quantity and quality of literature using study design, sampling techniques, data collection and data analysis [9]. This review followed the *Search-Appraisal-Synthesis-Analysis Framework* (SALSA) [9], which provides a structured framework for searching the literature, selecting articles to be included or excluded based on pre-determined eligibility criteria synthesizing and analyzing the findings from each included article.

### Search strategy

We searched the literature in consultation with an expert librarian in medical and social literature. We searched the following databases: PubMed, EMBASE, CINAHL, PEDRO (physical evidence database), Cochrane CENTRAL, PsychINFO and Web of Science, from inception till February 2021. We adopted a combination of medical subheadings (MeSH) and keywords – “aged” OR “older adult*” OR “older people” OR “elderly” OR “senior citizen*” OR “ag*” OR “gerontology” OR “geriatrics” AND “Africa*” OR “Africa South of Sahara” OR “Africa, Western” OR “Africa, Southern” OR “African, Eastern” OR “Africa, Central” OR “South Africa OR Nigeria OR Ghana OR Uganda OR Zambia OR Cameroon OR Zimbabwe OR Rwanda OR Kenya OR Gambia,” for each database listed above (see Appendix S1 in the **Online Supplementary Document** for example of the search strategy for CINAHL).

### Study selection

We included an article if:

- the population under study was older adults (55 years old), the age bracket referred to as being older adults in the SSA region [10], or at least 70% of the study population is 55 years and above or median/mean age of the population is 55 years. SSA countries are African countries fully or partially located to the south of Sahara; 46 of Africa's 54 countries excluding Algeria, Djibouti, Egypt, Libya, Morocco, Somalia, Sudan and Tunisia [11].
- the study reported data either as a quantitative variable or qualitative themes or both.
- the phenomena *of interest focused* on older adults (directly or indirectly) in the SSA region. Direct focus studies focused research on older adults or conducted research with or for older adults. Indirect focus on older adults are studies that, for instance, explored the perception or knowledge of healthcare workers on their interest in working with older adults or care experiences of healthcare workers, healthcare students, the older adults' relatives and the general population as it regards to older adults.
- it was published in the English Language from inception to February 2021. This time frame allowed us to understand the trends of ageing research in SSA.

We exclude perspective papers (ie, commentaries/opinion papers without data). We looked at the individual studies for systematic review articles and included only those that met our inclusion criteria.

Duplicates of studies from database search were removed in RefWorks [12]. Studies were selected in two stages: title and abstract screening and full-text screening. Six reviewers independently conducted a pilot trial of title and abstract screening of the first 300 articles to determine inter-rater reliability. Light's kappa (average kappa across all raters’ pairs) of the raters was 0.89 for the title and abstract screening and 0.90 for full-text screening indicating an excellent agreement between raters [13]. Due to the high level of agreement, the retrieved articles were divided, and each reviewer independently conducted the title, abstract and full-text screening. We had research team meetings to clarify any disagreement.

### Extracting the evidence

The first author (MK) developed the data extraction template and pilot-tested it in previous reviews [14]. Four reviewer authors conducted a pilot testing of data extraction using the template and subsequently extracted them independently. Any disagreement was resolved in a team meeting with at least six members in attendance. We extracted the following information from each article: the type of study (quantitative or mixed), authors' names, year, the title of the article, country, state/province the study was conducted in, the aim of the study/research question, specific disciplines, research settings, outcome measures, conditions studied, study design, sampling method, time followed, recruitment strategy, statement of ethical approval, statement of informed consent, sample size, participant characteristics (eg, sample size, sex, age), data analysis method, significant findings, conclusion, future research/policy/clinical recommendation, and study limitations.

### Data synthesis

We described the trends and other characteristics, including research areas, sampling methods, sample size, research settings by types (eg, quantitative, qualitative and mixed-method) of the included studies. We described the subject area using a two-step approach. First, we mapped the ageing research in SSA to predetermined faculties where the principal or corresponding author has affiliations. These faculties include health science, biological sciences, social science, physical sciences, environmental sciences, engineering, art/humanities, and business/commerce. In cases where the principal/corresponding authors are independent authors with no faculty affiliation, we contact them inquiring about their affiliation. Second, we created subject areas using conventional content analysis [15]. Four author reviewers (in pairs) read line-by-line the included studies' aim and title while reflecting on the study population characteristics, including age, phenomena of study, to derive codes. Each author (in pairs) notes their impressions on the meaning of the codes for each included study. Each pair met and derived sub-themes and themes for each included study.

Each paired author reviewer presented their themes and sub-themes in a research team meeting (consisting of 3 additional authors). We chose the “best fit” theme and sub-theme for each included article. Sub-themes may include studies on medical/social conditions (eg, hypertension or dementia), health outcomes (eg, mortality or functional disability) or phenomena of study (eg, experience of health care services, elder abuse, normative health values). Sub-themes were merged to determine themes, referred to ***as subject-related areas.*** For instance, conditions such as hypertension, diabetes were classified as non-communicable diseases-related studies, and stroke and Parkinson disease were classified as related neurological studies, normative values were classified as physiological values. Content analysis was performed for quantitative, qualitative and mixed-method studies separately. We presented the themes and sub-themes in frequency counts.

## RESULTS

**Figure 1:**
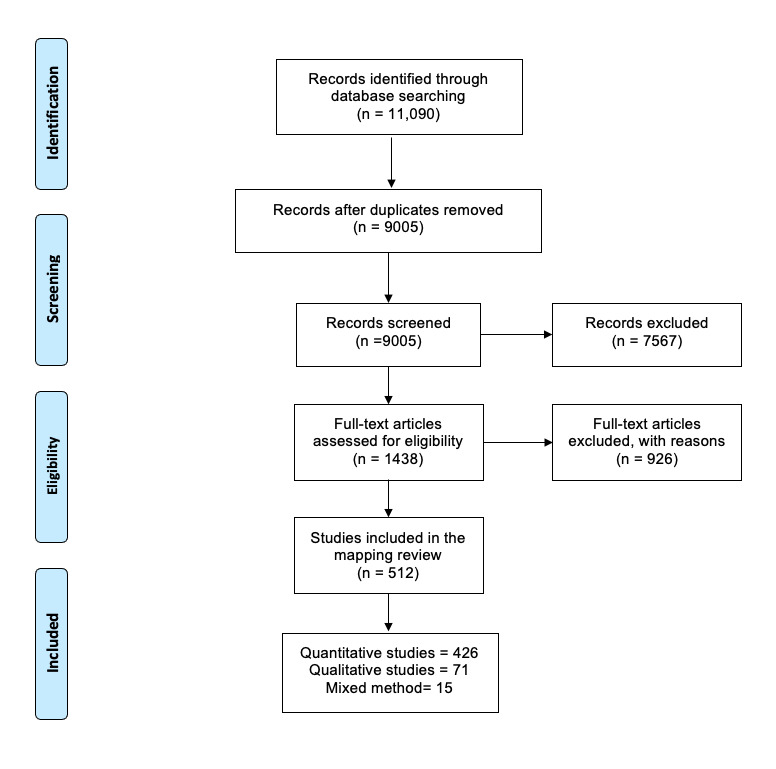
PRISMA flowchart of the included articles.

We retrieved 11 090 citations from the database searches. After removing duplicates, 9005 citations underwent abstract and title screening, and we excluded 7567. The remaining 1438 articles underwent full-text screening, and we included 512 (**Figure 1**). The included studies are presented in Appendix S2 in the **Online Supplementary Document**.

**Figure 2:**
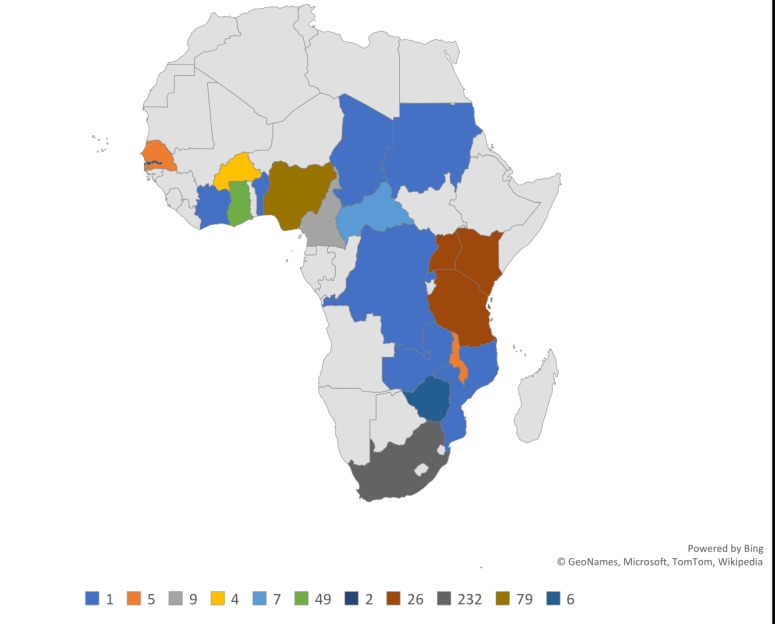
The SSA countries ageing-related research was conducted (n = 492/512). Twenty articles were conducted in more than one country.

The articles included in this review were conducted using quantitative method (n = 426, 83%), qualitative method (n = 71, 14%) and mixed-method (n = 15, 3%). Forty-five percent of the included studies were conducted in South-Africa. Other countries in which included studies were conducted included Angola, Benin, Botswana, Burkina Faso, Cameroon, Central African Republic, Chad, Congo (Brazzaville), Democratic Republic of Congo, Cote d'Ivoire, Ethiopia, Gabon, Gambia, Ghana, Guinea, Kenya, Malawi, Mauritania, Mozambique, Namibia, Niger, Nigeria, Senegal, Swaziland, Tanzania, Togo, Rwanda, Uganda, Zambia, Zimbabwe. Twenty articles were conducted in more than one country (**Figure 2**).

**Figure 3:**
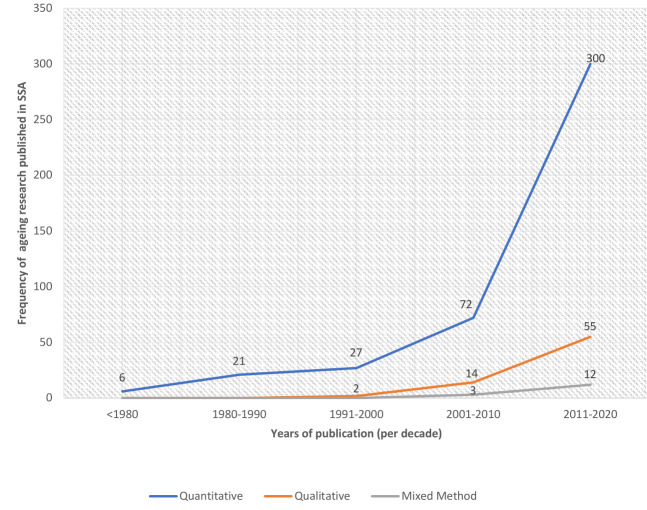
The trend of ageing research in Sub-Saharan Africa.

Seventy-two percent of the articles were published from 2010 to 2020 (see **Figure 3** for trend).

### Quantitative studies

Of the 426 included quantitative articles, 369 (87%) principal/corresponding authors' affiliation were health sciences, while 56 (13%) were social science, and one was humanities. The sample size ranged from 7 [16] to 392 131 [17]. While 104 studies (24%) had a sample size of fewer than 200 participants, 127 studies (30%) and 197 studies (46%), respectively, had a sample between 201 and 1000 and more than 1000 participants. Five studies did not report their sample size[18-22].

Participants were recruited from the community (n = 249,58%), hospitals (n = 137, 32%), or both hospital and community (n = 18, 5%), and nursing homes (n = 7, 2%), but not reported in 15 studies (4%). Whereas 166 (39%) studies were conducted in urban areas, 115 (30%) in rural areas, and 120 (28%) studies were conducted in both urban and rural settings. Ninety-nine (23%) articles did not state their research settings.

Quantitative studies included were mostly observational studies [cross-sectional (n = 250, 59%), longitudinal (n = 126, 30%), and case-control (n = 12, 3%)], and experimental studies [pre-post design (n = 4) [23-26]; randomized control trials (RCT, n = 12, 3%) [27-38]. Five percent of the included articles (n = 21) did not state their study designs. Ninety-eight (23%) articles did not state their sampling method. Where stated, probabilistic sampling ranged from simple random (n = 75, 18%), multi-stage sampling (n = 57, 13%), stratified sampling (n = 34, 8%), cluster sampling (n = 14, 4%) and systematic random sampling (n = 9, 2%). The non-probabilistic sampling method was convenience sampling (n = 79, 19%), purposive sampling (n = 32, 8%), consecutive sampling (n = 24, 6%), and quota sampling (n = 4, 1%).

Three-quarter of the included articles (n = 313, 73%) provided statements of obtaining ethical approval for their study or that ethical approval is not required, while 113 (27%) articles did not provide this statement. More than half of the included articles (n = 262, 62%) provided statements of obtaining informed consent from the participants before commencing with data collection.

#### Subject-related areas for quantitative studies

Our content-coding resulted in 30 subject-related areas. Most studies included were categorised into Non-communicable disease-related studies (n = 41), HIV-related studies (n = 30), Physical functioning-related studies (n = 26), Cancer-related studies (n = 28), quality of life/well-being related studies (n = 22), Dementia/cognitive impairment-related studies (n = 30), Neurological-related studies (n = 15). See **Table 1** for the description of all the subjected areas for the included quantitative studies.

**Table 1:** Subject-related areas for the quantitative studies

Subject areas for quantitative studies (n = 426)	Frequency
**Non-communicable disease-related studies(NCDs)** are studies that explore (a) risk factors, prevalence, incidence, screening, treatment of NCDs and mortality/survival rate of older adults living with NCDs, and (b) the association between NCDs (including diabetes, hypertension) and health outcomes (eg, physical functioning)	41
**HIV/AIDs-related studies** include studies that risk factors, prevalence, incidence, screening, treatment of HIV/AIDS, and mortality/survival rate of HIV/AIDS among older adult	30
**Cancer-related studies** explored risk factors, prevalence, incidence, screening, treatment, and mortality/survival rate of cancer among older adult	28
**Physical functioning-related studies** include studies that explored risk factors, prevalence, incidence, screening, treatment, and mortality/survival rate associated with physical functioning related outcomes (eg, falls, frailty, fracture) among older adults	26
**Quality of life/well-being-related studies** explored the well-being and quality of life of older adults and sometimes their caregivers and its association to other health, social and psychological outcomes.	22
**Dementia-related studies** explored the risk factors, prevalence, incidence, screening, treatment of dementia, and mortality/survival rate of older adults living with dementia.	20
**Healthcare utilization-related studies** explored how older adults use health care services and the accessibility of health care services.	20
**Eye related studies** explored the risk factors, prevalence, incidence, screening, treatment of eye conditions among older adults	17
**Neurological-related studies** included studies on stroke, its prevalence, incidence, risk factors, treatment outcomes associated with strokes, and the mortality rate of older adults with a history of stroke or Parkinson diseases.	15
**Generic health-related studies** explored the association between health and some demographics (eg, marital status, race); health behavior, health needs, perceived health, use of health product	16
**Psychological-related studies** explore different kinds of mental health issues, including delirium, depression, prevalence, risk factors, and relationships with other health and social conditions.	16
**Cardiovascular-related studies** explored risk factors, prevalence, incidence, screening, treatment, and mortality/survival rate of heart diseases among older adult	13
**Nutrition-related studies** explored the association between nutrition and health outcomes (eg, quality of life) or dietary requirement for older adults	13
**Care provision-related studies** described intergenerational care, experiences of older adults, health care professionals, informal caregivers as it regards providing care to older adults	13
**Mortality/survival-related** studies described the mortality/survival rate of older adults in a data set or a geriatric clinic with no link to any NCDs or other health and social conditions	13
**Physiologic-related studies** include studies that explored physiological normative values, physiological response (eg, dizziness or drug effect), and its prevalence among older adults	12
**Cognitive impairment-related studies** explored the association between cognitive impairment (not dementia) and other health outcomes among older adults.	10
**Other disease-related studies** (eg, bone and abdominal disease) explored the characteristics, risk factors, and treatment options of conditions that are not listed as NCDs, Cancer, or HIV	10
**Sleep-related studies** explored the risk factors, health risks, quality and quantity of sleep, prevalence, and mortality rate associated with other health outcomes among older adults	9
**Instrument validation studies** explored the content or validation of outcome measures such as depression or cognition scales among older adults and health care workers	9
**Dental-related studies** explored the risk factors, prevalence, incidence, screening, treatment of dental issues among older adults	8
**Demographic data studies** described the percentage of older adults characteristics either in hospital records or in a national census or groups of older adults with successful aging	7
**Housing/household studies** explored living arrangements and social positions as it regards older adults	7
**Behavioral studies** are studies related to the behavior of older adults (for instance, alcohol use or tobacco, or suicidal behavior)	6
**Physical (PA) activity related studies** explored the risk factors, prevalence, or incidence of PA among healthy older adults or older adults living with health conditions, eg, stroke)	6
**Disability studies** explored the experience of older adults living with disability not caused by HIV or any of the NCDs mentioned above, including dementia and depression	5
**Respiratory conditions studies** explored the risk factors, prevalence, incidence, screening, treatment of respiratory conditions among older adults	4
**Multimorbidity studies** explored the prevalence, risk factors of developing multiple health conditions among older adults	3
**Pension related studies** explored the impact of pension on the health and well-being of older adults	3
**Other studies include** facial aging pattern study (n = 1), poverty study (n = 2), sexuality study (n = 1), social engagement, capital, support and participation (n = 4), prosthesis (n = 1), Spirituality = 1, grief and death (n = 1), sarcopenia (n = 3), pension (n = 3), Tuberculosis studies (n = 2), Migration (n = 2), prescription (n = 3)	24

### Qualitative studies

Of the 71 included qualitative articles, 38 (54%) and 33 (46%) principal/corresponding authors' affiliations were health sciences and were social science, respectively, and one was humanities. The sample size ranged from 1 [39,40] to 214 [41]. Included studies recruited participants from the community (n = 52, 74%), the hospital (n = 16, 23%) and nursing home (n = 2, 3%). Whereas 25 (35%) studies were conducted in rural areas, 22 (31%) were conducted in urban areas. Also, 22 (31%) were conducted in both urban and rural settings, while two studies (3%) did not state the research setting [42,43]. Fifteen (21%) articles did not state their study design. Where stated, study design ranged from descriptive (n = 14, 20%), ethnography (n = 12, 17%), grounded theory (n = 7, 10%), narrative (n = 5, 7%), phenomenology (n = 10, 14%), interpretative exploratory (n = 4, 6%), case studies (n = 4, 6%). Fifty-seven (80%) studies purposely sampled their participants, while 14 (20%) used a convenience sampling method. Of those that used a purposive sampling method, 17 (30%) were criterion-based, three (5%) were random purposive, three (5%) were maximum variation, two (4%) used snowballing techniques, and 30 (59%) did not state the specific type of purposive sampling used. Forty-three articles (61%) collected data using individuals interviews (n = 31) focus group discussion (n = 8), observation (n = 1) or document review (n = 3), while 27 (38%) collected data using a combination of two or more data collection methods: individuals interviews and focus groups discussion (n = 19), individual interviews and observation (n = 2), individual interviews, focus groups and observation (n = 4), individual interviews, observation and document review (n = 2). Statements of ethical approval were noted in 64 articles and not in three articles [39,44,45]. Fifty-eight articles stated that informed consent was obtained from participants, whereas one article did not. Three articles reviewed documents [46-48]; hence they do not need consent from participants. Twenty-four articles [44,45,49-71] discussed their studies' rigour process, while the remaining 47 articles did not.

### Mixed-method

Of the 15 mixed-method studies, the principal/corresponding author's affiliation was in health sciences (n = 13, 85%) and social sciences (n = 2, 15%). Participants were recruited from the hospital (n = 7, 47%), and community (n = 8, 53%); in rural (n = 8, 53%), urban (n = 4, 27%), both (n = 2, 13%) settings and not described in one study [72]. Six studies did not describe the type of mixed-method [72-77]. Where described, the type of mixed-methods includes concurrent [78], convergent [79,80], cross-sectional [81,82], informative [83], sequential exploratory [84] and retrospective [85,86]. The sampling method included convenience [74,80,82,86], purposive [76,83,85], random [75,77,78], all-inclusive sampling [79]. The sampling method was not stated in four studies [72,73,81,84]. Statements of ethical approval were provided in all 15 included studies. All studies provided statements of informed consent except one [72]. Sample sizes ranged from 8 [78] to 15228 [75].

The themes from the qualitative and mixed-method studies were group into seven subject related areas. They include health/social care experience (n = 24), HIV/AIDs related studies (n = 21), contemporary studies (n = 14), older peoples’ care-related studies (n = 14), loneliness-related studies (n = 5), dementia care-related studies (n = 4) and quality of life/physical functioning related studies (n = 4). See **Table 2** for the description of the subject-related areas.

**Table 2:** Subject-related areas for the qualitative and mixed methods studies

Subject areas for qualitative (n = 71) and mixed-method studies (n = 15)	Frequency
**Health/social care services/experiences-related studies** explored the experiences of HCPS, informal caregivers, older adults on receiving or giving care to older adults. For instance, experiences of HCPs in falls prevention practice	24*
**HIV-related studies** explored the experiences of older adults in providing care to children living with HIV or the challenges of aging with HIV/AIDs or factors influencing accessing ART for HIV/AIDs	21*
**Contemporary studies** explored recent discussion on aging, including aging across the life course, elder abuse, gender and aging, neoliberalism and apartheid effects on the aging population, death in the family, poverty, and pension	14*
**Older peoples' care-related studies** explored several areas of care provision directly related to older adults, including intergenerational care, vulnerability/challenges in providing care and receiving care, family support, social responsibilities, and survival strategies among older adults amid poor economic situation in some SSA countries	14*
**Loneliness-related studies** explored experiences of loneliness, social isolation, and the impact of loneliness among loneliness	5
**Dementia care-related studies** explored the experiences and cultural beliefs associated with dementia care	4*
**Quality of life/physical performance-related studies** explored the quality of life and physical performance ranging from conceptualization of quality of life from the perspective of older adults and their activities of daily living	4

*The score of which these HIV-related studies (n = 4), older peoples' care (n = 3), health/social services related studies (n = 5), contemporary studies (n = 2), and dementia-related study (n = 1) were conducted in a mixed-method paradigm.

## DISCUSSION

This mapping review summarized the types and trends of research on ageing in the SSA region. Included studies were predominantly quantitative-cross-sectional studies, published in the last ten years and mainly in South Africa, with HIV-related studies and non-communicable disease-related studies being the most predominant studies on ageing in SSA. These studies published mainly in the social and health science paradigm increased over the last ten years could indicate interest in researching to improve older adults' health and social life in SSA. We could not find any empirical evidence to support or refute if ageing research has increased in the Latin American and Caribbean countries with a similar projection of the ageing population with the SSA countries.

Almost half of the included studies were conducted for or with older adults residing in South-Africa. This finding highlights the underrepresentation of other SSA countries, indicating a dearth of ageing research in SSA countries outside South Africa. This finding agrees with the previous review exploring end-of-life research in SSA, reporting a lack of socio-cultural aspects of end-of-life care in African countries outside South-Africa [87]. This high ageing research outcome from South Africa could be because of several reasons. Excluding Mauritius and Seychelles, the proportion of older adults in South Africa is higher than in other SSA countries [88]. The availability of resources, including research funding and research expertise, facilitating the publication of research in peer-reviewed journals, could be another reason. Besides, South-African researchers may have more collaborations with countries outside SSA that have extensive and established research programs on the ageing population, as evidence by the WHO-SAGE study [8]. Nevertheless, efforts to increase ageing research in countries with the highest proportion of older adults such as Mauritius and Seychelles, whose older adults (60 years and older) account for about more than 15% and 11% of their populations, respectively, are needed.

Aside from systematic reviews and meta-analysis, randomized control trials (RCT) are the highest level of evidence for clinical decision-making [89]. Two percent of the studies included in this review were RCT. We could argue that most ageing research in SSA regions does not focus on evaluating the effectiveness/efficacy of interventions to improve older adults' health and social outcomes, including quality of life and wellness. Notably, 30% of the articles included in this review reported findings from longitudinal studies in ageing. This percentage is considerable since the older adults' population (60+) is the lowest among any other populations, including children and younger adults in most SSA countries. Nevertheless, most longitudinal studies focused on understanding the latter part of life opportunities regarding risk factors that predict poor health outcomes among older adults (eg, mortality and morbidity). Due to the projected increase in non-communicable diseases among young adults and older adults in the SSA, most longitudinal studies focus on identifying risk factors that predispose older adults to develop non-communication diseases such as stroke and heart attacks. For instance, The Health and Aging in Africa: A Longitudinal Study of an INDEPTH community (HAALSI) in South Africa aimed to understand the major forces shaping the trajectory of the prevalence, incidence and risk factors of cardiometabolic diseases in a cohort of 5509 individuals [90]. WHO-SAGE (Wave I & II) recruited participants from Ghana, Uganda and South Africa, amongst another low/middle-income countries, to examine patterns and dynamics of age-related changes in health and well-being using longitudinal follow-up of a cohort as they age and investigate socioeconomic consequences of these health changes [8]. We believe it is high time that longitudinal studies examine life-span development (ageing from childhood to adolescence into adulthood and older age) in the SSA regions. They could include genomic and genetic parameters alongside the socio-demographic, physical, cognitive functioning variables.

There are fewer qualitative studies compared to quantitative studies included in this review. This is not surprising because historically, quantitative studies are often valued than qualitative studies [91], maybe because most quantitative studies provide a convincing argument for generalization of research findings. Nevertheless, qualitative studies are vital in understanding the experiences of older adults, family caregivers, and formal caregivers regarding interventions, policies and geriatric care implementations. Besides, qualitative evidence provides lived experiences of older adults and their family caregivers that can inform the family-patient-centred approach to care, which is the recommended approach for older adult's care [92]. Therefore, qualitative research should be viewed as complementary to high-quality RCTs instead of lesser quality. Most qualitative studies included in our review had flawed methodologies, including lack of report of study designs, sampling techniques (eg, criterion-based or maximum variation purposive sampling) and strategies to ensure rigour. We understand journals' word count limit may have hindered the reporting of some of these methods, especially the strategies to ensure rigour. Based on our experience, we suggest that qualitative researcher concisely provide a statement of rigour when stating the study strengths or data analysis section, eg, we used double coders to triangulate the study themes. Descriptive qualitative studies constitute the higher percentage compared to other qualitative study design (eg, interpretative phenomenology or grounded theory) that contains some form of interpretation of data aimed at providing conceptual themes emerging into a conceptual framework that could inform health and social care practice [93]. With fewer high-ranked qualitative studies, regional ageing policy decision-making based on lived experiences could be difficult with the current level of qualitative ageing research in SSA.

The mixed-method was the least studied method used in ageing research in SSA. Although 64% of researchers reported the type of mixed-method design (eg, convergence and concurrent), they failed to demonstrate when the qualitative and quantitative data were mixed; maybe because of the many journals' word counts limit. Mixed-methodology researchers had advocated that researchers who intend to use a mixed-method as their research method must demonstrate when and how the data was mixed [94]. The mixed-methods qualitative and quantitative components must be conceptualized and methodologically developed individually before mixing their data to answer the overarching study research question(s) [91]. However, most mixed-method studies included in this review did not fully develop their qualitative components; for instance, some researchers considered an open-ended comment section of a quantitative survey as a qualitative component of a mixed-method study. Such research may not fully qualify as a mixed-method study [91].

HIV-related studies and non-communicable disease (NCDs)-related studies were predominant. With the advent of antiretroviral drugs, individuals residing in SSA countries are treated in the early stages of the diseases, which has increased their life expectancy to at least 50 years [95]. Most of these individuals live to become grandparents, and they often provide care for their grandchildren [6]. With chronological age to be considered an older adult in most SSA countries is 55+ [10], our review captured many HIV-related studies. Additionally, the number of research on NCDs-related studies was not surprising because most NCDs-hypertension, diabetes, and stroke cause considerable and growing morbidity and mortality burden among older adults in the SSA [96].

Unfortunately, the SSA's ageing research focuses more on health and social sciences, with little to no contribution from humanities, environmental or physical sciences. In most developed countries, environmental scientists (eg, architecture and regional planners) have collaborated with the health authorities to develop accessible neighbourhoods (eg, age-friendly cities) or housing systems for older adults [97]. This collaboration highlights the role of transdisciplinary collaboration, which encourages professionals outside the typical health and social care system to participate in providing care for older adults.

This study is the first to provide a systematic mapping review that summarises ageing research in SSA; however, it has some limitations. Because this review is broad, the findings are reported in a Four-Part series; this paper is Part I. The quality assessment and detailed description of specific research areas such as HIV related research and care provision research will be published in subsequent papers. The study's limitations include missed studies due to the number of countries included in the search strategies. In addition, we only included peer-reviewed literature published in English, and some of the national journals in SSA are not indexed in MEDLINE or related databases [98].

## RECOMMENDATIONS

1. We recommend more RCTs that focus on examining the effectiveness of interventions to improve health outcomes among older adults in SSA. National and international funding agencies should set up specific funding competitions to encourage ageing research to develop high-quality RCTs.
2. The SAGE and other longitudinal studies in SSA countries have provided valuable information about risk factors that influence age-related health and wellbeing changes. We recommend that national and international funding agencies develop or commission a longitudinal study to examine life-span development (ageing from childhood to adolescence into adulthood and older age) in the SSA regions. The longitudinal studies could also involve genetic and genomic biomarkers of ageing rather than the commonly used epidemiological variables, including socio-demographic-economic determinants of health.
3. The growth HIV/AIDs epidemic has levelled off in the SSA since the late 1990s [99]. We recommend that ageing research focus more on improving care or maintaining health outcomes among older adults and their relatives. Efforts to increase healthcare professionals' interest in working with older adults are recommended, as it is the most natural approach to building capacity in preparation for ageing research. Such efforts could include developing scholarships to encourage healthcare workers and students to study or conduct research in ageing. For instance, the Commonwealth Scholarships on Age-related studies (eg, gerontology degrees or certificates) and Community-Based Initiatives in Ageing Scholarships developed by the Emerging Researchers and Professionals Ageing-African Network (https://erpaan.org/funding.html) are examples of strategies to encourage ageing research in SSA.
4. One reason for the underrepresentation of ageing research from other SSA countries could be a lack of funding to conduct ageing research or lack of funding to publish in an open-peer review. Most open-access journals offer article processing fees (APF) waiver to only researchers residing in lower-income countries. The majority of ageing research was conducted by researchers residing in upper and lower-income countries, eg, Nigeria, South Africa and Kenya. Although open access journals offer at least a 50% APF discount for researchers in middle-income countries, most researchers in this region struggle to pay. We recommend that national and international funding agencies subsidize the APC fees for authors whose articles have undergone peer review and are considered to impact either older peoples' policy or care provision substantially. Committees should be set up to develop strategies, criteria and frameworks to determine ageing articles or studies with a potential of substantial impact in SSA.
5. Transdisciplinary co-production approaches encourage stakeholders' active involvement, including older adults, their relatives and professionals (academic and non-academic) who are not typically part of older adult's care. Such professionals could include engineers who design mobility aids for older adults or adaptive devices for driving assistance among older adults. We did not see such collaborations in ageing research in SSA from the included studies.

In conclusion, this review's articles indicated that ageing research in SSA is a predominantly cross-sectional quantitative study design. Noteworthy is the small number of RCTs found in this review. Among the few qualitative studies, descriptive studies represent a higher percentage, while mixed-method studies were the least methodology employed by ageing researchers in SSA. The next series of this review will describe the quality of articles included in this systematic review.

## Additional material

Online Supplementary Document
