# Supplementary material for: Setting priorities for ageing research in Africa: A systematic mapping review of 512 studies from sub-Saharan Africa
Source: J Glob Health. 2021 Jun 26;11:15002. doi: 10.7189/11.15002 (PMC8284542; doi:10.7189/11.15002)
Supplement: Online Supplementary Document [file jogh-11-15002-s001.pdf]

Appendix S1: Sample search strategy in CINHAL

	Search ID#	Search Terms	Search Options	Actions
	S9	S7 AND S8	Search modes - Boolean/Phrase	**View Results** (1,926)<br>**View Details**<br>**Edit**
	S8	S1 OR S2 OR S3 OR S4 OR S5 OR S6	Search modes - Boolean/Phrase	**View Results** (493,692)<br>**View Details**<br>**Edit**
	S7	(MH "Africa") OR "africa" OR (MH "Africa South of the Sahara") OR (MH "Africa, Western") OR (MH "Africa, Southern") OR (MH "Africa, Northern") OR (MH "Africa, Eastern") OR (MH "Africa, Central") OR (MH "South Africa") OR (MH "Nigeria") OR (MH "Ghana") OR (MH "KENYA") OR (MH "Uganda")	Search modes - Boolean/Phrase	**View Results** (22,777)<br>**View Details**<br>**Edit**
	S6	"older person"	Search modes - Boolean/Phrase	**View Results** (875)<br>**View Details**<br>**Edit**
	S5	(MH "Geriatrics") OR "Gerontology"	Search modes - Boolean/Phrase	**View Results** (4,538)<br>**View Details**<br>**Edit**
	S4	(MH "Aged") OR "aged"	Search modes - Boolean/Phrase	**View Results** (476,124)<br>**View Details**<br>**Edit**
	S3	"senior citizens"	Search modes - Boolean/Phrase	**View Results** (368)<br>**View Details**<br>**Edit**
	S2	"older adults"	Search modes - Boolean/Phrase	**View Results** (29,413)<br>**View Details**<br>**Edit**
	S1	(MH "Aged, 80 and Over") OR (MH "Aged") OR "elderly"	Search modes - Boolean/Phrase	**View Results** (434,318)<br>**View Details**<br>**Edit**

Appendix S2 : Characteristics of included articles: Quantitative (n=426), qualitative (n=71) and mixed method (n=15)

QUANTITATIVE ARTICLES (n=426)			
**Authors (Year), Country**	**Title**	**Study design**	**Sample size**
Abbai et al (2018), South Africa	Good correlation between the Afinion AS100 analyser and the ABX Pentra 400 analyser for the measurement of glycosylated haemoglobin and lipid levels in older adults in Durban, South Africa	Cross-sectional	435
Abeme et al (2020), Nigeria	Blood Pressure Control and Kidney Damage in Hypertension: Results of a Three-Center Cross-Sectional Study in North Central Nigeria	Cross-sectional	1063
Abelson (2013), South Africa	Left atrial appendage closure in patients with atrial fibrillation in whom warfarin is contra-indicated: initial South African experience.	Pre-post (prospective)	12
Aboderin et al (2017), Kenya	Musculoskeletal health conditions among older populations in urban slums in sub-Saharan Africa	Cross-sectional	2006/07 survey - 921, 2016 survey - 1497
Ackuaku dogbe et al (2015), Ghana	Cataract Surgical Uptake Among Older Adults in Ghana	Cross-sectional	5571
Adam et al (2013), South Africa	Effect of pre-fracture mobility on the early post-operative functional outcome in elderly patients with a hip fracture	Pre-post (observational)	90
Adebajo et al (1991), Nigeria	Fractures of the hip and distal forearm in West Africa and the United Kingdom.	Case-control (Retrospective)	746700: Ibadan, 416500: Southampton
Adebusoye et al (2018), Nigeria	Factors Associated With Sarcopenia Among Older Patients Attending a Geriatric Clinic in Nigeria	Cross-sectional	624
Adebusoye et al (2020), Nigeria	Mortality Trends among Older Patients Admitted to the Geriatric Centre, University College Hospital, Ibadan, Nigeria, 2013-2017.	Cross-sectional	1091
Adhvaryu (2007), Tanzania	The Long-Run Impacts of Adult Deaths on Older Household Members in Tanzania	Longitudinal	613
Agboghoroma et al (2020), Nigeria	Peripheral arterial disease and its correlates in patients with type 2 diabetes mellitus in a teaching hospital in northern Nigeria: a cross-sectional study	Cross-sectional	200
Agbozo et al (2018), Ghana	Nutrition knowledge, dietary patterns and anthropometric indices of older persons in four peri-urban communities in Ga West municipality, Ghana	Cross-sectional	120
Agyemang-Duah et al (2020) Ghana	Predictors of healthcare utilisation among poor older people under the livelihood empowerment against poverty programme in the Atwima Nwabiagya District of Ghana	Cross-sectional	200
Aheto et al (2020), Ghana	Prevalence, socio-demographic and environmental determinants of asthma in 4621 Ghanaian adults: Evidence from Wave 2 of the World Health Organization's study on global AGEing and adult health	Cross-sectional	4621

Akande-Sholabi et al (2020), Nigeria	Pharmacists' knowledge and counselling on fall risk increasing drugs in a tertiary teaching hospital in Nigeria	Cross-sectional	56
Akande-Sholabi et al (2020), Nigeria	Evaluation of Prescription Pattern of Analgesic Use among Ambulatory Elderly in South-Western Nigeria	Cross-sectional	337
Akinyemi et al (2008), Nigeria	The Nigerian Aging Males' Symptoms scale. Experience in elderly males	Cross-sectional	456
Akinyemi et al (2014), Nigeria, Sudan & Tanzania	Contribution of Non-communicable Diseases to Medical Admissions of Elderly Adults in Africa: A Prospective, Cross-sectional in Nigeria, Sudan, and Tanzania	Cross-sectional	874
Akinyemi et al (2017), Nigeria	Demographic and epidemiological characteristics of HIV opportunistic infections among older adults in Nigeria	Longitudinal (cohort)	17,312
Akinyemu et al (2014), Nigeria	Profile and Determinants of Vascular Cognitive Impairment in African Stroke Survivors: The CogFAST Nigeria Study	Case-control	217
Akinyemu et al (2015), Nigeria	Medial Temporal Lobe Atrophy, White Matter Hyper-intensities and Cognitive Impairment Among Nigerian African Stroke Survivors	Cross-sectional	58
Akosile et al (2014), Nigeria	Fear of falling and quality of life of apparently-healthy elderly individuals from a Nigerian population.	Cross-sectional	261
Akosile et al (2018), Nigeria	Depression, Functional Disability and Quality of Life Among Nigerian Older Adults: Prevalence and Relationships	Cross-sectional	206
Akor et al (2020), Nigeria	Predictors of Health-Related Quality of Life in patients with Chronic Obstructive Pulmonary Diseases using the COPD assessment test	Cross-sectional	60
Akoria et al (2020), Nigeria	Evaluation of Documentation of Admissions into A Geriatrics Unit in Nigeria: 2014-2018	Cross-sectional	835
Akuamoah-Boateng et al (2013), Ghana	Self-reported Vision Health Status Among Older People in the Kassena-Nankana District, Ghana	Cross-sectional	4294
Alberts et al (1991), South Africa	Metastatic breast cancer - age has a significant effect on survival	Longitudinal (retrospective)	426
Allain et al (2014), Malawi	Falls and other geriatric syndromes in Blantyre, Malawi: a community survey of older adults	Cross-sectional	98
Amegbo et al (2018), Ghana	Predictors of basic self-care and intermediate self-care functional disabilities among older adults in Ghana.	Cross-sectional	4107
Amegbor et al (2020), Ghana	Effect of cognitive and structural social capital on depression among older adults in Ghana: A multilevel cross-sectional analysis	Cross-sectional	4123
Ameh et al (2015), South Africa	Predictors of health care use by adults 50 years and over in a rural South African setting.	Cross-sectional	5795
Amoo et al (2020), Nigeria	Prevalence and Pattern of Psychiatric Morbidity Among Community-Dwelling Elderly Populations in Abeokuta, Nigeria	Not stated	532
Amosun (2014), South Africa	The process of enhancing a geriatric module in undergraduate physiotherapy education in South Africa - perceived attitudes	Cross- sectional	100

	towards ageing among community-dwelling elderly persons in Cape Town		
Amosun et al (2007), South Africa	Are elderly pedestrians allowed enough time at pedestrian crossings in Cape Town, South Africa?	Cross-sectional	60
Annin et al (2014), Ghana	Assessing the association between the degree of pain and socioeconomic status among older persons in Ghana.	Cross-sectional	5108
Ardington et al (2009), South Africa	The impact of AIDS on intergenerational support in South Africa: Evidence from the Cape area panel study.	Longitudinal (retrospective)	696
Awoke et al (2017), Ghana	Predictors of public and private healthcare utilization and associated health system responsiveness among older adults in Ghana.	Cross-sectional	2517
Awuviry-Newton et al (2020), Ghana	Do factors across the World Health Organisation's International Classification of Functioning, Disability and Health framework relate to caregiver availability for community dwelling older adults in Ghana?	Cross-sectional	400
Awuviry-Newton et al (2020), Ghana	Correlates of older adult inpatients' personal care provision to people with functional difficulties in Ghana	Cross-sectional	400
Ayernor et al (2012), Ghana	Disease of Ageing in Ghana	Cross-sectional	507
Ayodapo et al (2020), Nigeria	Patient Education and Medication Adherence among Hypertensives in a Tertiary Hospital, South Western Nigeria	Cross-sectional	420
Ayuk et al (2020), Nigeria	Impact of Diabetes Mellitus on Sexuality in a Developing Country Setting: A Case-Control Study in Calabar, Nigeria	Case-control	330
Balogun et al (2019), Nigeria	Determinants of Bed Net Use Among Older People in Nigeria: Results From a Nationally Representative Survey	Cross-sectional	3586
Bastawrous et al (2016), Kenya	Six-Year Incidence of Blindness and Visual Impairment in Kenya: The Nakuru Eye Disease Cohort Study	Longitudinal (cohort)	5000
Beauge et al (2020), Burkina Faso	Do Targeted User Fee Exemptions Reach the Ultra-Poor and Increase their Healthcare Utilisation? A Panel Study from Burkina Faso	Longitudinal	1260
Bennett et al (2016), Kenya	Gender differentials and old age survival in the Nairobi slums, Kenya.	Longitudinal	2417
Biritwum et al (2013), Ghana	Household characteristics for older adults and study background from SAGE Ghana Wave 1	Longitudinal	6000
Bloomfield et al (2017), Kenya	Markers of Atherosclerosis, Clinical Characteristics, and Treatment Patterns in Heart Failure A Case-Control Study of Middle-Aged Adult Heart Failure Patients in Rural Kenya	Case control	5562
Boateng et al (2017), Ghana	Obesity and the burden of health risks among the elderly in Ghana: A population study	Cross-sectional	2,091

Boon et al (2009), South Africa	The impact of a community-based pilot health education intervention for older people as caregivers of orphaned and sick children as a result of HIV and AIDS in South Africa.	Pre-post	202
Boon et al (2010), South Africa	Correlates of grief among older adults caring for children and grandchildren as a consequence of HIV and AIDS in South Africa.	Cross-sectional	755
Boon et al (2010), South Africa	Explaining perceived ability among older people to provide care as a result of HIV and AIDS in South Africa.	Cross-sectional	409
Bornman et al (2007), South Africa	Serum testosterone levels in South African men and the onset of androgen decline in ageing males	Not stated	40
Brathwaite et al (2002), South Africa	Elderly citizen's perception of their health and care provided in a rural South African community.	Cross-sectional	201
Cadmus et al (2017), Nigeria	A Descriptive Study of the Morbidity Pattern of Older Persons Presenting at a Geriatric Centre in Southwestern Nigeria	Cross-sectional	4886
Calllixte et al (2015), Cameroon	The pattern of neurological diseases in elderly people in outpatient consultations in Sub-Saharan Africa.	Longitudinal (retrospective)	912
Calys-Tagoe (2014), Ghana	Predictors of Subjective Well-Being Among Older Ghanaians	Cross-sectional	4724
Calys-Tagoe et al (2020), Ghana	Individual awareness and treatment effectiveness of hypertension among older adults in Ghana: evidence from the World Health Organization study of global ageing and adult health wave 2	Cross-sectional	3575
Charlton et al (1997), South Africa	The prevalence of diabetes mellitus and associated risk factors in elderly coloured South Africans	Cross-sectional	200
Charlton et al (2007), South Africa	The MNA, but not the DETERMINE, screening tool is a valid indicator of nutritional status in elderly Africans. Nutrition.	Cross-sectional	283
Chepngeno-Langat et al (2011), Kenya	Gender differentials on the health consequences of care-giving to people with AIDS-related illness among older informal carers in two slums in Nairobi, Kenya.	Cross-sectional	1492
Chepngeno-Langat et al (2012), Kenya	Concern About HIV and AIDS Among Older People in the Slums of Nairobi, Kenya	Longitudinal	2,061
Chepngeno-Langat (2013), Kenya	Perception of vulnerability to HIV infection among older people in Nairobi, Kenya: a need for intervention	Longitudinal	2053
Chepngeno-Langat et al (2014), Kenya	Entry and re-entry into informal care-giving over a 3-year prospective study among older people in Nairobi slums, Kenya	Cross-sectional	2696
Chilima et al (1998), Malawi	Anthropometric characteristics of older people in rural Malawi.	Cross-sectional	296
Chilima et al (2001), Malawi	Nutrition and handgrip strength of older adults in rural Malawi	Cross-sectional	296
Chukwuorji et al (2017), Nigeria	Stressful life events, family support and successful ageing in the Biafran War generation.	Cross-sectional	453
Clark et al (2014), Nigeria & USA	Obesity and 10-year mortality in very old African Americans and Yoruba-Nigerians: exploring the obesity paradox.	Longitudinal (cohort)	4698

Clausen (2005), Botswana	Predictors of Food Variety and Dietary Diversity Among Older Persons in Botswana	Cross-sectional	1085
Dake & Welen (2020) Ghana	Towards universal access to healthcare for older adults: an assessment of the old-age exemption policy under Ghana's National Health Insurance Scheme	Cross-sectional	2412
De Jager et al (2017), South Africa	Dementia prevalence in a rural region of South Africa: A Cross-sectional community study.	Cross-sectional	1394
de Picciotto et al (2001), South Africa	Verbal Fluency in Elderly Bilingual Speakers: Normative Data and Preliminary Application to Alzheimer's Disease	Not stated	36
de Rouvray et al (2014), central African Republic and Congo	The nutritional status of older people with and without dementia living in an urban setting in central Africa: the EDAC study	Cross-sectional	1016
De-Terline et al (2020), 12 countries: Benin, Cameroon, Congo (Brazzaville), Democratic Republic of the Congo, Gabon, Guinea, Cote d'ivoire, Mauritania, Mozambique, Niger, Senegal and Togo)	Poor adherence to medication and salt restriction as a barrier to reaching blood pressure control in patients with hypertension: Cross-sectional study from 12 sub-Saharan countries	Cross-sectional	2198
De Villiers et al (2009), South Africa	Efficacy and safety of a live attenuated influenza vaccine in adults 60 years of age and older	Randomized Control Trial	3242
De Villiers et al (2011), South Africa	Stroke outcomes in a socio-economically disadvantaged urban community	Not stated	196
Dei (2018), Ghana	Is healthcare really equal for all? Assessing the horizontal and vertical equity in healthcare utilisation among older Ghanaians	Cross-sectional	4304
Desormais et al (2014), Central African Republic	Prevalence of peripheral artery disease in the elderly population in urban and rural areas of Central Africa: the EPIDEMCA study	Not stated	1871
Dewhurst et al (2012), Tanzania	The prevalence of disability in older people in Hai, Tanzania	Cross-sectional	2232
Dewhurst et al (2012), Tanzania	Rates of diagnosis and treatment of neurological disorders within a prevalent population of community-dwelling elderly people in sub-Saharan Africa	Longitudinal (cohort)	2232
Dewhurst et al (2012), Tanzania	Strikingly Low Prevalence of Atrial Fibrillation in Elderly Tanzanians	Cross-sectional	2232
Dewhurst et al (2013), Tanzania	The prevalence of neurological disorders in older people in Tanzania	Cross-sectional	2232
Dewhurst et al (2013), Tanzania	The high prevalence of hypertension in rural-dwelling Tanzanian older adults and the disparity between detection, treatment and control: a rule of sixths?	Longitudinal (cohort)	2232
Dewhurst et al (2014)	Electrocardiographic Reference Values for a Population of Older Adults in Sub-Saharan Africa	Cross-sectional	2232
Dia et al (2014), Senegal	Respiratory viruses associated with patients older than 50 years presenting with ILI in Senegal, 2009 to 2011	Longitudinal (cohort)	232

Diamond et al (1986), South Africa	Primary hyperparathyroidism, A study of 100 patients in Johannesburg	Case-control	100
Digenio et al (1991), South Africa	The Johannesburg cardiac rehabilitation programme	Longitudinal	387
Dobseu et al (2020), Cameroon	Evaluation of hepatic fibrosis in HIV/HCV co-infected individuals in Yaoundé, Cameroon: usefulness of APRI score in resource-constrained settings	Cross-sectional	97
Dotchin et al (2014), Tanzania	The association between disability and cognitive impairment in an elderly Tanzanian population	Longitudinal (cohort)	296
du Rand et al (2001), South Africa	Needs of frail elderly people in informal settlements	Cross-sectional	1200
Eales et al (1996), South Africa	The exercise capacity of three socio-economic groups of elderly hypertensive patients	Not stated	44
Eales et al (1997), South Africa	A Proposed Field Test for Evaluating Fitness in Elderly Hypertensive Patients	Cross-sectional	44
Eduardo et al (2014), Kenya, Mozambique, Rwanda & Tanzania	Characteristics and outcomes among older HIV-positive adults enrolled in HIV programs in four sub-Saharan African countries.	Longitudinal (cohort)	HIV care - 392, 131 ART - 184,689
Elk et al (1983), South Africa	The Coloured elderly in Cape - a psychosocial, psychiatric and medical community survey	Cross-sectional	150
Enikuomehin et al (2020), Nigeria	Influence of gender on the distribution of type 2 diabetic complications at the Obafemi Awolowo teaching hospital, Ile-Ife, Nigeria	Cross-sectional	400
Eze et al. (2020), Nigeria	Anterior Bladder Wall Thickness, Post-Void Urine Residue, and Bladder Emptying Efficiency as Indicators of Bladder Dysfunction in Nigerian Men with Benign Prostatic Hyperplasia	Cross-sectional	77
Ezenwa et al (2020), Nigeria	Apical Peri-prostatic Nerve Block versus Intra-rectal Xylocaine Gel for Trans-.Rectal Ultrasound Guided Prostate Biopsy among Nigerian Patients: A prospective Randomized Study	Randomized control trails	90
Faber et al (1992), South Africa	Dietary patterns and nutritional status in free-living older white men with established vascular disease	Not stated	39
Fakoya et al (2018), Nigeria	Determinants of Quality of Life of Elderly Patients Attending a General Practice Clinic in Southwest Nigeria.	Cross-sectional	216
Fantahun et al (2009), Ethiopia	Ageing of a Rural Ethiopian Population: Who Are the Survivors?	Longitudinal (cohort)	2231
Fawale et al (2017), Nigeria	Correlates of sleep quality and sleep duration in a sample of urban-dwelling elderly Nigerian women	Cross-sectional	528
Ferrari et al (2013), South Africa	Geographical variations in the prevalence and management of cardiovascular risk factors in outpatients with CAD: Data from the contemporary CLARIFY registry	Longitudinal (cohort)	32,954
Folorunso et al (2020), Nigeria	Age-Related Hearing Loss at Gwagwalada Area Council of Federal Capital Territory, Abuja	Cross-sectional	114

Forrest et al (2011), South Africa & USA	A prospective, randomized, open-label trial comparing the safety and efficacy of trivalent live attenuated and inactivated influenza vaccines in adults 60 years of age and older	Randomized “open label” trial	3009
Gatimu et al (2016), Ghana	Prevalence and determinants of diabetes among older adults in Ghana	Cross-sectional	5565
Gaziano et al (2017), South Africa	Cardio metabolic risk in a population of older adults with multiple co-morbidities in rural South Africa: the HAALSI (Health and Aging in Africa: Longitudinal studies of INDEPTH communities) study	Longitudinal (cohort)	6821
Geerts (2017), South Africa	Neutral zone or conventional mandibular complete dentures: a randomised crossover trial comparing oral health-related quality of life	Randomized “crossover” trial	35
Gildner et al. (2014), China, Ghana, India, Mexico, Russian & South Africa	Sleep duration, sleep quality, and obesity risk among older adults from six middle-income countries: findings from the study on global AGEing and adult health (SAGE).	Cross-sectional	28,980
Gillis et al (1981), South Africa	Physical and mental incapacity in elderly white persons in Cape Town	Cross-sectional	150
Gillis et al (1991), South Africa	Psychological distress and depression in urbanising elderly black persons	Cross-sectional	365
Gomez-Olivé (2014), South Africa	Sleep problems and mortality in rural South Africa: novel evidence from a low-resource setting.	Cross-sectional	6206
Gómez-Olivé et al (2010), South Africa	Assessing health and well-being among older people in rural South Africa	Cross-sectional	6206
Gómez-Olivé et al (2013), South Africa	Self-reported health and health care use in an ageing population in the Agincourt sub-district of rural South Africa	Cross-sectional	425
Gomez-Olive et al (2014), South Africa	Social conditions and disability related to the mortality of older people in rural South Africa.	Longitudinal (cohort)	4085
Govender et al (2010), South Africa	The Health Status and Unmet Health Needs of Old-Age Pensioners Living in Selected Urban Poor Communities in Cape Town, South Africa	Cross-sectional	703
Gray et al (2014), Tanzania	Development and Validation of the Identification and Intervention for Dementia in Elderly Africans (IDEA) Study Dementia Screening Instrument	Longitudinal (cohort)	1198
Gray et al (2016), Tanzania	Rates and predictors of three-year mortality in older people in rural Tanzania	Longitudinal (cohort)	2232
Gray et al (2017), Tanzania	Identifying Frailty and its Outcomes in Older People in Rural Tanzania	Longitudinal (cohort)	1198
Guerchet (2009), Benin Republic	Cognitive Impairment and Dementia in Elderly People Living in Rural Benin, West Africa	Cross-sectional	502

Guerchet et al (2010), Central African Republic	Prevalence of Dementia in Elderly Living in Two Cities of Central Africa: The EDAC Survey	Cross-sectional	1016
Guerchet et al (2012), Central African Republic and Congo	Factors Associated with Dementia Among Elderly People Living in Two Cities in Central Africa: The EDAC Multi-center Study	Cross-sectional	1016
Guerchet et al (2013), Central African Republic	Association Between a Low Ankle–Brachial Index and Dementia in a General Elderly Population in Central Africa (Epidemiology of Dementia in Central Africa Study)	Cross-sectional	1055
Gureje et al (2006), Nigeria	Functional Disability in Elderly Nigerians: Results from the Ibadan Study of Aging	Cross-sectional	2152
Gureje et al (2006), Nigeria	The profile and impact of probable dementia in a sub-Saharan African community: results from the Ibadan Study of Aging	Cross-sectional	2152
Gureje et al (2007), Nigeria	Epidemiology of major depressive disorder in elderly Nigerians in the Ibadan Study of Ageing: a community-based survey	Cross-sectional	2152
Gureje et al (2008), Nigeria	Depression and disability: comparisons with common physical conditions in the Ibadan study of aging.	Longitudinal	2152
Gureje et al (2011), Nigeria	The Natural History of Insomnia in the Ibadan Study of Ageing	Longitudinal	1696
Gureje et al (2011), Nigeria	Incidence of and risk factors for dementia in the Ibadan study of aging.	Longitudinal (cohort)	2149 (Follow-up=1408)
Gureje et al (2014), Nigeria	Profile and Determinants of Successful Aging in the Ibadan Study of Ageing	Longitudinal	2149
Gutierrez et al (2012), Angola	Perception of quality of life in an elderly Angolan sample	Cross-sectional	1003
Gyasi et al (2018), Ghana	Gender, Self-Rated Health and Functional Decline Among Community-Dwelling Older Adults	Cross-sectional	1200
Gyasi et al (2019) Ghana	Financial Inclusion, Health-Seeking Behavior, and Health Outcomes Among Older Adults in Ghana	Longitudinal	1200
Gyasi et al (2020), Ghana	Association of food insecurity with psychological disorders: Results of a population-based study among older people in Ghana	Cross-sectional	1200
Gyasi et al (2020), Ghana	How do lifestyle choices affect the link between living alone and psychological distress in older age? Results from the AgeHeaPsyWel-HeaSeeB study	Cross-sectional	1200
Gyasi et al (2020), Ghana	Impact of food insecurity with hunger on mental distress among community-dwelling older adults	Cross-sectional	1200
Gyasi et al (2020), Ghana	Multidimensional Social Support and Health Services Utilization Among Noninstitutionalized Older Persons in Ghana	Cross-sectional	1200
Gyasi et al (2020), Ghana	Physical activity and predictors of loneliness in community-dwelling older adults: The role of social connectedness	Cross-sectional	1200
Gyasi et al (2020), Ghana	Risk of Psychological Distress Among Community- Dwelling Older Adults Experiencing Spousal Loss in Ghana	Cross-sectional	1200

Hao et al (2017), South Africa	Social participation and perceived depression among elderly population in south Africa	Cross-sectional	422
Hendrie et al (2013), Nigeria	Homocysteine levels and dementia risk in Yoruba and African Americans	Longitudinal (cohort)	760
Heyns et al (2003), South Africa	Problems with prostate specific antigen screening for prostate cancer in the primary healthcare setting in South Africa	Not stated	21
Heyns et al (2011), South Africa	Prostate cancer among different racial groups in the Western Cape: Presenting features and management	Longitudinal (retrospective)	901
Hien (2014), Burkina Faso	Prevalence and patterns of multi-morbidity among the elderly in Burkina Faso: Cross-sectional	Cross-sectional	389
Hontelez et al (2011), South Africa	Ageing with HIV in South Africa	Cross-sectional (mathematical model)	Not applicable
Hosegood et al (2005), South Africa	The impact of adult mortality on the living arrangements of older people in rural South Africa.	Longitudinal	3,657
Houser et al (2017), DR Congo	Diabetes and Hypertension in Congolese Church Personnel: An Emerging Epidemic?	Cross-sectional	670
Huang et al (2020), Ghana & South Africa	Effect of financial stress on self-rereported health and quality of life among older adults in five developing countries: a cross sectional analysis of WHO-SAGE survey	Cross-sectional	Ghana= 1968<br>South Africa= 1924
Hughes et al (2013), South Africa	The prevalence of traditional herbal medicine use among hypertensives living in South African communities.	Cross-sectional	135
Ibrahim et al (2015), Nigeria	Direct non-medical costs double the total direct costs to patients undergoing cataract surgery in Zamfara state, Northern Nigeria: a case series.	Cross-sectional	104
Ice et al (2008), Kenya	Health and health perceptions among Kenyan grandparents.	Longitudinal	287
Ice et al (2010), Kenya	The impact of caregiving on the health and well-being of Kenyan Luo grandparents.	Longitudinal	1072
Ice et al (2012), Kenya	Stress associated with caregiving: An examination of the stress process model among Kenyan Luo elders	Longitudinal	640
Igbokwe et al (2020), Nigeria	Prevalence of loneliness and association with depressive and anxiety symptoms among retirees in Northcentral Nigeria: a cross-sectional study	Cross-sectional	1099
Jacobs et al (1984), South Africa	The coloured elderly in Cape Town--a psychosocial, psychiatric and medical community survey. Part IV. Haematological values.	Cross-sectional	150
Jardim et al (2018), South Africa	Cardiovascular Disease Profile of the Oldest Adults in Rural South Africa: Data from the HAALSI Study (Health and Aging in Africa: Longitudinal Studies of INDEPTH Communities).	Longitudinal	5059

Jésus et al (2013), Central African Republic & Republic of Congo	Factors associated with nutritional status in elderly living in two cities of central Africa: The EDAC study	Cross-sectional	1016
Joffe et al (1975), South Africa	Metabolic bone disease in the elderly. Biochemical studies in three different racial groups living in South Africa.	Cross-sectional	60
Kailemobi et al (2016), Ghana & South Africa	Common risk factors and edentulism in adults, aged 50 years and over, in China, Ghana, India and South Africa: results from the WHO Study on global AGEing and adult health (SAGE).	Cross-sectional	China 11,692, Ghana 4093), India 6409 & South Africa 2985
Kalu et al (2019), Nigeria	Knowledge about risk factors for falls and practice about fall prevention in older adults among physiotherapists in Nigeria.	Cross-sectional	329
Kalula (2015), South Africa	Prevalence of falls in an urban community-dwelling older population of Cape Town, South Africa	Cross-sectional	837
Kalula et al (2006), South Africa	Management of older patients presenting after a fall - an accident and emergency department audit.	Longitudinal	100
Kalula et al (2010), South Africa	Profile and management of patients at a memory clinic.	Longitudinal	305
Kalula et al (2015), South Africa	Ethnic differences in rates and causes of falls in an urban community-dwelling older population in South Africa.	Longitudinal	837
Kalula et al (2016), South Africa	Risk factors for falls in older adults in a South African Urban Community.	Longitudinal	837
Kalula et al (2017), South Africa	Methodological challenges in a study on falls in an older population of Cape Town, South Africa	Longitudinal	837
Karstaedt et al (2014), South Africa	Tuberculosis in older adults in Soweto, South Africa.	Longitudinal (cohort)	110
Kellett-Wright et al (2020), Tanzania	Screening for HIV-Associated Neurocognitive Disorder (HAND) in Adults Aged 50 and Over Attending a Government HIV Clinic in Kilimanjaro, Tanzania. Comparison of the International HIV Dementia Scale (IHDS) and IDEA Six Item Dementia Screen	Cross-sectional of a longitudinal study	253
Kimuna et al (2007), South Africa	Older People as Resources in South Africa: Mpumalanga Households	Cross-sectional	1002
Kinyanda et al (2016), Uganda	Risk of major depressive disorder among older persons living in HIV-endemic central and southwestern Uganda.	Cross-sectional	471
Klemz et al (2015), South Africa	The effect of altruism on the spending behavior of elderly caregivers of family members with HIV/AIDS in South African townships.	Cross-sectional	50
Kobayashi et al (2019), South Africa	Socioeconomic gradients in chronic disease risk behaviors in a population-based study of older adults in rural South Africa	Cross-sectional	5059
Kobayashi et al (2019), South Africa	Cognitive Function and Impairment in Older, Rural South African Adults: Evidence From "Health and Aging in Africa: A Longitudinal Study of an INDEPTH Community in Rural South Africa"	Longitudinal	5059
Kolbe-Alexander et al (2006), South Africa	Comparison of two methods of measuring physical activity in South African older adults	Cross-sectional	122

Kolbe-Alexander et al (2015), South Africa	The relationship between the built environment and habitual levels of physical activity in South African older adults: a pilot study.	Cross-sectional	44
Koyanagi et al (2019), South Africa	Food Insecurity Is Associated with Mild Cognitive Impairment among Middle-Aged and Older Adults in South Africa: Findings from a Nationally Representative Survey.	Cross-sectional	3672
Kretchy et al (2020), Ghana	The Association between Diabetes-Related Distress and Medication Adherence in Adult Patients with Type 2 Diabetes Mellitus: A Cross-Sectional Study	Cross-sectional	188
Krige (2010), South Africa	Melanoma in black South Africans.	Longitudinal (retrospective)	63
Kunna et al (2017), China & Ghana	Measurement and decomposition of socioeconomic inequality in single and multi-morbidity in older adults in China and Ghana: results from the WHO study on global AGEing and adult health (SAGE)	Cross-sectional	13,177 in China, 4305 in Ghana
Kyobuntungi (2010), Kenya	The health and well-being of older people in Nairobi's slums.	Cross-sectional	2696
Kyobutungi et al (2009), Kenya	HIV/AIDS and the health of older people in the slums of Nairobi, Kenya: results from a Cross-sectional survey	Cross-sectional	2696
Lambert et al (2017), Ghana, India & Russian	Impact of informal caregiving on older adults' physical and mental health in low-income and middle-income countries: a cross-sectional, secondary analysis based on the WHO's Study on global AGEing and adult health (SAGE).	Cross-sectional	caregivers (Ghana 143; India 490; Russia 270) & non-caregivers (Ghana 4112; India 6001; Russia 3304)
Lartey et al (2019), Ghana	Rapidly Increasing Prevalence of Overweight and Obesity in Older Ghanaian Adults From 2007-2015: Evidence From WHO-SAGE Waves 1 & 2	Longitudinal	5821
Lasisi et al (2010), Nigeria	Tinnitus in the elderly: Profile, correlates, and impact in the Nigerian Study of Ageing.	Longitudinal (cohort)	2152
Lasisi et al (2014), Nigeria	Prevalence and correlates of dizziness in the Ibadan Study of Ageing	Longitudinal (cohort)	2152
Lazenby et al (2019), Botswana	Place of death among Botswana's oldest old.	Longitudinal	26594
Le Roux et al (2007), South Africa	Acute extradural haematoma in the elderly.	Longitudinal	3249
Lekpa et al (2013), Senegal	Socio-demographic and clinical profile of chronic pain with neuropathic characteristics in sub-Saharan African elderly.	Cross-sectional	549
Lenger et al (1996), South Africa	Informant questionnaires as screening measures to detect dementia. A pilot study in the South African context.	Cross-sectional	40
Lewis et al (2017), Tanzania	Risk factors for delirium in older medical inpatients in Tanzania.	Cross-sectional	510
Longdon et al (2013), Tanzania	The prevalence of dementia in rural Tanzania: A Cross-sectional community-based study.	Cross-sectional	1198

Lowis et al (1997), South Africa	A comparison of the effects of sacred and secular music on elderly people.	Cross-sectional	30
Lwanga et al (2020), Uganda	Implementing routine physical function screening among elderly HIV-positive patients in Uganda	Cross-sectional	93
Mabaso et al (2016), South Africa	Knowledge and practices related to diabetes mellitus among adults with diabetes in the Mopani District, Limpopo Province, South Africa	Cross-sectional	225
Mabeku et al (2020), Cameroon	Helicobacter pylori infection, a risk factor for Type 2 diabetes mellitus: a hospital-based cross-sectional study among dyspeptic patients in Douala-Cameroon	Cross-sectional	187
Macia et al (2012), Senegal	Prevalence, awareness, treatment and control of hypertension among adults 50 years and older in Dakar, Senegal	Cross-sectional	500
Macia et al (2012), Senegal	Age identity, self-rated health, and life satisfaction among older adults in Dakar, Senegal	Cross-sectional	500
Macia et al (2015), South Africa	Exploring life satisfaction among older adults in Dakar.	Cross-sectional	500
Manne-Goehler et al (2019), Uganda	Depressive symptoms before and after antiretroviral therapy initiation among older-aged individuals in rural Uganda	Longitudinal (cohort)	154 PLWH and 142 community-based HIV-negative controls
Maritz et al (2018), South Africa	Evaluating several biomarkers as predictors of aortic stiffness in young and older Africans, not consuming alcohol based on self-report.	Cross-sectional	322
Martinez et al (2012), South Africa	Quality of life and social engagement of alcohol abstainers and users among older adults in South Africa.	Cross-sectional	3666
Matlho et al (2019),Botswana	HIV prevalence and related behaviours of older people in Botswana — secondary analysis of the Botswana AIDS Impact Survey (BAIS) IV	Cross-sectional	970
Mbada et al (2020), Nigeria	Cross-cultural adaptation and psychometric evaluation of the Yoruba version of the Back beliefs questionnaire among patients with chronic low-back pain	Cross-sectional	119
Mbui et al (2017), Kenya	Prescription patterns and adequacy of blood pressure control among adult hypertensive patients in Kenya; findings and implications	Cross-sectional	271
McKinnon et al (2013), Sub-Saharan African	The relationship of living arrangements and depressive symptoms among older adults in sub-Saharan Africa.	Cross-sectional	12,647
Meiring et al (1983), South Africa	Identification and definition of the geriatric patient in a teaching hospital.	Case control	100
Menyanu et al (2017), Ghana & South Africa	Salt Use Behaviours of Ghanaians and South Africans: A Comparative Study of Knowledge, Attitudes and Practices	Longitudinal	10,522

Mhaka-Mutepfa et al (2014), Uganda	Grandparents fostering orphans: Influences of protective factors on their health and well-being.	Cross-sectional	241
Minicuci et al (2014), Ghana	Sociodemographic and socioeconomic patterns of chronic non-communicable disease among the older adult population in Ghana.	Cross-sectional	4274
Molete et al (2014), South Africa	Oral health needs and barriers to accessing care among the elderly in Johannesburg.	Cross-sectional	308
Moreono-Agostino et al (2020), Ghana and South Africa	Are Retired People Higher in Experiential Wellbeing Than Working Older Adults? A Time Use Approach	Longitudinal	Ghana= 2598<br>South Africa=1589
Mtowa et al (2017), Tanzania	Socio-demographic inequalities in HIV testing behaviour and HIV prevalence among older adults in rural Tanzania, 2013.	Cross-sectional	1643
Mugisha et al (2015), Uganda	Gender perspectives in care provision and care receipt among older people infected and affected by HIV in Uganda	Cross-sectional	510
Mugisha et al (2016), Uganda	Chronic disease, risk factors and disability in adults aged 50 and above living with and without HIV: findings from the Wellbeing of Older People Study in Uganda	Cross-sectional	471
Mugisha et al (2016), Uganda	Timing of Most Recent Health Care Visit by Older People Living With and Without HIV: Findings From the SAGE Well-Being of Older People Study in Uganda	Cross-sectional	510
Mugisha et al (2018), Uganda	Social engagement and survival in people aged 50 years and over living with HIV and without HIV in Uganda: a prospective cohort study.	Cross-sectional	510
Mwanyangala et al (2010), Tanzania	Health Status and Quality of Life Among Older Adults in Rural Tanzania	Cross-sectional	8206
Myroniuk (2017), Malawi	Marital Dissolutions and the Health of Older Individuals in a Rural African Context	Cross-sectional	1200
Namale et al (2020), Uganda	Predictors of 30-day and 90-day mortality among hemorrhagic and ischemic stroke patients in urban Uganda: prospective hospital-based cohort study	Longitudinal (cohort)	153
Nanji et al (2020), Kenya	The Muranga Teleophthalmology Study: A Comparison of Virtual (Teleretina) Assessment with in-person Clinical Examination to Diagnose Diabetic Retinopathy and Age-related Macular Degeneration in Kenya	Not stated	612
Nash et al (1983), South Africa	The coloured elderly in Cape Town - a psychosocial, psychiatric and medical community survey. Part III. A survey of physical disorders and disabilities.	Cross-sectional	149
Negin et al (2010), Kenya	High Rates of AIDS-Related Mortality Among Older Adults in Rural Kenya	Longitudinal (retrospective)	1228

Negin et al (2011), Malawi, Rwanda & Tanzania	Risk factors for non-communicable diseases among older adults in rural Africa	Cross-sectional	654
Negin et al (2012), Malawi, Nigeria, Senegal, Rwanda & Tanzania	HIV attitudes, awareness and testing among older adults in Africa.	Cross-sectional	2265
Negin et al (2012), South Africa	Prevalence of HIV and chronic comorbidities among older adults.	Cross-sectional	4227
Negin et al (2016), Uganda	Sexual behaviour of older adults living with HIV in Uganda	Cross-sectional	101
Negin et al (2017), South Africa	Health expenditure and catastrophic spending among older adults living with HIV.	Cross-sectional	2872
Njemini et al (2002), Cameroon	The prevalence of autoantibodies in an elderly sub-Saharan African population	Cross-sectional	331
Njemini et al (2011), Cameroon	Circulating heat shock protein 70 (Hsp70) in elderly members of a rural population from Cameroon: association with infection and nutrition.	Cross-sectional	137
Nwankwo et al (2020), Nigeria	Impact of neoadjuvant chemotherapy in improving operative intervention in the management of cervical cancer in low resource setting: a preliminary report	Pre-post design	20
Nyanguru et al (2007), Uganda	Migration and Aging: the Case of Zimbabwe	Cross-sectional	812
Nyirenda et al (2012), South Africa	An investigation of factors associated with the health and well-being of HIV-infected or HIV-affected older people in rural South Africa.	Cross-sectional	422
Nyirenda et al (2015), Uganda & South Africa	Health, wellbeing, and disability among older people infected or affected by HIV in Uganda and South Africa	Longitudinal (cohort)	932
Obuku et al (2013), Uganda	Determinants of clinician knowledge on aging and HIV/AIDS: a survey of practitioners and policy makers in Kampala District, Uganda.	Cross-sectional	100
Ogunyemi et al (2018), Nigeria	Health-Related Quality of Life of the Elderly in Institutional Care and Non-Institutional Care in Southwestern Nigeria: A Comparative Study.	Cross-sectional	360
Ojagbemi et al (2013), Nigeria	Suicidal behaviour in old age-result from the Ibadan Study of Ageing	Cross-sectional	2149
Ojagbemi et al (2015), Nigeria	Gait speed and cognitive decline over 2 years in the Ibadan study of aging.	Longitudinal (cohort)	2149
Ojagbemi et al (2016), Nigeria	Cognitive Reserve, Incident Dementia, and Associated Mortality in the Ibadan Study of Ageing.	Longitudinal	2149
Ojagbemi et al (2017), Nigeria	Chronic Conditions, New Onset, and Persistent Disability in the Ibadan Study of Aging	Longitudinal (cohort)	2873
Ojagbemi et al (2017), Nigeria	Low Socioeconomic Position, and Mortality in the Ibadan Study of Aging.	Longitudinal (cohort)	2149

Ojagbemi et al (2018), Nigeria	Symptomatic and Functional Recovery From Major Depressive Disorder in the Ibadan Study of Ageing.	Longitudinal (cohort)	2149
Okoye et al (2020), Nigeria	Validation of Igbo version of the modified falls efficacy scale among community dwelling older adults: a validation study	Cross-sectional	109
Okunade et al (2020), Nigeria	Risk predictors of early recurrence in women with epithelial ovarian cancer in Lagos, Nigeria	Longitudinal (cohort)	81
Oladeji et al (2011), Nigeria	Chronic pain conditions and depression in the Ibadan Study of Ageing.	Cross-sectional	2152
Olamoyegun et al (2020), Nigeria	Mobile phone ownership and willingness to receive mHealth services among patients with diabetes mellitus in South-West, Nigeria	Cross-sectional	259
Olatayo et al (2015), Nigeria	Sexuality in Nigerian Older Adults	Cross-sectional	100
Ologe et al. (2005), Nigeria	Ear diseases in elderly hospital patients in Nigeria.	Longitudinal (retrospective)	320
Omenai et al (2020), Nigeria	Mortality Patterns in Patients with Diabetes Mellitus at a Nigerian Tertiary Hospital: A 10-Year Autopsy Study	Cross-sectional	1092
Onadja et al (2013), Burkina Faso	Gender differences in cognitive impairment and mobility disability in old age: A Cross-sectional in Ouagadougou, Burkina Faso	Cross-sectional	918
Onakpoya et al (2020), Nigeria	Early experience with permanent pacemaker implantation at a tertiary hospital in Nigeria	Longitudinal (cohort)	22
Onakpoya et al (2020), Nigeria	Intraocular pressure variation after conventional extracapsular cataract extraction, manual small incision cataract surgery and phacoemulsification in an indigenous black population	Cross-sectional	82
Onwubiko et al (2020), Nigeria	Factors Associated with Depression and Anxiety among Glaucoma Patients in a Tertiary Hospital South-East Nigeria	Cross-sectional	182
Onwuchewa et al (2009), Nigeria	Stroke at the University of Port Harcourt Teaching Hospital, Rivers State, Nigeria	Longitudinal (retrospective)	202
Osberg et al (2015), Tanzania	The Hunger of Old Women in Rural Tanzania: can Subjective Data Improve Poverty Measurement?	Not stated	502
Oshi et al (2014), Nigeria	Profile and Treatment Outcomes of Tuberculosis in the Elderly in South-eastern Nigeria, 2011-2012	Longitudinal (cohort)	212
Ottie-Boakye (2020), Ghana	Coverage of non-receipt of cash transfer (Livelihood Empowerment Against Poverty) and associated factors among older persons in the Mampong Municipality, Ghana – a quantitative analysis	Cross-sectional	356
Otitoola et al (2015), South Africa	Trends in the development of obesity in elderly day care attendees in Sharpeville, South Africa, from 2007-2011.	Longitudinal (cohort)	208
Padayachey et al (2017), South Africa	Depression in older adults: prevalence and risk factors in a primary health care sample	Cross-sectional	255

Paddick et al (2015), Tanzania	The prevalence and burden of behavioural and psychological symptoms of dementia in rural Tanzania.	Cross-sectional	296
Paddick et al (2017), Tanzania	Adaptation and validation of the Alzheimer's Disease Assessment Scale-Cognitive (ADAS-Cog) in a low-literacy setting in sub-Saharan Africa.	Cross-sectional	66
Paddick et al (2018), Tanzania	Identification of delirium and dementia in older medical inpatients in Tanzania: A comparison of screening and diagnostic methods.	Longitudinal (cohort)	507
Paquissi (2016), Angola	Prevalence of Peripheral Arterial Disease among Adult Patients Attending Outpatient Clinic at a General Hospital in South Angola	Cross-sectional	115
Parag et al (2016), South Africa	How long are elderly patients followed up with mammography after the diagnosis of breast cancer? A single-centre experience in a developing country.	Longitudinal (retrospective)	40
Parmar et al (2014), Ghana and Senegal	Enrolment of older people in social health protection programs in West Africa – Does social exclusion play a part?	Cross-sectional	Ghana 435, Senegal 2933
Payne et al (2013), Malawi	Disability Transitions and Health Expectancies among Adults 45 Years and Older in Malawi: A Cohort-Based Model	Longitudinal (cohort)	1665
Payne et al (2017), South Africa	Physical function in an aging population in rural South Africa: Findings from HAALSI and cross-national comparisons with HRS sister studies.	Longitudinal	5059
Payne et al (2017), South Africa	Prevalence and correlates of frailty in an older rural African population: findings from the HAALSI cohort study	Longitudinal	5059
Peil et al (1988), Nigeria	Going home: migration careers of southern Nigerians.	Cross-sectional	1665
Peltzer (2012), South Africa	Sociodemographic and health correlates of sleep problems and duration in older adults in South Africa.	Cross-sectional	3840
Peltzer (2017), South Africa	Differences in Sleep Duration among Four Different Population Groups of Older Adults in South Africa	Cross-sectional	3284
Peltzer et al (2012), South Africa	Tobacco use and associated factors in older adults in South Africa.	Cross-sectional	3840
Peltzer et al (2012), South Africa	Patient experiences and health system responsiveness among older adults in South Africa.	Cross-sectional	3840
Peltzer et al (2012), South Africa	Fruit and vegetable intake and associated factors in older adults in South Africa.	Cross-sectional	3840
Peltzer et al (2012), South Africa	Cognitive functioning and associated factors in older adults in South Africa	Cross-sectional	3840
Peltzer et al (2013), South Africa	Problem drinking and associated factors in older adults in South Africa.	Cross-sectional	2144
Peltzer et al (2013), South Africa	Hypertension and associated factors in older adults in South Africa.	Cross-sectional	3840
Peltzer et al (2013), South Africa	Depression and associated factors in older adults in South Africa.	Cross-sectional	3840

Peltzer et al (2013), South Africa	Arthritis and Associated Factors in Older Adults in South Africa	Cross-sectional	3840
Peltzer et al (2014), South Africa	Breast and Cervical Cancer Screening and Associated Factors among Older Adult Women in South Africa	Cross-sectional	3840
Peltzer et al (2017), South Africa	Association between Visual Impairment and Low Vision and Sleep Duration and Quality among Older Adults in South Africa.	Cross-sectional	3840
Peltzer et al (2018), South Africa	Self-Reported Sleep Duration and Its Correlates with Socio-demographics, Health Behaviours, Poor Mental Health, and Chronic Conditions in Rural Persons 40 Years and Older in South Africa	Cross-sectional	6281
Pengpid et al (2019), South Africa	Sedentary Behaviour and 12 Sleep Problem Indicators Among Middle-Aged and Elderly Adults in South Africa	Cross-sectional	4782
Perold et al (2000), South Africa	The composition of old age homes in South Africa in relation to the residents and nursing personnel	Cross-sectional	612
Pettifor et al (1978), South Africa	Seasonal variation in serum 25-hydroxycholecalciferol concentrations in elderly South African patients with fractures of femoral neck.	Cross-sectional	232
Phaswana-Mafuya (2018), South Africa	Racial or Ethnic Health Disparities among Older Adults in Four Population Groups in South Africa	Cross-sectional	3284
Phaswana-Mafuya et al (2013), South Africa	Self-reported prevalence of chronic non-communicable diseases and associated factors among older adults in South Africa	Cross-sectional	3840
Phaswana-Mafuya et al (2013), South Africa	Sociodemographic predictors of multiple non-communicable disease risk factors among older adults in South Africa.	Cross-sectional	3,840
Phukubye et al (2011), South Africa	The incidence and structure of the fabella in a South African cadaver sample.	Cross-sectional	51
Pieterse et al (2002), Tanzania	The association between nutritional status and handgrip strength in older Rwandan refugees	Cross-sectional	828
Pileron et al (2018), Sub-Saharan Africa	Cancer incidence in older adults in selected regions of sub-Saharan Africa, 2008–2012	Longitudinal (retrospective)	8944
Pilleron et al (2015), Central African Republic	Is dependent personality disorder associated with mild cognitive impairment and dementia in Central Africa? A result from the EPIDEMCA programme.	Cross-sectional	2002
Pilleron et al (2015), Central African Republic	Association between stressful life events and cognitive disorders in Central Africa: Results from the EPIDEMCA program.	Cross-sectional	2002
Pilleron et al (2015), Central African Republic	Association between mild cognitive impairment and dementia and undernutrition among elderly people in Central Africa: some results from the EPIDEMCA (Epidemiology of Dementia in Central Africa) programme	Cross-sectional	2002
Pilleron et al (2017), Central African Republic	Prevalence, awareness, treatment, and control of hypertension in older people in Central Africa: the EPIDEMCA study	Cross-sectional	2113

Prinsloo et al (1991), South Africa	Health services--needs of the elderly in two black urban areas of the Cape Peninsula.	Cross-sectional	365
Puckree et al (1997), South Africa	An evaluation of the functional status of the residents of a geriatric residential facility in South Africa.	Cross-sectional	101
Puckree et al (2002), South Africa	African traditional healers: what health care professionals need to know	Cross-sectional	330
Puckree et al (2014), South Africa	Effectiveness of a community based programme of physiotherapy on stability, balance and function of stroke patients	Longitudinal (cohort)	25
Pupwe et al (2020), South Africa	Chemotherapy for elderly colorectal cancer patients at a tertiary hospital in South Africa	Longitudinal (cohort)	50
Putnam et al (2018), Tanzania	Hypertension in a resource-limited setting: Is it associated with end organ damage in older adults in rural Tanzania?	Longitudinal	246
Raal et al (2011), South Africa	CEPHEUS SA: a South African survey on the under-treatment of hypercholesterolemia.	Cross-sectional	3001
Raal et al (2013), South Africa	Prevalence of dyslipidaemia in statin-treated patients in South Africa: results of the Dyslipidaemia International Study (DYSIS).	Cross-sectional	1029
Rabie et al (2016), South Africa	Relation of socio-economic status to the independent application of self-care in older persons of South Africa	Cross-sectional	198
Ralston et al (2015), South Africa	Who Benefits - Or Does not - From South Africa's Old Age Pension? Evidence from Characteristics of Rural Pensioners and Non-Pensioners.	Cross-sectional	4915
Ralston et al (2018), South Africa	The Role of Older Persons' Environment in Aging Well: Quality of Life, Illness, and Community Context in South Africa	Cross-sectional	2,937
Ralston et al (2019), South Africa	Policy Shift: South Africa's Old Age Pensions' Influence on Perceived Quality of Life	Longitudinal	6025
Ramjeeth et al (2008), South Africa	The evaluation of low-density lipoprotein cholesterol goals achieved in patients with established cardiovascular disease and/or hyperlipidaemia receiving lipid-lowering therapy: the South African Not at Goal study (SA-NAG).	Cross-sectional	1201
Ramlagan et al (2013), South Africa	Social capital and health among older adults in South Africa.	Cross-sectional	3840
Ramlagan et al (2014), South Africa	Hand grip strength and associated factors in non-institutionalised men and women 50 years and older in South Africa.	Cross-sectional	3840
Ramlall et al (2014), South Africa	Sensitivity and specificity of neuropsychological tests for dementia and mild cognitive impairment in a sample of residential elderly in South Africa.	Longitudinal	302
Ramocha et al (2017), South Africa	Quality of life and physical activity among older adults living in institutions compared to the community	Cross-sectional	80

Rand et al (2015), South Africa	Predicting daily use of the affected upper extremity 1 year after stroke.	Longitudinal	125
Randall et al (2016), African countries	The quality of demographic data on older Africans	Cross-sectional	Not applicable
Rangel et al (2015), South Africa	Mortality after emergency surgery continues to rise after discharge in the elderly: Predictors of 1-year mortality.	Longitudinal (retrospective)	390
Ranjith et al (2017), South Africa	Association Between Hyperuricemia and Major Adverse Cardiac Events in Patients with Acute Myocardial Infarction.	Longitudinal (retrospective)	2683
Rayner et al (2007), South Africa	A survey of hypertensive practices at two community health centres in Cape Town.	Cross-sectional	161
Rayner et al (2012), South Africa	G-protein-coupled receptor kinase 4 polymorphisms predict blood pressure response to dietary modification in Black patients with mild-to-moderate hypertension.	Randomized Control Trials	40
Reddy et al (1985), South Africa	Prevalence and severity of periodontitis in a high fluoride area in South Africa.	Cross-sectional	71
Reiger et al (2017), South Africa	Awareness, treatment, and control of dyslipidaemia in rural South Africa: The HAALSI (Health and Aging in Africa: A Longitudinal Study of an INDEPTH Community in South Africa) study.	Longitudinal	5059
Resnikoff (1988), Chad	Epidemiology of Bietti's keratopathy. Study of risk factors in Central Africa (Chad)	Cross-sectional	3,241
Rishworth et al (2020), Uganda	Getting Old Well in Sub Saharan Africa: Exploring the Social and Structural Drivers of Subjective Wellbeing among Elderly Men and Women in Uganda	Longitudinal	470
Robb et al (2017), South Africa	Malnutrition in the elderly residing in long-term care facilities: a Cross-sectional survey using the Mini Nutritional Assessment (MNA (R)) screening tool	Cross-sectional	124
Rodriguez et al (2002), South Africa	Depression and social support in the elderly population: a study of rural South African elders.	Cross-sectional	Not stated
Rohr et al (2017), South Africa	Performance of self-reported HIV status in determining true HIV status among older adults in rural South Africa: a validation study	Longitudinal	5059
Rosenberg et al (2020) South Africa	The relationships between cognitive function, literacy and HIV status knowledge among older adults in rural South Africa	Longitudinal	5059
Rossouw et al (2017), South Africa	A Comparable Yardstick: Adjusting for Education Bias in South African Health System Responsiveness Ratings	Cross-sectional	1846
Rotchford et al (2000), South Africa	Rapid assessment of cataract surgical coverage in rural Zululand.	Cross-sectional	562
Rotchford et al (2002), South Africa	Glaucoma in Zulus: a population-based Cross-sectional survey in a rural district in South Africa.	Cross-sectional	1115
Rotchford et al (2003), South Africa	Temba glaucoma study: a population-based Cross-sectional survey in urban South Africa.	Cross-sectional	1120

Rotchford et al (2003), South Africa	Exfoliation syndrome in black South Africans.	Cross-sectional	1840
Saeed et al (2016), Ghana	Effect of socio-economic factors in utilization of different healthcare services among older adult men and women in Ghana	Cross-sectional	5573
Sanuade (2019), Ghana	Prevalence and correlates of stroke among older adults in Ghana: Evidence from the Study on Global AGEing and adult health (SAGE).	Cross-sectional	4,279
Sanya et al (2011), Nigeria	Profile and Causes of Mortality Among Elderly Patients Seen in a Tertiary Care Hospital in Nigeria	Longitudinal (cohort)	297
Sarfo et al (2020), Ghana	Risk Factor Control in Stroke Survivors with Diagnosed and Undiagnosed Diabetes: A Ghanaian Registry Analysis	Cross-sectional	101
Sarkodie et al (2020), Ghana	Percutaneous transhepatic biliary stent placement in the palliative management of malignant obstructive jaundice: initial experience in a tertiary center in Ghana	Not stated	23
Schatz et al (2012), South Africa	The impact of pensions on health and wellbeing in rural South Africa: Does gender matter?	Cross-sectional	4085
Schatz et al et al (2015), South Africa	Dependent or Productive? A New Approach to Understanding the Social Positioning of Older South Africans Through Living Arrangements	Cross-sectional	2000 (N= 7518); 2005 (N= 8167); 2010 (N= 10192)
Schatz et al et al (2018), South Africa	Living Arrangements, Disability and Gender of Older Adults Among Rural South Africa.	Longitudinal	5809
Schmidlin et al (2018), South African	Facial ageing in South African adult males	Cross-sectional	189
Schnaid et al (2000), South Africa	Fractured neck of femur in black patients: a prospective study.	Case control	72
Schoeman et al (1996), South Africa	Squamous cell carcinoma in neuropathic plantar ulcers in leprosy: another example of Marjolin's ulcer	Case control series	7
Scholten et al (2011), Uganda	Health and functional status among older people with HIV/AIDS in Uganda	Cross-sectional	510
Segal et al (1980), South Africa	Hiatus hernia in Johannesburg blacks.	Not stated	1092
Segal et al (1982), South Africa	Diverticular disease in urban Africans in South Africa.	Not stated	1048
Segal et al (1988), South Africa	Factors associated with oesophageal cancer in Soweto, South Africa.	Case control	200 - cases, 391 - control
Silbert et al (1977), South Africa	Medical and psychosocial problems of the aged.	Not stated	49347
Simo et al (2020), Cameroon	Correlates of diabetic polyneuropathy of theelderly in Sub-Saharan Africa	Cross-sectional	159
Sissolak et al (2013), South Africa	Tissue microarray in a subset of South African patients with DLBCL.	Longitudinal (retrospective)	93
Skidmore et al (2015), South Africa	Strategy Training During Inpatient Rehabilitation May Prevent Apathy Symptoms After Acute Stroke.	Randomised Controlled Trial	30

Sliwa et al (2010), South Africa	Predisposing factors and incidence of newly diagnosed atrial fibrillation in an urban African community: insights from the Heart of Soweto Study	Longitudinal (cohort)	246
Smith et al (2005), South Africa	Current patient perceptions on the menopause: a South African perspective.	Cross-sectional	541
Solomon et al (1982), South Africa	Osteoarthritis of the hip: the patient behind the disease.	Cross-sectional	105 - advanced OA, 100 - femoral neck acute fracture, 176 normal individuals
Solomon et al (2005), South Africa	The need for tighter rheumatoid arthritis control in a South African public health care center.	Longitudinal (cohort)	359
Solomon et al (2011), South Africa	Burden of depressive symptoms in South African public healthcare patients with established rheumatoid arthritis: a case-control study.	Case-control	643
Solomons (1984), South Africa	Malignant mesothelioma--clinical and epidemiological features. A report of 80 cases.	Longitudinal (retrospective)	80 - study group, 546 - reference group
Somdyala et al (2010), South Africa	Cancer incidence in a rural population of South Africa, 1998-2002.	Not stated	2501
Ssonko et al (2018), Uganda	Polypharmacy among HIV positive older adults on anti-retroviral therapy attending an urban clinic in Uganda	Cross-sectional	412
Surka et al (2001), South Africa	Outcome of high-volume cataract surgery at an academic hospital	Not stated	98
Swart et al (2014), South Africa	Early Loading of Mandibular Implants Placed Immediately After Extraction: A 10-year Prospective Study of Eight Patients	Longitudinal	8
Tannor et al (2017), South Africa	Quality of life in patients on chronic dialysis in South Africa: a comparative mixed methods study.	Cross-sectional	128
Tarekegne et al (2018), Ghana & South Africa	Sociodemographic and behavioral characteristics associated with self-reported diagnosed diabetes mellitus in adults aged 50+ years in Ghana and South Africa: results from the WHO-SAGE wave 1.	Cross-sectional	Ghana 4732, South Africa 3842
Till et al (1999), South Africa	Experience in a hospital-based clinic as part of chiropractic undergraduate training	Cross-sectional	262
Tipping et al (2006), South Africa	The burden and risk factors for adverse drug events in older patients--a prospective Cross-sectional.	Cross-sectional	517
Togonu-Bickersteth (1986), Nigeria	Age Identification Among Yoruba Aged	Not stated	603
Tolani et al (2020), Nigeria	Acute urinary tract infection in patients with underlying benign prostatic hyperplasia and prostate cancer	Cross-sectional	166
Tomás et al (2012), Angola	Predicting perceived health in Angolan elderly: The moderator effect of being oldest old	Cross-sectional	1003
Tomita et al (2013), South Africa	Depression, disability and functional status among community-dwelling older adults in South Africa: evidence from the first South African National Income Dynamics Study	Longitudinal (panel survey)	1429

Torgersen et al (2019), Botswana	Impact of Efavirenz Metabolism on Loss to Care in Older HIV+ Africans	Longitudinal (cohort)	941
Toure et al (2009), Senegal	Risk factors for dementia in a Senegalese elderly population.	Cross-sectional	507
Udjo et al (2006), South Africa	Demographic impact of HIV/AIDS on the young and elderly populations in South Africa.	Longitudinal (cohort)	Not stated
Uwakwe et al (2009), Nigeria	The epidemiology of dependence in older people in Nigeria: prevalence, determinants, informal care, and health service utilization. A 10/66 dementia research group Cross-sectional survey.	Cross-sectional	1238
Uys et al (1990), South Africa	Standards for the nursing care of the frail aged	Not stated	52
Van Biljon et al (2015), South Africa	A partial validation of the WHOQOL-OLD in a sample of older people in South Africa.	Cross-sectional	176
van der Pas et al (2015), South Africa	Features of home and neighbourhood and the liveability of older South Africans	Cross-sectional	1008
van der Weilen et al (2018), Ghana	Universal health coverage in the context of population ageing: What determines health insurance enrolment in rural Ghana?	Longitudinal	5846
van Rensburg et al (2017), South Africa	An elderly, urban population: Their experiences and expectations of pharmaceutical services in community pharmacies	Cross-sectional	67
Van Staden et al (2007), South Africa	Profile of the geriatric patient hospitalised at Universitas Hospital, South Africa.	Longitudinal (retrospective)	791
van Vuuren et al (2009), South Africa	Effectiveness of influenza vaccination in the elderly in South Africa.	Case-control	45522
van Wyk et al (1977), South Africa	Oral health status of institutionalized elderly Cape Coloreds from the Cape Peninsula of South Africa.	Not stated	585
van Wyk et al (1977), South Africa	Tooth survival in institutionalized elderly Cape Coloreds from the Cape Peninsula of South Africa.	Not stated	585
Vlantis et al (2003), South Africa	Conversion from a non-indwelling to a PROVOX 2 indwelling voice prosthesis for speech rehabilitation: comparison of voice quality and patient preference	Longitudinal	17
von Klemperer et al (2014), South Africa	Thrombolysis risk prediction: applying the SITS-SICH and SEDAN scores in South African patients.	Longitudinal	41
Vorobiof et al (2004), South Africa	First line therapy with paclitaxel (Taxol) and pegylated liposomal doxorubicin (Caelyx) in patients with metastatic breast cancer: a multicentre phase II study.	Randomized Control Trials	34
Vorster et al (2015), South Africa	Fluorine-18-fluoroethylcholine PET/CT in the detection of prostate cancer: a South African experience.	Longitudinal (retrospective)	50
Wachira et al (2015), Kenya	Characterization of in-hospital cardiac arrest in adult patients at a tertiary hospital in Kenya	Longitudinal (retrospective)	128

Walker et al (1986), South Africa	Survival of black men with prostatic cancer in Soweto, Johannesburg, South Africa.	Longitudinal (retrospective)	160
Walker et al (1989), South Africa	Risk factors and survival from colorectal cancer in black patients in Soweto, South Africa.	Randomized Control Trials	42
Walker et al (1989), South Africa	Prevalence of gallstones in elderly black women in Soweto, Johannesburg, as assessed by ultrasound.	Not stated	100
Walker et al (1990), South Africa	Obesity in indigent elderly rural African women: effects on hypertension, hyperlipidaemia and hyperglycaemia.	Randomized Control Trials	90
Walker et al (1991), South Africa	Serum albumin levels in elderly rural Africans.	Longitudinal	100
Walker et al (1992), South Africa	Case-control study of prostate cancer in black patients in Soweto, South Africa.	Case control	166
Walker et al (2005), South Africa	Lung cancer in Africans in a South African city population in transition.	Longitudinal (retrospective)	381
Wallrauch et al (2010), South Africa	HIV prevalence and incidence in people 50 years and older in rural South Africa.	Longitudinal	Not reported
Wandera et al (2014), Uganda	Prevalence and correlates of disability among older Ugandans: evidence from the Uganda National Household Survey	Not stated	2628
Wandera et al (2015), Uganda	Factors associated with self-reported ill health among older Ugandans: A Cross-sectional study	Cross-sectional	2382
Wandera, et al (2015), Uganda	Prevalence and risk factors for self-reported non-communicable diseases among older Ugandans: a Cross-sectional	Cross-sectional	2382
Ware et al (2017), South Africa	Associations between dietary salt, potassium and blood pressure in South African adults: WHO SAGE Wave 2 Salt & Tobacco	Longitudinal	2928
Wasserman et al (2007), South Africa	Conservative management of breast cancer in the elderly in a developing country.	Longitudinal (retrospective)	483
Wasserman et al (2009), South Africa	Community-based care of stroke patients in a rural African setting.	Longitudinal	30
Wasserman et al (2012), South Africa	Early outcomes of thrombolysis for acute ischaemic stroke in a South African tertiary care centre.	Longitudinal	42
Waterhouse et al (2017), South Africa	The impact of multi-morbidity on disability among older adults in South Africa: do hypertension and socio-demographic characteristics matter?	Longitudinal	3842
Watkins et al (2014), South Africa	PT321 Economic consequences of cardiovascular disease in South African households: An analysis of the WHO Study on Global Aging (SAGE).	Longitudinal	4895
Webb et al (2015), South Africa	Diabetes care and complications in primary care in the Tshwane district of South Africa.	Randomized Control Trials	599
Wentink et al (2010), South Africa	Incidence and histological features of colorectal cancer in the Northern Cape Province, South Africa.	Longitudinal (retrospective)	206

Werfalli et al (2018), South Africa	Diabetes in South African older adults: prevalence and impact on quality of life and functional disability - as assessed using SAGE Wave 1 data.	Cross-sectional	3836
Wessels et al (2012), South Africa	DVT prophylaxis in relation to patient risk profiling - TUNE-IN study.	Cross-sectional	608
Westaway (2010), South Africa	Effects of ageing, chronic disease and co-morbidity on the health and well-being of older residents of Greater Tshwane.	Cross-sectional	710
Westaway et al (2001), South Africa	The effect of type 2 diabetes mellitus on health-related quality of life (HRQOL).	Longitudinal	487
Westaway et al (2007), South Africa	Which personal quality of life domains affect the happiness of older South Africans?	Longitudinal	854
Westaway et al (2010), South Africa	The impact of chronic diseases on the health and well-being of South Africans in early and later old age	Longitudinal	710
Westaway et al (2015), South Africa	Investigating the psychometric properties of the Rosenberg Self-Esteem scale for South African residents of Greater Pretoria.	Longitudinal	1158
Whigham et al (2011), South Africa	Myocilin mutations in black South Africans with POAG.	Randomized Control Trials	244
Whitelaw et al (1992), South Africa	Community-acquired bacteremia in the elderly: a prospective study of 121 cases.	Longitudinal (retrospective)	121
Whitelaw et al (1994), South Africa	Post-discharge follow-up of stroke patients at Groote Schuur Hospital - a prospective study.	Cross-sectional	70
Whittaker et al (1991), South Africa	Frail aged persons residing in South African homes for the aged who require hospitalisation. Part I. Urban areas.	Longitudinal	9032
Williams et al (2010), South Africa	Major LOXL1 risk allele is reversed in exfoliation glaucoma in a black South African population.	Randomized Control Trials	150
Williams et al (2015), South Africa	MYOC mutations in black south African patients with primary open-angle glaucoma: genetic testing and cascade screening.	Cross-sectional	472
Wilunda (2015), Kenya	Health and ageing in Nairobi's informal settlements-evidence from the International Network for the Demographic Evaluation of Populations and Their Health (INDEPTH): a Cross-sectional study	Cross-sectional	1878
Wolff (1979), South Africa	Health needs of geriatric patients discharged from hospital.	Cross-sectional	500
Wood et al (2007), South Africa	A single unit lymphoma experience: outcome in a Cape Town academic centre.	Longitudinal (retrospective)	253
Yawson (2013), Ghana	Tobacco use in older adults in Ghana: sociodemographic characteristics, health risks and subjective wellbeing	Longitudinal	4305
Yawson (2014), Ghana	Self-reported Cataracts in Older Adults in Ghana: Sociodemographic and Health Related Factors	Longitudinal	4278
Yorston et al (2002), Kenya	Does prospective monitoring improve cataract surgery outcomes in Africa?	Longitudinal	1845

Zengin et al (2017), Gambia	The Gambian Bone and Muscle Ageing Study: Baseline Data from a Prospective Observational African Sub-Saharan Study	Longitudinal	488
Zengin et al (2018), Gambia	The prevalence of sarcopenia and relationships between muscle and bone in ageing West-African Gambian men and women	Longitudinal	488
Zimmer (2005), Sub-Saharan Africa	Older adults in sub-Saharan Africa living with children and grandchildren.	Cross-sectional	50879
Zwi et al (1989), South Africa	Mesothelioma in South Africa, 1976-84: incidence and case characteristics.	Longitudinal (retrospective)	1347
**QUALITATIVE STUDIES (n=71)**			
Aboderin (2004), Ghana	Decline in Material Family Support for Older People in Urban Ghana, Africa: Understanding Processes and Causes of Change.	Interpretive exploratory	51
Adam & Koranteng, (2020), Ghana	Availability, accessibility, and impact of social support on breast cancer treatment among breast cancer patients in Kumasi, Ghana: A qualitative study	Phenomenological	15
Adandom et al (2020), Nigeria	Managing psycho-social-cognitive factors during hip/knee fracture rehabilitation for older adults: clinicians' experiences	Interpretive description	15
Adinkrah (2020), Ghana	Grannicides in Ghana: a study of lethal violence by grandchildren against grandmothers	Exploratory	NA (document analysis)
Agunbiade et al (2012), Nigeria	Ageing, Sexuality and Enhancement Among Yoruba People in South Western Nigeria	Not stated	64 - individual interview, 100 - focus group
Agunbiade et al (2017), Nigeria	Neoliberalism and resilience among older Yoruba people in a semi-urban community, South West Nigeria.	Narrative research	37
Agyemang-Duah et al (2020), Ghana	Dynamics of health information-seeking behaviour among older adults with very low incomes in Ghana: a qualitative study	Not stated	60
Alidu et al (2000), Ghana	'What a dog will see and kill, a cat will see and ignore it': An exploration of health-related help-seeking among older Ghanaian men residing in Ghana and the United Kingdom	Not stated	26
Akinrolie et al (2020), Nigeria	Intergenerational Support between Older Adults and Adult Children in Nigeria: The Role of Reciprocity	Phenomenological	18
Amod et al (2005), South Africa	Ventriculitis due to a hetero strain of vancomycin intermediate Staphylococcus aureus (hVISA): successful treatment with linezolid in combination with intraventricular vancomycin.	Not stated	1
Angotti et al (2018), South Africa	Taking care' in the age of AIDS: older rural South Africans' strategies for surviving the HIV epidemic	Grounded theory	77
Bayuo (2017), Ghana	Experiences with out-patient hospital service utilisation among older persons in the Asante Akyem North District- Ghana	Not stated	16

Bohman et al (2007), South Africa	We clean our houses, prepare for weddings and go to funerals: daily lives of elderly Africans in Majaneng, South Africa.	Ethnography	16
Bohman et al (2009), South Africa	Tradition in transition intergenerational relations with focus on the aged and their family members in a South African context.	Ethnography	29
Bohman et al (2011), South Africa	South Africans' Experiences of Being Old and of Care and Caring in a Transitional Period	Ethnography	16
Bohman et al (2014), South Africa	Existing and Evolving in Two Minds: Beliefs in Relation to Health and Illness Expressed by Older South Africans	Ethnography	16
Brown (2015), Ghana	Elderly caregiving in Ghana: An exploration of family caregivers' perceptions.	Phenomenology	20
Cadmus et al (2012), Nigeria	Older Persons' Views and Experience of Elder Abuse in South Western Nigeria: A Community-Based Qualitative Survey	Grounded theory	64
de Klerk et al (2017), South Africa	“A Body Like a Baby”: Social Self-Care among Older People with Chronic HIV in Mombasa	Ethnography	8
Diaemeta et al (2018), Nigeria	The Burden Experience of Formal and Informal Caregivers of Older Adults With Hip Fracture in Nigeria	Phenomenology	17
Golaz et al (2015), Uganda	Understanding the vulnerability of older adults: Extent of and breaches in support systems in Uganda	Qualitative description	83
Hien et al (2016), Burkina Faso	Point of view of older adults on the potentially inappropriate medications prescribing in primary care facilities in Bobo-Dioulasso, Burkina Faso	Qualitative description	-
Jingi et al (2017), Cameroon	A case management of hypertension in the elderly in sub-Sahara Africa: lessons from Granny.	Qualitative description	1
Kakongi et al (2020), Uganda	Exploring pathways to Hospital Care for Patients with Alzheimer’s disease and related dementias in rural South Western Uganda	Descriptive	24
Kerr et al (2004), South Africa	Factors that influence retirement self-actualisation	Phenomenology	24
Kiplagat et al (2019), Kenya	Challenges with seeking HIV care services: perspectives of older adults infected with HIV in western Kenya	Descriptive qualitative	57
Knight et al (2018), South Africa	"I attend at Vanguard and I attend here as well": barriers to accessing healthcare services among older South Africans with HIV and non-communicable diseases.	Interpretive exploratory	10
Kuteesa et al (2012), Uganda	Older people living with HIV in Uganda: Understanding their experience and needs.	Qualitative description	40
Lekalakala-Mokgele (2014), South Africa	Understanding of the risk of HIV infection among the elderly in Ga-Rankuwa, South Africa.	Not stated	32
Lekalakala-Mokgele (2016), South Africa	Exploring gender perceptions of risk of HIV infection and related behaviour among elderly men and women of Ga-Rankuwa, Gauteng Province, South Africa.	Not stated	32

Leuning et al (2000), South Africa	Meanings and Expressions of Care and Caring for Elders in Urban Namibian Families: A Transcultural Nursing Study	Ethnography	18
Lopes Ibanez-Gonzalez et al (2015)	Clinics and churches: life-worlds and health-seeking practices of older women with non-communicable disease in rural South Africa.	Qualitative description	13
Matovu & Wallhagen (2020), Uganda	Perceived Caregiver Stress, Coping, and Quality of Life of Older Ugandan Grandparent-Caregivers	Grounded theory	32
Matovu et al (2020), Uganda	Ugandan Jajjas: Antecedents and rewards of caring for grandchildren in the context of HIV	Grounded theory	32
Mkhonto et al (2018), South Africa	When people with dementia are perceived as witches. Consequences for patients and nurse education in South Africa	Qualitative description	37
Muchiri et al (2012), South Africa	Needs and preferences for nutrition education of type 2 diabetic adults in a resource-limited setting in South Africa	Phenomenology	31
Mushi et al (2014), Tanzania	Social Representation and Practices Related to Dementia in Hai District of Tanzania	Phenomenology	41
Nadasen (2008), South Africa	"Life Without Line Dancing and the Other Activities Would Be Too Dreadful to Imagine": An Increase in Social Activity for Older Women	Not stated	31
Naido et al (2019), South Africa	What the Elderly Experience and Expect From Primary Care Services in KwaZulu-Natal, South Africa	Interpretative exploratory	28
Nwankwo et al (2019), Nigeria	The clinical experiences of Nigerian physiotherapists in managing environmental and socioeconomic determinants of mobility for older adults	Qualitative description	20
Obi et al (2019), Nigeria	The Experience and Perception of Physiotherapists in Nigeria re: Fall Prevention in Recurrent-Faller Older Adults	Qualitative description	12
Ojembe et al (2018), Nigeria	Describing reasons for loneliness among older people in Nigeria	phenomenology	12
Ojembe et al (2019), Nigeria	Television, radio, and telephone: Tools for reducing loneliness among older adults in Nigeria	Phenomenology	15
Okoh et al (2020), Nigeria	Nigerian healthcare workers’ perception of transdisciplinary approach to older adults’ care: A qualitative case study	Case study	16
Rajak et al (2009), Ghana	Further insight into West African crystalline maculopathy.	Case studies	14
Richards et al (2013), Uganda	Neglected older women and men: Exploring age and gender as structural drivers of HIV among people aged over 60 in Uganda	Qualitative description	42
Roos et al (2010), South Africa	Older persons' experiences of loneliness: A South African perspective.	Not stated	31
Roos et al (2012), South Africa	The role of context and the interpersonal experience of loneliness among older people in a residential care facility.	Qualitative description	10
Roos et al (2014), South Africa	(Re)creating community: Experiences of older women forcibly relocated during apartheid.	Ethnography	11

Roos et al (2016), South Africa	Older people's experiences of giving and receiving empathy in relation to middle adolescents in rural South Africa.	Not stated	8
Roos et al (2017), South Africa	Intergenerational Care Perceptions of Older Women and Middle Adolescents in a Resource-Constrained Community in South Africa.	Narrative	10
Rotchford et al (2002), South Africa	Reasons for poor cataract surgery uptake - a qualitative study in rural South Africa.	Phenomenology	20
Rutagumirwa et al (2019), Tanzania	“I Have to Listen to This Old Body”: Femininity and the Aging Body	Grounded theory	60
Schatz et al (2009), South Africa	Reframing vulnerability: Mozambican refugees' access to state-funded pensions in rural South Africa.	Narrative	30
Schatz et al (2012), South Africa	My heart is very painful: Physical, mental and social wellbeing of older women at the times of HIV/AIDS in rural South Africa	Narrative	30
Schatz et al (2014), South Africa	My legs affect me a lot. ... I can no longer walk to the forest to fetch firewood: challenges related to health and the performance of daily tasks for older women in a high HIV context.	Narrative	30
Schatz et al (2018), South Africa	"I Was Referred From the Other Side": Gender and HIV Testing Among Older South Africans Living With HIV	Grounded theory	21
Schatz et al (2019), Uganda	For us here, we remind ourselves: strategies and barriers to ART access and adherence among older Ugandans.	Not stated	40
Sidloyi et al (2016), South Africa	Survival strategies of elderly women in Ngangelizwe Township, Mthatha, South Africa: Livelihoods, social networks and income.	Not stated	15
Singo et al (2015), South Africa	The views of the elderly on the impact that HIV and AIDS has on their lives in the Thulamela Municipality, Vhembe District, Limpopo province.	Qualitative description	12
Skovdal et al (2011), Zimbabwe	Challenges faced by elderly guardians in sustaining the adherence to antiretroviral therapy in HIV-infected children in Zimbabwe.	Not stated	33
Sobnach et al (2009), South Africa	First case report of pharyngeal cysticercosis	Case study	1
Ssengonzi (2007), Uganda	The Plight of Older Persons as Caregivers to People Infected/Affected by HIV/AIDS: Evidence from Uganda	Not stated	10
Tanyi, et al (2018), Cameroon	HIV/AIDS and older adults in Cameroon: Emerging issues and implications for caregiving and policy-making	Qualitative descriptive analysis	36
Udvardy et al (1992), South Africa	Gender, aging and power in sub-Saharan Africa: Challenges and puzzles.	Ethnography	7
van Biljon et al (2015), South Africa	A Conceptual Model of Quality of Life for Older People in Residential Care Facilities in South Africa	Grounded theory	19
Van der Geest (2002), Ghana	I want to go!' How older people in Ghana look forward to death	Ethnography	35
Van der Geest et al (2004), Ghana	“They don’t come to listen”: The experience of loneliness among older people in Kwahu, Ghana	Ethnography	27

Van Dongen (2003), South Africa	Die lewe vat ek net soos ek dit kry. Life stories and remembrance of older coloured people on farms in the western cape province.	Ethnography	10
Van Dongen (2005), South Africa	Remembering in times of misery: Can older people in South Africa 'get through'?	Ethnography	50
Wilkinson et al (2013), South Africa	Family members' perceptions and expectations of the use of syringe drivers: a South African study.	Qualitative description	8
**MIXED METHOD (n=15)**			
Afolabi et al (2019) Nigeria	Gender analysis of nurses' attitude towards care of the elderly with dementia in Obafemi Awolowo University Teaching Hospitals Complex, Ile-Ife, Osun State, Nigeria	Sequential exploratory	100
Deist et al (2017), South Africa	Living with a parent with dementia: A family resilience study	Cross-sectional	47
Drah (2014), Ghana & South Africa	'Older Women', Customary Obligations and Orphan Foster Caregiving: The Case of Queen Mothers in Manya Klo, Ghana	Not stated	34
Frost (2015), Sub-Saharan Africa	Care of the Elderly: Survey of Teaching in an Aging Sub-Saharan Africa	Cross-sectional	124
Geyer (2010), South Africa	Strengths-based group work with alcohol dependent older persons: Solution to an age-old problem?	Concurrent triangulation	8
Kuteesa et al (2014), Uganda	Experiences of HIV-related stigma among HIV-positive older persons in Uganda-A mixed methods analysis.	Informative	183
Naah et al (2020), Cameroon	Determinants of Active and Healthy Ageing in Sub-Saharan Africa: Evidence from Cameroon	Not stated	100
Ndou et al (2013), South Africa	A rapid assessment of a community health worker pilot programme to improve the management of hypertension and diabetes in Emfuleni sub-district of Gauteng Province, South Africa.	Retrospective case study approach	56
Peltzer (2004), South Africa	Health beliefs and prescription medication compliance among diagnosed hypertension clinic attenders in a rural South African hospital	Not stated	100
Phillips-Howard et al (2014), Kenya	Deaths Ascribed to Non-Communicable Diseases among Rural Kenyan Adults Are Proportionately Increasing: Evidence from a Health and Demographic Surveillance System, 2003-2010	Not stated	15228
Pienaar et al (2010), South Africa	Self-reported outcomes of aural rehabilitation for adult hearing aid users in a South African context.	Not stated	61
Rhoda et al (2015), South Africa	Provision of inpatient rehabilitation and challenges experienced with participation post discharge: quantitative and qualitative inquiry of African stroke patients.	Retrospective record review	452
Schatz (2007), South Africa	'Taking care of my own blood': older women's relationships to their households in rural South Africa	Convergent	qualitative - 24, quantitative - 70272

Semeere et al (2014), Uganda	Mortality and Immunological Recovery Among Older Adults on Antiretroviral Therapy at a Large Urban HIV Clinic in Kampala, Uganda	Not stated	9,806
Watson et al (2013), South Africa	Community-based collaboration in support of older persons.	Convergent	333

Appendix 3S

Reference 2 (included articles, n = 512)

Abbai, N. S., Nyirenda, M., Reddy, T., & Ramjee, G. (2017). Good correlation between the Afinion AS100 analyser and the ABX Pentra 400 analyser for the measurement of glycosylated haemoglobin and lipid levels in older adults in Durban, South Africa. *South African Medical Journal = Suid-Afrikaanse Tydskrif Vir Geneeskunde*, *108*(1), 50–55. https://doi.org/10.7196/SAMJ.2017.v108i1.12548

Abelson, M. (2013). Left atrial appendage closure in patients with atrial fibrillation in whom warfarin is contra-indicated: Initial South African experience. *Cardiovascular Journal of Africa*, *24*(4), 107–109. https://doi.org/10.5830/CVJA-2013-018

Abene, E. E., Gimba, Z. M., Edah, J. O., Akinbuwa, B. A., Uchendu, D. G., Onyenuche, C., ... & Agaba, E. I. (2020). Blood pressure control and kidney damage in hypertension: Results of a three-center cross-sectional study in North Central Nigeria. *Nigerian Journal of Clinical Practice*, *23*(11), 1590.

Aboderin, I. (2004). Decline in Material Family Support for Older People in Urban Ghana, Africa: Understanding Processes and Causes of Change. In *Journals of Gerontology - Series B Psychological Sciences and Social Sciences* (Vol. 59, Issue 3, pp. S128–S137). Gerontological Society of America. https://doi.org/10.1093/geronb/59.3.S128

Aboderin, I., & Nanyonjo, A. (2017). Musculoskeletal health conditions among older populations in urban slums in sub-Saharan Africa. In *Best Practice and Research: Clinical Rheumatology* (Vol. 31, Issue 2, pp. 115–128). Bailliere Tindall Ltd. https://doi.org/10.1016/j.berh.2017.11.001

Ackuaku-Dogbe, E. M., Yawson, A. E., & Biritwum, R. B. (2015). Cataract Surgical Uptake Among Older Adults in Ghana. *Ghana Medical Journal*, *49*(2), 84–89. https://doi.org/10.4314/gmj.v49i2.4

Adam, S. G. L. M. D. (2013). Effect of pre-fracture mobility on the early post-operative functional outcome in elderly patients with a hip fracture. *South African Journal of Physiotherapy*, *69*(3), 13–19.

Adam, A., & Koranteng, F. (2020). Availability, accessibility, and impact of social support on breast cancer treatment among breast cancer patients in Kumasi, Ghana: A qualitative study. *PloS one*, *15*(4), e0231691.

Adandom, I. I., Jumbo, S. U., Diameta, E., Nwankwo, H. C., Akinola, B., & Kalu, M. E. (2020). Managing Psycho-Social-Cognitive Factors during Hip/knee

Fracture Rehabilitation for Older Adults: Clinicians' Experiences. *Nigerian Hospital Practice*, *26*(1-2), 23-32.

Adebajo, A. O., Cooper, C., & Evans, J. G. (1991). Fractures of the hip and distal forearm in west africa and the United Kingdom. *Age and Ageing*, *20*(6), 435–438. https://doi.org/10.1093/ageing/20.6.435

Adebusoye, L., Ogunbode, A., Olowookere, O., Ajayi, S., & Ladipo, M. (2018). Factors associated with sarcopenia among older patients attending a geriatric clinic in Nigeria. *Nigerian Journal of Clinical Practice*, *21*(4), 443–450. https://doi.org/10.4103/njcp.njcp_374_17

Adebusoye, L. A., Olowookere, O. O., Ajayi, S. A., Akinmoladun, V. I., & Alonge, T. O. (2020). Mortality Trends among Older Patients Admitted to the Geriatric Centre, University College Hospital, Ibadan, Nigeria, 2013-2017. *West African Journal of Medicine*, *37*(3), 209-215.

Adhvaryu, A. R., & Beegle, K. (2012). The long-run impacts of adult deaths on older household members in Tanzania. *Economic Development and Cultural Change*, *60*(2), 245–277. https://doi.org/10.1086/662577

Adinkrah, M. (2020). Grannicides in Ghana: a study of lethal violence by grandchildren against grandmothers. *Journal of elder abuse & neglect*, *32*(3), 275-294.

Agboghoroma, O. F., Akemokwe, F. M., & Puepet, F. H. (2020). Peripheral arterial disease and its correlates in patients with type 2 diabetes mellitus in a teaching hospital in northern Nigeria: a cross-sectional study. *BMC cardiovascular disorders*, *20*(1), 1-6.

Agbozo, F., Amardi-Mfoafo, J., Dwase, H., & Ellahi, B. (2018). Nutrition knowledge, dietary patterns and anthropometric indices of older persons in four peri-urban communities in Ga West municipality, ghana. *African Health Sciences*, *18*(3), 743–755. https://doi.org/10.4314/ahs.v18i3.33

Agunbiade, O. M., & Akinyemi, A. I. (2016). Neoliberalism and resilience among older yoruba people in a semiurban community, South West Nigeria. In *Cross-Cultural and Cross-Disciplinary Perspectives in Social Gerontology* (pp. 85–107). Springer Singapore. https://doi.org/10.1007/978-981-10-1654-7_5

Agunbiade, O. M., & Ayotunde, T. (2012). Ageing, sexuality and enhancement among Yoruba people in south western Nigeria. *Culture, Health and Sexuality*, *14*(6), 705–717. https://doi.org/10.1080/13691058.2012.677861

Agyemang-Duah, W., Peprah, C., & Arthur-Holmes, F. (2020). Predictors of healthcare utilisation among poor older people under the livelihood empowerment against poverty programme in the Atwima Nwabiagya District of Ghana. *BMC geriatrics*, *20*(1), 1-11.

Agyemang-Duah, W., Arthur-Holmes, F., Peprah, C., Adei, D., & Peprah, P. (2020). Dynamics of health information-seeking behaviour among older adults with very low incomes in Ghana: a qualitative study. *BMC Public Health*, *20*(1), 1-13.

Aheto, J. M. K., Udofia, E. A., Kallson, E., Mensah, G., Nadia, M., Chatterji, S., ... & Yawson, A. E. (2020). Prevalence, socio-demographic and environmental determinants of asthma in 4621 Ghanaian adults: Evidence from Wave 2 of the World Health Organization's study on global AGEing and adult health. *PLoS One*, *15*(12), e0243642.

Akande-Sholabi, W., Agha, P. C., Olowookere, O. O., & Adebusoye, L. A. (2020). Evaluation of prescription pattern of analgesic use among ambulatory elderly in South-Western Nigeria. *Annals of African Medicine*, *19*(2), 131.

Akande-Sholabi, W., Ogundipe, F. S., & Adisa, R. (2020). Pharmacists' knowledge and counselling on fall risk increasing drugs in a tertiary teaching hospital in Nigeria. *BMC health services research*, *20*, 1-9.

Akinrolie, O., Okoh, A. C., & Kalu, M. E. (2020). Intergenerational support between older adults and adult children in Nigeria: The role of reciprocity. *Journal of Gerontological Social Work*, *63*(5), 478-498.

Akinyemi, A., Bamiwuye, O., Inathaniel, T., Ijadunola, K., & Fatusi, A. (2008). The Nigerian Aging Males' Symptoms scale. Experience in elderly males. *Aging Male*, *11*(2), 89–93. https://doi.org/10.1080/13685530802169871

Akinyemi, J. O., Ogunbosi, B. O., Fayemiwo, A. S., Adesina, O. A., Obaro, M., Kuti, M. A., Awolude, O. A., Olaleye, D. O., & Adewole, I. F. (2017). Demographic and epidemiological characteristics of HIV opportunistic infections among older adults in Nigeria. *African Health Sciences*, *17*(2), 315–321. https://doi.org/10.4314/ahs.v17i2.4

Akinyemi, R. O., Allan, L., Owolabi, M. O., Akinyemi, J. O., Ogbole, G., Ajani, A., Firbank, M., Ogunniyi, A., & Kalaria, R. N. (2014). Profile and determinants of vascular cognitive impairment in African stroke survivors: The CogFAST Nigeria Study. *Journal of the Neurological Sciences*, *346*(1–2), 241–249. https://doi.org/10.1016/j.jns.2014.08.042

Akinyemi, R. O., Firbank, M., Ogbole, G. I., Allan, L. M., Owolabi, M. O., Akinyemi, J. O., Yusuf, B. P., Ogunseyinde, O., Ogunniyi, A., & Kalaria, R. N. (2015). Medial temporal lobe atrophy, white matter hyperintensities and cognitive impairment among Nigerian African stroke survivors. *BMC Research Notes*, *8*(1), 625. https://doi.org/10.1186/s13104-015-1552-7

Akinyemi, R. O., Izzeldin, I. M. H., Dotchin, C., Gray, W. K., Adeniji, O., Seidi, O. A., Mwakisambwe, J. J., Mhina, C. J., Mutesi, F., Msechu, H. Z., Mteta, K.

A., Ahmed, M. A. M., Hamid, S. H. M., Abuelgasim, N. A. A., Mohamed, S. A. A., Mohamed, A. Y. O., Adesina, F., Hamzat, M., Olunuga, T., … Walker, R. (2014). Contribution of noncommunicable diseases to medical admissions of elderly adults in Africa: A prospective, Cross-Sectional study in Nigeria, Sudan, and Tanzania. *Journal of the American Geriatrics Society*, *62*(8), 1460–1466. https://doi.org/10.1111/jgs.12940

Akor, A. A., Bamidele, A., & Erhabor, G. E. (2020). Predictors of Health-Related Quality of Life (HRQOL) in Patients With Chronic Obstructive Pulmonary Disease using the COPD Assessment Test (CAT). *West African Journal of Medicine*, *37*(3), 275-280.

Akoria, O., Osian, F., Akene, B., Ugorji, E., & Emore, O. (2021). Evaluation of Documentation of Admissions into A Geriatrics Unit in Nigeria: 2014-2018. *West African Journal of Medicine*, *38*(1), 873-879.

Akosile, Christopher O., Anukam, G. O., Johnson, O. E., Fabunmi, A. A., Okoye, E. C., Iheukwumere, N., & Akinwola, M. O. (2014). Fear of Falling and Quality of Life of Apparently-Healthy Elderly Individuals from a Nigerian Population. *Journal of Cross-Cultural Gerontology*, *29*(2), 201–209. https://doi.org/10.1007/s10823-014-9228-7

Akosile, Christopher Olusanjo, Mgbeojedo, U. G., Maruf, F. A., Okoye, E. C., Umeonwuka, I. C., & Ogunniyi, A. (2018). Depression, functional disability and quality of life among Nigerian older adults: Prevalences and relationships. *Archives of Gerontology and Geriatrics*, *74*, 39–43. https://doi.org/10.1016/j.archger.2017.08.011

Akuamoah-Boateng, H. (2013). Self-reported vision health status among older people in the Kassena-Nankana District, Ghana. *Global Health Action*, *6*(1), 19012. https://doi.org/10.3402/gha.v6i0.19012

Alberts, A. S., Falkson, G., & Van Der Merwe, R. (1991). *Metastatic breast cancer - age has a significant effect on survival*. South African Medical Journal. https://www.ajol.info/index.php/samj/article/view/158041

Alidu, L., & Grunfeld, E. A. (2020). ‘What a dog will see and kill, a cat will see and ignore it’: An exploration of health-related help-seeking among older Ghanaian men residing in Ghana and the United Kingdom. *British Journal of Health Psychology*, *25*(4), 1102-1117.

Allain, T. J., Mwambelo, M., Mdolo, T., & Mfune, P. (2014). Falls and other geriatric syndromes in Blantyre, Malawi: A community survey of older adults. *Malawi Medical Journal*, *26*(4), 105–108. www.mmj.medcol.mw

Amegbor, P. M., Kuuire, V. Z., Robertson, H., & Kuffuor, O. A. (2018). Predictors of basic self-care and intermediate self-care functional disabilities among older adults in Ghana. *Archives of Gerontology and Geriatrics*, *77*, 81–88. https://doi.org/10.1016/j.archger.2018.04.006

Amegbor, P. M., Braimah, J. A., Adjaye-Gbewonyo, D., Rosenberg, M. W., & Sabel, C. E. (2020). Effect of cognitive and structural social capital on depression among older adults in Ghana: A multilevel cross-sectional analysis. *Archives of gerontology and geriatrics*, *89*, 104045.

Ameh, S., Gómez-Olivé, F. X., Kahn, K., Tollman, S. M., & Klipstein-Grobusch, K. (2014). Predictors of health care use by adults 50 years and over in a rural South African setting. *Global Health Action*, *7*(1). https://doi.org/10.3402/gha.v7.24771

Amod, F., Moodley, I., Peer, A. K. C., Sunderland, J., Lovering, A., Wootton, M., Nadvi, S., & Vawda, F. (2005). Ventriculitis due to a hetero strain of vancomycin intermediate Staphylococcus aureus (hVISA): Successful treatment with linezolid in combination with intraventricular vancomycin. *Journal of Infection*, *50*(3), 252–257. https://doi.org/10.1016/j.jinf.2004.04.002

Amoo, G., Ogundele, A. T., Olajide, A. O., Ighoroje, M. G., Oluwaranti, A. O., Onunka, G. C., ... & Folaji, O. G. (2020). Prevalence and pattern of psychiatric morbidity among community-dwelling elderly populations in Abeokuta, Nigeria. *Journal of geriatric psychiatry and neurology*, *33*(6), 353-362.

Amosun, S. L. (2014). The process of enhancing a geriatric module in undergraduate physiotherapy education in South Africa--perceived attitudes towards ageing among community-dwelling elderly persons in cape town. *South African Journal of Physiotherapy*, *70*(1), 24–30. https://go.gale.com/ps/i.do?p=AONE&sw=w&issn=03796175&v=2.1&it=r&id=GALE%7CA455784091&sid=googleScholar&linkaccess=fulltext

Amosun, S. L., Burgess, T., Groeneveldt, L., & Hodgson, T. (2007). Are elderly pedestrians allowed enough time at pedestrian crossings in Cape Town, South Africa? *Physiotherapy Theory and Practice*, *23*(6), 325–332. https://doi.org/10.1080/09593980701593755

Annin, K., Saeed, B., Yawson, A., Musah, A. A., Nakua, E., Agyei-Baffour, P., & Nsowah-Nuamah, N. N. (2014). Assessing the association between the degree of pain and socioeconomic status among older persons in Ghana. *Global Journal of Health Science*, *6*(3), 155–164. https://doi.org/10.5539/gjhs.v6n3p155

Ardington, C., Case, A., Islam, M., Lam, D., Leibbrandt, M., Menendez, A., & Olgiati, A. (2010). The impact of AIDS on intergenerational support in South Africa: Evidence from the cape area panel study. *Research on Aging*, *32*(1), 97–121. https://doi.org/10.1177/0164027509348143

Awoke, M. A., Negin, J., Moller, J., Farell, P., Yawson, A. E., Biritwum, R. B., & Kowal, P. (2017). Predictors of public and private healthcare utilization and associated health system responsiveness among older adults in Ghana. *Global Health Action*, *10*(1). https://doi.org/10.1080/16549716.2017.1301723

Awuviry-Newton, K., Ofori-Dua, K., & Newton, A. (2020). Correlates of older adult inpatients' personal care provision to people with functional difficulties in Ghana. *PloS one*, *15*(10), e0238693.

Awuviry-Newton, K., Wales, K., Tavener, M., & Byles, J. (2020). Do factors across the World Health Organisation's International Classification of Functioning, Disability and Health framework relate to caregiver availability for community-dwelling older adults in Ghana?. *PloS one*, *15*(5), e0233541.

Ayernor, P. K. (2012). Diseases of ageing in Ghana. *Ghana Medical Journal*, *46*(2 Suppl), 18–22. /pmc/articles/PMC3645144/?report=abstract

Ayodapo, A. O., Elegbede, O. T., Omosanya, O. E., & Monsudi, K. F. (2020). Patient education and medication adherence among hypertensives in a tertiary hospital, South Western Nigeria. *Ethiopian journal of health sciences*, *30*(2).

Ayokunle, A. M., Oyeyemi, F. T., Onipede, W., O, T. F., Olagunju, A. E., Makinde, G. B., Olawole-Isaac, A., & Oluwatomipe, A. P. (2015). The Definitions and Onset of an Old Person in South-Western Nigeria. *Educational Gerontology*, *41*(7), 494–503. https://doi.org/10.1080/03601277.2014.1003492

Ayuk, A. E., Omoronyia, O. E., Asibong, U. E., Enang, O. E., Legogie, A. O., & Nwafor, K. N. (2020). Impact of diabetes mellitus on sexuality in a developing country setting: A case-control study in Calabar, Nigeria. *Nigerian Journal of Clinical Practice*, *23*(6), 870.

Balogun, S., Yusuff, H., Adeleye, B., Balogun, M., Aminu, A., Yusuf, K., & Tettey, P. (2018). Determinants of bed net use among older people in Nigeria: Results from a nationally representative survey. *Pan African Medical Journal*, *31*. https://doi.org/10.11604/pamj.2018.31.112.12627

Bastawrous, A., Mathenge, W., Wing, K., Rono, H., Gichangi, M., Weiss, H. A., Macleod, D., Foster, A., Burton, M. J., & Kuper, H. (2016). Six-year incidence of blindness and visual impairment in Kenya: The Nakuru eye disease cohort study. *Investigative Ophthalmology and Visual Science*, *57*(14), 5974–5983. https://doi.org/10.1167/iovs.16-19835

Beaugé, Y., De Allegri, M., Ouédraogo, S., Bonnet, E., Kuunibe, N., & Ridde, V. (2020). Do Targeted User Fee Exemptions Reach the Ultra-Poor and Increase their Healthcare Utilisation? A Panel Study from Burkina Faso. *International Journal of Environmental Research and Public Health*, *17*(18), 6543.

Bennett, R., Chepngeno-Langat, G., Evandrou, M., & Falkingham, J. (2016). Gender differentials and old age survival in the Nairobi slums, Kenya. *Social Science and Medicine*, *163*, 107–116. https://doi.org/10.1016/j.socscimed.2016.07.002

Biritwum, R. B., Mensah, G., Minicuci, N., Yawson, A. E., Naidoo, N., Chatterji, S., & Kowal, P. (2013). Household characteristics for older adults and study background from SAGE Ghana Wave 1. *Global Health Action*, *6*(1). https://doi.org/10.3402/gha.v6i0.20096

Bloomfield, G. S., DeLong, A. K., Akwanalo, C. O., Hogan, J. W., Carter, E. J., Aswa, D. F., Binanay, C., Koech, M., Kimaiyo, S., & Velazquez, E. J. (2016). Markers of Atherosclerosis, Clinical Characteristics, and Treatment Patterns in Heart Failure: A Case-Control Study of Middle-Aged Adult Heart Failure

Patients in Rural Kenya. *Global Heart*, *11*(1), 97. https://doi.org/10.1016/j.gheart.2015.12.014

Boateng, G. O., Adams, E. A., Odei Boateng, M., Luginaah, I. N., & Taabazuing, M.-M. (2017). Obesity and the burden of health risks among the elderly in Ghana: A population study. *PLOS ONE*, *12*(11), e0186947. https://doi.org/10.1371/journal.pone.0186947

Bohman, D. M., Van Wyk, N. C., & Ekman, S. (2014). Existing and evolving in two minds: Beliefs in relation to health and illness expressed by older South Africans. *Africa Journal of Nursing and Midwifery*, *16*(2), 139–152. https://doi.org/10.25159/2520-5293/37

Bohman, Doris M., Vasuthevan, S., Van Wyk, N. C., & Ekman, S. L. (2007). “We clean our houses, prepare for weddings and go to funerals”: Daily lives of elderly Africans in Majaneng, South Africa. *Journal of Cross-Cultural Gerontology*, *22*(4), 323–337. https://doi.org/10.1007/s10823-007-9040-8

Boon, H., James, S., Ruiter, R. A. C., Van Den Borne, B., Williams, E., & Reddy, P. (2010). Explaining perceived ability among older people to provide care as a result of HIV and AIDS in South Africa. *AIDS Care - Psychological and Socio-Medical Aspects of AIDS/HIV*, *22*(4), 399–408. https://doi.org/10.1080/09540120903202921

Boon, H., Ruiter, R. A. C., James, S., Van Den Borne, B., Williams, E., & Reddy, P. (2009). The impact of a community-based pilot health education intervention for older people as caregivers of orphaned and sick children as a result of HIV and AIDS in South Africa. *Journal of Cross-Cultural Gerontology*, *24*(4), 373–389. https://doi.org/10.1007/s10823-009-9101-2

Boon, H., Ruiter, R. A. C., James, S., Van Den Borne, B., Williams, E., & Reddy, P. (2010). Correlates of grief among older adults caring for children and grandchildren as a consequence of HIV and AIDS in South Africa. *Journal of Aging and Health*, *22*(1), 48–67. https://doi.org/10.1177/0898264309349165

Brathwaite, D., Mogotlane, S., Rodriguez, H., Dorsey, S., Mangongo, R., & Matlakala, M. (2002). Elderly citizen’s perception of their health and care provided in a rural South African community. *ABNF Journal*, *13*(2), 37–41. https://go.gale.com/ps/i.do?p=AONE&sw=w&issn=10467041&v=2.1&it=r&id=GALE%7CA93610977&sid=googleScholar&linkaccess=fulltext

Cadmus, E. O., Owoaje, E. T., & Akinyemi, O. O. (2015). Older persons’ Views and experience of elder abuse in south western Nigeria: A community-based qualitative survey. *Journal of Aging and Health*, *27*(4), 711–729. https://doi.org/10.1177/0898264314559893

Callixte, K. T., Clet, T. B., Jacques, D., Faustin, Y., François, D. J., & Maturin, T. T. (2015). The pattern of neurological diseases in elderly people in outpatient

consultations in Sub-Saharan Africa. *BMC Research Notes*, *8*(1). https://doi.org/10.1186/s13104-015-1116-x

Calys-Tagoe, B., Nuertey, B. D., Tetteh, J., & Yawson, A. E. (2020). Individual awareness and treatment effectiveness of hypertension among older adults in Ghana: evidence from the World Health Organization study of global ageing and adult health wave 2. *The Pan African Medical Journal*, *37*.

Calys-Tagoe, B. N. L., Hewlett, S. A., Dako-Gyeke, P., Yawson, A. E., Baddoo, N. A., Seneadza, N. A. H., Mensah, G., Minicuci, N., Naidoo, N., Chatterji, S., Kowal, P., & Biritwum, R. B. (2014). Predictors of subjective well-being among older Ghanaians. *Ghana Medical Journal*, *48*(4), 178–184. https://doi.org/10.4314/gmj.v48i4.2

Charlton, K. E., Kolbe-Alexander, T. L., & Nel, J. H. (2007). The MNA, but not the DETERMINE, screening tool is a valid indicator of nutritional status in elderly Africans. *Nutrition*, *23*(7–8), 533–542. https://doi.org/10.1016/j.nut.2007.04.015

Charlton, K. E., Levitt, N. S., & Lombard, C. J. (1997). *The prevalence of diabetes mellitus and associated risk factors in elderly coloured South Africans*. South African Medical Journal. https://pubmed.ncbi.nlm.nih.gov/9137356/

Chepngeno-Langat, G. (2013). Perception of vulnerability to HIV infection among older people in Nairobi, Kenya: A need for intervention. *Journal of Biosocial Science*, *45*(2), 249–266. https://doi.org/10.1017/S0021932012000417

Chepngeno-Langat, G. (2014). Entry and re-entry into informal care-giving over a 3-year prospective study among older people in Nairobi slums, Kenya. *Health and Social Care in the Community*, *22*(5), 533–544. https://doi.org/10.1111/hsc.12114

Chepngeno-Langat, G., Falkingham, J. C., Madise, N. J., & Evandrou, M. (2012). Concern About HIV and AIDS Among Older People in the Slums of Nairobi, Kenya. *Risk Analysis*, *32*(9), 1512–1523. https://doi.org/10.1111/j.1539-6924.2011.01765.x

Chepngeno-Langat, G., Madise, N., Evandrou, M., & Falkingham, J. (2011). Gender differentials on the health consequences of care-giving to people with AIDS-related illness among older informal carers in two slums in Nairobi, Kenya. *AIDS Care*, *23*(12), 1586–1594. https://doi.org/10.1080/09540121.2011.569698

Chilima, D. M., & Ismail, S. J. (1998). Anthropometric characteristics of older people in rural Malawi. *European Journal of Clinical Nutrition*, *52*(9), 643–649. https://doi.org/10.1038/sj.ejcn.1600617

Chilima, Dorothy M, & Ismail, S. J. (2001). Nutrition and handgrip strength of older adults in rural Malawi. *Public Health Nutrition*, *4*(1), 11–17.

https://doi.org/10.1079/phn200050

Chukwuorji, J. B. C., Nwoke, M. B., & Ebere, M. O. (2017). Stressful life events, family support and successful ageing in the Biafran War generation. *Aging and Mental Health*, *21*(1), 95–103. https://doi.org/10.1080/13607863.2015.1083946

Clark, D. O., Gao, S., Lane, K. A., Callahan, C. M., Baiyewu, O., Ogunniyi, A., & Hendrie, H. C. (2014). Obesity and 10-year mortality in very old african americans and yoruba-nigerians: Exploring the obesity paradox. *Journals of Gerontology - Series A Biological Sciences and Medical Sciences*, *69*(9), 1162–1169. https://doi.org/10.1093/gerona/glu035

Clausen, T., Charlton, K. E., Gobotswang, K. S. M., & Holmboe-Ottesen, G. (2005). Predictors of food variety and dietary diversity among older persons in Botswana. *Nutrition*, *21*(1), 86–95. https://doi.org/10.1016/j.nut.2004.09.012

Dake, F. A., & Van Der Wielen, N. (2020). Towards universal access to healthcare for older adults: an assessment of the old-age exemption policy under Ghana's National Health Insurance Scheme. *International journal for equity in health*, *19*(1), 1-10.

De Jager, C. A., Msemburi, W., Pepper, K., & Combrinck, M. I. (2017). Dementia Prevalence in a Rural Region of South Africa: A Cross-Sectional Community Study. *Journal of Alzheimer's Disease*, *60*(3), 1087–1096. https://doi.org/10.3233/JAD-170325

de Klerk, J., & Moyer, E. (2017). "A Body Like a Baby": Social Self-Care among Older People with Chronic HIV in Mombasa. *Medical Anthropology*, *36*(4), 305–318. https://doi.org/10.1080/01459740.2016.1235573

De Picciotto, J., & Friedland, D. (2001). Verbal fluency in elderly bilingual speakers: Normative data and preliminary application to Alzheimer's disease. *Folia Phoniatrica et Logopaedica*, *53*(3), 145–152. https://doi.org/10.1159/000052669

de Terline, D. M., Kramoh, K. E., Diop, I. B., Nhavoto, C., Balde, D. M., Ferreira, B., ... & Antignac, M. (2020). Poor adherence to medication and salt restriction as a barrier to reaching blood pressure control in patients with hypertension: Cross-sectional study from 12 sub-Saharan countries. *Archives of Cardiovascular Diseases*, *113*(6-7), 433-442.

de Villiers, L., Badri, M., Ferreira, M., & Bryer, A. (2011). Stroke outcomes in a socio-economically disadvantaged urban community. *South African Medical Journal*, *101*(5), 345–348. https://doi.org/10.7196/samj.4588

De Villiers, P. J. T., Steele, A. D., Hiemstra, L. A., Rappaport, R., Dunning, A. J., Gruber, W. C., & Forrest, B. D. (2009). Efficacy and safety of a live

attenuated influenza vaccine in adults 60 years of age and older. *Vaccine*, *28*(1), 228–234. https://doi.org/10.1016/j.vaccine.2009.09.092

Dei, V., & Sebastian, M. S. (2018). Is healthcare really equal for all? Assessing the horizontal and vertical equity in healthcare utilisation among older Ghanaians. *International Journal for Equity in Health*, *17*(1), 86. https://doi.org/10.1186/s12939-018-0791-3

Deist, M., & Greeff, A. P. (2017). Living with a parent with dementia: A family resilience study. *Dementia*, *16*(1), 126–141. https://doi.org/10.1177/1471301215621853

Desormais, I., Aboyans, V., Guerchet, M., Ndamba-Bandzouzi, B., Mbelesso, P., Dantoine, T., Mohty, D., Marin, B., Preux, P. M., & Lacroix, P. (2015). Prevalence of peripheral artery disease in the elderly population in urban and rural areas of Central Africa: The EPIDEMCA study. *European Journal of Preventive Cardiology*, *22*(11), 1462–1472. https://doi.org/10.1177/2047487314557945

Dewhurst, F., Dewhurst, M. J., Gray, W. K., Aris, E., Orega, G., Howlett, W., Warren, N., & Walker, R. W. (2013). The prevalence of neurological disorders in older people in Tanzania. *Acta Neurologica Scandinavica*, *127*(3), 198–207. https://doi.org/10.1111/j.1600-0404.2012.01709.x

Dewhurst, Felicity, Dewhurst, M. J., Gray, W. K., Chaote, P., Howlett, W., Orega, G., & Walker, R. W. (2012). Rates of diagnosis and treatment of neurological disorders within a prevalent population of community-dwelling elderly people in sub-Saharan Africa. *Journal of Epidemiology and Global Health*, *2*(4), 207–214. https://doi.org/10.1016/j.jegh.2012.11.002

Dewhurst, Felicity, Dewhurst, M. J., Gray, W. K., Orega, G., Howlett, W., Chaote, P., Dotchin, C., Longdon, A. R., Paddick, S. M., & Walker, R. W. (2012). The prevalence of disability in older people in Hai, Tanzania. *Age and Ageing*, *41*(4), 517–523. https://doi.org/10.1093/ageing/afs054

Dewhurst, M. J., Adams, P. C., Gray, W. K., Dewhurst, F., Orega, G. P., Chaote, P., & Walker, R. W. (2012). Strikingly low prevalence of atrial fibrillation in elderly Tanzanians. *Journal of the American Geriatrics Society*, *60*(6), 1135–1140. https://doi.org/10.1111/j.1532-5415.2012.03963.x

Dewhurst, M. J., Dewhurst, F., Gray, W. K., Chaote, P., Orega, G. P., & Walker, R. W. (2013). The high prevalence of hypertension in rural-dwelling Tanzanian older adults and the disparity between detection, treatment and control: A rule of sixths? *Journal of Human Hypertension*, *27*(6), 374–380. https://doi.org/10.1038/jhh.2012.59

Dia, N., Richard, V., Kiori, D., Cisse, E. H. A. K., Sarr, F. D., Faye, A., Goudiaby, D. G., Diop, O. M., & Niang, M. N. (2014). Respiratory viruses associated with patients older than 50 years presenting with ILI in Senegal, 2009 to 2011. *BMC Infectious Diseases*, *14*(1), 189. https://doi.org/10.1186/1471-2334-

14-189

Diameta, E., Adandom, I., Jumbo, S. U., Nwankwo, H. C., Obi, P. C., & Kalu, M. E. (2018). The Burden Experience of Formal and Informal Caregivers of Older Adults With Hip Fracture in Nigeria. *Journals.Sagepub.Com*, *4*. https://doi.org/10.1177/2377960818785155

Diamond, T. H., Botha, J. R., Kalk, W. J., & Shires, R. (1986). Primary hyperparathyroidism. A study of 100 patients in Johannesburg. *South African Medical Journal*, *69*(2), 94–97. https://europepmc.org/article/med/3941958

Digenio, A. G., Sim, J. G. M., Krige, K., Stewart, A., Morris, R., Dowdeswell, R. J., & Padayachee, G. N. (1991). *The Johannesburg cardiac rehabilitation programme*. South African Medical Journal. https://pubmed.ncbi.nlm.nih.gov/1996433/

Dobseu, R., Nanfack, A., Kowo, M., Ambada, G., Kamgaing, R., Chenwi, C., ... & Ndjolo, A. (2020). Evaluation of hepatic fibrosis in HIV/HCV co-infected individuals in Yaoundé, Cameroon: usefulness of APRI score in resource-constrained settings. *BMC Infectious Diseases*, *20*(1), 1-7.

Dotchin, C. L., Paddick, S. M., Gray, W. K., Kisoli, A., Orega, G., Longdon, A. R., Chaote, P., Dewhurst, F., Dewhurst, M., & Walker, R. W. (2015). The association between disability and cognitive impairment in an elderly Tanzanian population. *Journal of Epidemiology and Global Health*, *5*(1), 57–64. https://doi.org/10.1016/j.jegh.2014.09.004

Drah, B. B. (2014). "Older Women", Customary Obligations and Orphan Foster Caregiving: The Case of Queen Mothers in Manya Klo, Ghana. *Journal of Cross-Cultural Gerontology*, *29*(2), 211–229. https://doi.org/10.1007/s10823-014-9232-y

du Rand, P., & Engelbrecht, K. (2001). Needs of frail elderly people in informal settlements. *Curationis*, *24*(4), 10–16. https://doi.org/10.4102/curationis.v24i4.869

Eales, C. J., & Stewart, A. V. (1996). The exercise capacity of three socio-economic groups of elderly hypertensive patients. *Physiotherapy Research International*, *1*(4), 255–264. https://doi.org/10.1002/pri.69

Eales, C. J., & Stewart, A. V. (1997). *A proposed field test for evaluating fitness in elderly hypertensive patients*. South African Journal of Physiotherapy. https://doi.org/10.4102/sajp.v53i2.1362

Eduardo, E., Lamb, M. R., Kandula, S., Howard, A., Mugisha, V., Kimanga, D., Kilama, B., El-Sadr, W., & Elul, B. (2014). Characteristics and Outcomes among Older HIV-Positive Adults Enrolled in HIV Programs in Four Sub-Saharan African Countries. *PLoS ONE*, *9*(7), e103864.

https://doi.org/10.1371/journal.pone.0103864

Elk, R., Swartz, L., & Gillis, L. S. (1983). *The Coloured elderly in Cape Town. A psychosocial, psychiatric and medical community survey. Part I. Introduction and psychosocial data*. South African Medical Journal. https://pubmed.ncbi.nlm.nih.gov/6648741/

Enikuomehin, A., Kolawole, B. A., Soyoye, O. D., Adebayo, J. O., & Ikem, R. T. (2020). Influence of gender on the distribution of type 2 diabetic complications at the obafemi awolowo teaching hospital, Ile-Ife, Nigeria. *African Health Sciences*, *20*(1), 294-307.

Eze, B. U., Mbaeri, T. U., & Orakwe, J. C. (2020). Anterior bladder wall thickness, post-void urine residue, and bladder emptying efficiency as indicators of bladder dysfunction in Nigerian men with benign prostatic hyperplasia. *Nigerian Journal of Clinical Practice*, *23*(9), 1215.

Ezenwa, E. V., Osaghae, S. O., Ozah, E. O., & Okparanta, G. (2020). Apical peri-prostatic nerve block versus intra-rectal xylocaine gel for trans-rectal ultrasound guided prostate biopsy among Nigerian patients: A prospective randomized study. *Nigerian Journal of Clinical Practice*, *23*(9), 1183

Faber, M., Kriek, J. A., Wolmarans, P., van Staden, E., Benadé, A. J., Labadarios, D., Slazus, W., & Taljaard, J. J. (1992). Dietary patterns and nutritional status in free-living older white men with established vascular disease. *South African Medical Journal = Suid-Afrikaanse Tydskrif Vir Geneeskunde*, *82*(4), 232–236. http://www.ncbi.nlm.nih.gov/pubmed/1411818

Fakoya, O. O., Abioye-Kuteyi, E. A., Bello, I. S., Oyegbade, O. O., Olowookere, S. A., & Ezeoma, I. T. (2018). Determinants of Quality of Life of Elderly Patients Attending a General Practice Clinic in Southwest Nigeria. *International Quarterly of Community Health Education*, *39*(1), 3–7. https://doi.org/10.1177/0272684X18781781

Fantahun, M., Berhane, Y., Högberg, U., Wall, S., & Byass, P. (2009). Ageing of a rural Ethiopian population: who are the survivors? *Public Health*, *123*(4), 326–330. https://doi.org/10.1016/j.puhe.2008.10.019

Fawale, M. B., Ismaila, I. A., Mustapha, A. F., Komolafe, M. A., & Ibigbami, O. (2017). Correlates of sleep quality and sleep duration in a sample of urban-dwelling elderly Nigerian women. *Sleep Health*, *3*(4), 257–262. https://doi.org/10.1016/j.sleh.2017.05.008

Ferrari, R., Ford, I., Greenlaw, N., Tardif, J. C., Tendera, M., Abergel, H., Fox, K., Hu, D., Shalnova, S., & Steg, P. G. (2015). Geographical variations in the prevalence and management of cardiovascular risk factors in outpatients with CAD: Data from the contemporary CLARIFY registry. In *European Journal of Preventive Cardiology* (Vol. 22, Issue 8, pp. 1056–1065). SAGE Publications Inc. https://doi.org/10.1177/2047487314547652

Folorunso, D. F., Dahilo, E. A., Gbujie, I. O., Damtong, F. M., Quadri, O. A., Nwakwo, B. E., ... & Nwaorgu, O. B. (2020). Age-related hearing loss at Gwagwalada area council of federal capital territory, Abuja. *Nigerian Journal of Clinical Practice*, *23*(11), 1494.

Forrest, B. D., Steele, A. D., Hiemstra, L., Rappaport, R., Ambrose, C. S., & Gruber, W. C. (2011). A prospective, randomized, open-label trial comparing the safety and efficacy of trivalent live attenuated and inactivated influenza vaccines in adults 60 years of age and older. *Vaccine*, *29*(20), 3633–3639. https://doi.org/10.1016/j.vaccine.2011.03.029

Frost, L., Liddie Navarro, A., Lynch, M., Campbell, M., Orcutt, M., Trelfa, A., Dotchin, C., & Walker, R. (2015). Care of the Elderly: Survey of Teaching in an Aging Sub-Saharan Africa. *Gerontology and Geriatrics Education*, *36*(1), 14–29. https://doi.org/10.1080/02701960.2014.925886

Gatimu, S. M., Milimo, B. W., & Sebastian, M. S. (2016). Prevalence and determinants of diabetes among older adults in Ghana. *BMC Public Health*, *16*(1), 1–12. https://doi.org/10.1186/s12889-016-3845-8

Gaziano, T. A., Abrahams-Gessel, S., Gomez-Olive, F. X., Wade, A., Crowther, N. J., Alam, S., Manne-Goehler, J., Kabudula, C. W., Wagner, R., Rohr, J., Montana, L., Kahn, K., Bärnighausen, T. W., Berkman, L. F., & Tollman, S. (2017). Cardiometabolic risk in a population of older adults with multiple co-morbidities in rural South Africa: the HAALSI (Health and Aging in Africa: longitudinal studies of INDEPTH communities) study. *BMC Public Health*, *17*(1), 1–10. https://doi.org/10.1186/s12889-017-4117-y

Geerts, G. A. V. M. (2017). Neutral zone or conventional mandibular complete dentures: a randomised crossover trial comparing oral health-related quality of life. *Journal of Oral Rehabilitation*, *44*(9), 702–708. https://doi.org/10.1111/joor.12533

Geyer, S. (2010). Strengths-based groupwork with alcohol dependent older persons: Solution to an age-old problem? *Groupwork*, *20*(1), 63–86. https://doi.org/10.1921/095182410X535935

Gildner, T. E., Liebert, M. A., Kowal, P., Chatterji, S., & Josh Snodgrass, J. (2014). Sleep duration, sleep quality, and obesity risk among older adults from six middle-income countries: findings from the study on global AGEing and adult health (SAGE). *American Journal of Human Biology : The Official Journal of the Human Biology Council*, *26*(6), 803–812. https://doi.org/10.1002/ajhb.22603

Gillis, L. S., & Elk, R. (1981). *Physical and mental incapacity in elderly White persons in Cape Town. A community survey*. South African Medical Journal. https://journals.co.za/content/m_samj/59/5/AJA20785135_13564

Gillis, L. S., Welman, M., Koch, A., & Joyi, M. (1991). *Psychological distress and depression in urbanising elderly black persons*. South African Medical Journal. https://www.ajol.info/index.php/samj/article/view/158373

Golaz, V., Wandera, S. O., & Rutaremwa, G. (2017). Understanding the vulnerability of older adults: Extent of and breaches in support systems in Uganda. *Ageing and Society*, *37*(1), 63–89. https://doi.org/10.1017/S0144686X15001051

Gómez-Olivé, F. X., Thorogood, M., Clark, B., Kahn, K., & Tollman, S. (2013). Self-reported health and health care use in an ageing population in the Agincourt sub-district of rural South Africa. *Global Health Action*, *6*, 19305. https://doi.org/10.3402/gha.v6i0.19305

Gómez-Olivé, F. X., Thorogood, M., Kandala, N. B., Tigbe, W., Kahn, K., Tollman, S., & Stranges, S. (2014). Sleep problems and mortality in rural South Africa: Novel evidence from a low-resource setting. *Sleep Medicine*, *15*(1), 56–63. https://doi.org/10.1016/j.sleep.2013.10.003

Gómez-Olivé, X., Thorogood, M., Bocquier, P., Mee, P., Kahn, K., Berkman, L., & Tollman, S. (2014). Social Conditions and Disability Related to the Mortality of Older People in Rural South Africa. *World Health & Population*, *15*(4), 34–43. https://doi.org/10.12927/whp.2015.24266

Govender, T., & Barnes, J. M. (2014). The Health Status and Unmet Health Needs of Old-Age Pensioners Living in Selected Urban Poor Communities in Cape Town, South Africa. *Journal of Community Health*, *39*(6), 1063–1070. https://doi.org/10.1007/s10900-014-9851-9

Gray, W. K., Dewhurst, F., Dewhurst, M. J., Orega, G., Kissima, J., Chaote, P., & Walker, R. W. (2016). Rates and predictors of three-year mortality in older people in rural Tanzania. *Archives of Gerontology and Geriatrics*, *62*, 36–42. https://doi.org/10.1016/j.archger.2015.10.008

Gray, W. K., Orega, G., Kisoli, A., Rogathi, J., Paddick, S. M., Longdon, A. R., Walker, R. W., Dewhurst, F., Dewhurst, M., Chaote, P., & Dotchin, C. (2017). Identifying Frailty and its Outcomes in Older People in Rural Tanzania. *Experimental Aging Research*, *43*(3), 257–273. https://doi.org/10.1080/0361073X.2017.1298957

Gray, W. K., Paddick, S. M., Kisoli, A., Dotchin, C. L., Longdon, A. R., Chaote, P., Samuel, M., Jusabani, A. M., & Walker, R. W. (2014). Development and validation of the identification and intervention for dementia in elderly Africans (IDEA) study dementia screening instrument. *Journal of Geriatric Psychiatry and Neurology*, *27*(2), 110–118. https://doi.org/10.1177/0891988714522695

Guerchet, M., Houinato, D., Paraiso, M. N., Von Ahsen, N., Nubukpo, P., Otto, M., Clément, J. P., Preux, P. M., & Dartigues, J. F. (2009). Cognitive impairment and dementia in elderly people living in rural Benin, West Africa. *Dementia and Geriatric Cognitive Disorders*, *27*(1), 34–41.

https://doi.org/10.1159/000188661

Guerchet, M., M'Belesso, P., Mouanga, A. M., Bandzouzi, B., Tabo, A., Houinato, D. S., Paraïso, M. N., Cowppli-Bony, P., Nubukpo, P., Aboyans, V., Clément, J. P., Dartigues, J. F., & Preux, P. M. (2010). Prevalence of dementia in elderly living in two cities of central africa: The EDAC survey. *Dementia and Geriatric Cognitive Disorders*, *30*(3), 261–268. https://doi.org/10.1159/000320247

Guerchet, M., Mbelesso, P., Mouanga, A. M., Tabo, A., Bandzouzi, B., Clément, J. P., Lacroix, P., Preux, P. M., & Aboyans, V. (2013). Association between a low ankle-brachial index and dementia in a general elderly population in Central Africa (epidemiology of dementia in Central Africa study). *Journal of the American Geriatrics Society*, *61*(7), 1135–1140. https://doi.org/10.1111/jgs.12310

Guerchet, M., Mouanga, A. M., M'belesso, P., Tabo, A., Bandzouzi, B., Paraïso, M. N., Houinato, D. S., Cowppli-Bony, P., Nubukpo, P., Aboyans, V., Clément, J. P., Dartigues, J. F., & Preux, P. M. (2012). Factors associated with dementia among elderly people living in two cities in Central Africa: The EDAC multicenter study. *Journal of Alzheimer's Disease*, *29*(1), 15–24. https://doi.org/10.3233/JAD-2011-111364

Gureje, O., Ademola, A., & Olley, B. O. (2008). Depression and disability: Comparisons with common physical conditions in the Ibadan Study of Aging. *Journal of the American Geriatrics Society*, *56*(11), 2033–2038. https://doi.org/10.1111/j.1532-5415.2008.01956.x

Gureje, O., Kola, L., & Afolabi, E. (2007). Epidemiology of major depressive disorder in elderly Nigerians in the Ibadan Study of Ageing: a community-based survey. *Lancet*, *370*(9591), 957–964. https://doi.org/10.1016/S0140-6736(07)61446-9

Gureje, O., Ogunniyi, A., & Kola, L. (2006). The profile and impact of probable dementia in a sub-Saharan African community: results from the Ibadan Study of Aging. *Journal of Psychosomatic Research*, *61*(3), 327–333. https://doi.org/10.1016/j.jpsychores.2006.07.016

Gureje, O., Ogunniyi, A., Kola, L., & Abiona, T. (2011). Incidence of and risk factors for dementia in the Ibadan study of aging. *Journal of the American Geriatrics Society*, *59*(5), 869–874. https://doi.org/10.1111/j.1532-5415.2011.03374.x

Gureje, O., Ogunniyi, A., Kola, L., & Afolabi, E. (2006). Functional disability in elderly Nigerians: Results from the Ibadan Study of Aging. *Journal of the American Geriatrics Society*, *54*(11), 1784–1789. https://doi.org/10.1111/j.1532-5415.2006.00944.x

Gureje, O., Oladeji, B. D., Abiona, T., & Chatterji, S. (2014). Profile and determinants of successful aging in the Ibadan study of ageing. *Journal of the American Geriatrics Society*, *62*(5), 836–842. https://doi.org/10.1111/jgs.12802

Gureje, O., Oladeji, B. D., Abiona, T., Makanjuola, V., & Esan, O. (2011). The natural history of insomnia in the Ibadan study of ageing. *Sleep*, *34*(7), 965–973. https://doi.org/10.5665/SLEEP.1138

Gutiérrez, M., Tomás, J. M., Sancho, P., Galiana, L., & Francisco, E. H. (2014). Percepción de calidad de vida en una muestra de ancianos angoleños. *Revista de Psicologia Social*, *29*(2), 346–370. https://doi.org/10.1080/02134748.2014.918825

Gyasi, R. M., Abass, K., & Adu-Gyamfi, S. (2020). How do lifestyle choices affect the link between living alone and psychological distress in older age? Results from the AgeHeaPsyWel-HeaSeeB study. *BMC public health*, *20*(1), 1-9.

Gyasi, R. M., Adam, A. M., & Phillips, D. R. (2019). Financial inclusion, Health-Seeking behavior, and health outcomes among older adults in Ghana. *Research on aging*, *41*(8), 794-820.

Gyasi, R. M., Obeng, B., & Yeboah, J. Y. (2020). Impact of food insecurity with hunger on mental distress among community-dwelling older adults. *PloS one*, *15*(3), e0229840.

Gyasi, R. M., Peprah, P., & Appiah, D. O. (2020). Association of food insecurity with psychological disorders: Results of a population-based study among older people in Ghana. *Journal of affective disorders*, *270*, 75-82.

Gyasi, R. M., & Phillips, D. R. (2020). Risk of psychological distress among community-dwelling older adults experiencing spousal loss in Ghana. *The Gerontologist*, *60*(3), 416-427.

Gyasi, R. M., Phillips, D. R., & Amoah, P. A. (2020). Multidimensional social support and health services utilization among noninstitutionalized older persons in Ghana. *Journal of Aging and Health*, *32*(3-4), 227-239.

Gyasi, R. M., Phillips, D. R., Asante, F., & Boateng, S. (2020). Physical activity and predictors of loneliness in community-dwelling older adults: The role of social connectedness. *Geriatric Nursing*.

Gyasi, R. M., & Phillips, D. R. (2018). Gender, self-rated health and functional decline among community-dwelling older adults. *Archives of Gerontology and Geriatrics*, *77*, 174–183. https://doi.org/10.1016/j.archger.2018.05.010

Hao, G., Bishwajit, G., Tang, S., Nie, C., Ji, L., & Huang, R. (2017). Social participation and perceived depression among elderly population in South Africa. *Clinical Interventions in Aging*, *12*, 971–976. https://doi.org/10.2147/CIA.S137993

Hendrie, H. C., Baiyewu, O., Lane, K. A., Purnell, C., Gao, S., Hake, A., Ogunniyi, A., Gureje, O., Unverzagt, F. W., Murrell, J., Deeg, M. A., & Hall, K. (2013). Homocysteine levels and dementia risk in Yoruba and African Americans. *International Psychogeriatrics*, *25*(11), 1859–1866.

https://doi.org/10.1017/S1041610213001294

Heyns, C. F., Fisher, M., Lecuona, A., & van der Merwe, A. (2011). Prostate cancer among different racial groups in the western cape: Presenting features and management. *South African Medical Journal*, *101*(4), 267–270. https://doi.org/10.7196/samj.4420

Heyns, C. F., Mathee, S., Isaacs, A., Kharwa, A., De Beer, P. M., & Pretorius, M. A. (2003). Problems with prostate specific antigen screening for prostate cancer in the primary healthcare setting in South Africa. *BJU International*, *91*(9), 785–788. https://doi.org/10.1046/j.1464-410X.2003.04241.x

Hien, H., Berthé, A., Drabo, M. K., Konaté, B., Toé, N., Tou, F., Adiara, M., Badini-Kinda, F., Ouédraogo, M., Meda, N., & Macq, J. (2016). *Point of view of older adults on the potentially inappropriate medications prescribing in primary care facilities in Bobo-Dioulasso, Burkina Faso*. Revue d'Epidemiologie et de Sante Publique. https://doi.org/10.1016/j.respe.2015.09.009

Hien, Hervé, Berthé, A., Drabo, M. K., Meda, N., Konaté, B., Tou, F., Badini-Kinda, F., & Macq, J. (2014). Prevalence and patterns of multimorbidity among the elderly in Burkina Faso: Cross-sectional study. *Tropical Medicine and International Health*, *19*(11), 1328–1333. https://doi.org/10.1111/tmi.12377

Hontelez, J. A. C., Lurie, M. N., Newell, M. L., Bakker, R., Tanser, F., Bärnighausen, T., Baltussen, R., & De Vlas, S. J. (2011). Ageing with HIV in South Africa. *AIDS*, *25*(13), 1665–1667. https://doi.org/10.1097/QAD.0b013e32834982ea

Hosegood, V., & Timæus, I. M. (2005). The impact of adult mortality on the living arrangements of older people in rural South Africa. *Ageing and Society*, *25*(3), 431–444. https://doi.org/10.1017/S0144686X0500365X

Houser, N., Baiden, P., & Fuller-Thomson, E. (2017). Diabetes and Hypertension in Congolese Church Personnel: An Emerging Epidemic? *Journal of Community Health*, *42*(3), 453–460. https://doi.org/10.1007/s10900-016-0276-5

Huang, R., Ghose, B., & Tang, S. (2020). Effect of financial stress on self-rereported health and quality of life among older adults in five developing countries: a cross sectional analysis of WHO-SAGE survey. *BMC geriatrics*, *20*(1), 1-12.

Hughes, G. D., Aboyade, O. M., Clark, B. L., & Puoane, T. R. (2013). The prevalence of traditional herbal medicine use among hypertensives living in South African communities. *BMC Complementary and Alternative Medicine*, *13*, 38. https://doi.org/10.1186/1472-6882-13-38

Ibrahim, N., Pozo-Martin, F., & Gilbert, C. (2015). Direct non-medical costs double the total direct costs to patients undergoing cataract surgery in Zamfara state, Northern Nigeria: A case series. *BMC Health Services Research*, *15*(1). https://doi.org/10.1186/s12913-015-0831-2

Ice, G. H., Sadruddin, A. F. A., Vagedes, A., Yogo, J., & Juma, E. (2012). Stress associated with caregiving: An examination of the stress process model among Kenyan Luo elders. *Social Science and Medicine*, *74*(12), 2020–2027. https://doi.org/10.1016/j.socscimed.2012.02.018

Ice, G. H., Yogo, J., Heh, V., & Juma, E. (2010). The Impact of Caregiving on the Health and Well-being of Kenyan Luo Grandparents. *Research on Aging*, *32*(1), 40–66. https://doi.org/10.1177/0164027509348128

Ice, G. H., Zidron, A., & Juma, E. (2008). Health and health perceptions among Kenyan grandparents. *Journal of Cross-Cultural Gerontology*, *23*(2), 111–129. https://doi.org/10.1007/s10823-008-9063-9

Igbokwe, C. C., Ejeh, V. J., Agbaje, O. S., Umoke, P. I. C., Iweama, C. N., & Ozoemena, E. L. (2020). Prevalence of loneliness and association with depressive and anxiety symptoms among retirees in Northcentral Nigeria: a cross-sectional study. *BMC geriatrics*, *20*, 1-10.

Jacobs, P., Richards, J. D., & Ben-Arie, O. (1984). The coloured elderly in Cape Town--a psychosocial, psychiatric and medical community survey. Part IV. Haematological values. *South African Medical Journal = Suid-Afrikaanse Tydskrif Vir Geneeskunde*, *65*(1), 16–18. http://www.ncbi.nlm.nih.gov/pubmed/6695241

Jardim, T. V., Witham, M. D., Abrahams-Gessel, S., Gómez-Olivé, F. X., Tollman, S., Berkman, L., & Gaziano, T. A. (2018). Cardiovascular Disease Profile of the Oldest Adults in Rural South Africa: Data from the HAALSI Study (Health and Aging in Africa: Longitudinal Studies of INDEPTH Communities). *Journal of the American Geriatrics Society*, *66*(11), 2151–2157. https://doi.org/10.1111/jgs.15567

Jésus, P., Guerchet, M., Mouanga, A. M., Mbelesso, P., Preux, P. M., & Desport, J. C. (2013). *Factors associated with nutritional status in elderly living in two cities of central Africa: The EDAC study*. Annals of Nutrition and Metabolism. http://www.embase.com/search/results?subaction=viewrecord&from=export&id=L71179590%5Cnhttp://dx.doi.org/10.1159/000354245%5Cnhttp://sfx.hul.harvard.edu/sfx_local?sid=EMBASE&issn=02506807&id=doi:10.1159%2F000354245&atitle=Factors+associated+with+nutritiona

Jingi, A. M., Kuate, L. M., & Noubiap, J. J. (2017). A case management of hypertension in the elderly in Africa: Lessons from granny. *Pan African Medical Journal*, *26*. https://doi.org/10.11604/pamj.2017.26.165.10660

Joffe, B. I., Seftel, H. C., Goldberg, R. C., Bersohn, I., & Hackeng, W. H. (1975). *Metabolic bone disease in the elderly. Biochemical studies in three different racial groups living in South Africa*. South African Medical Journal. https://pubmed.ncbi.nlm.nih.gov/1154141/

Kakongi, N., Rukundo, G. Z., Gelaye, B., Wakida, E. K., Obua, C., & Okello, E. S. (2020). Exploring pathways to Hospital Care for Patients with Alzheimer's disease and related dementias in rural South Western Uganda. *BMC health services research*, *20*, 1-12.

Kailembo, A., Preet, R., & Stewart Williams, J. (2016). Common risk factors and edentulism in adults, aged 50 years and over, in China, Ghana, India and South Africa: Results from the WHO Study on global AGEing and adult health (SAGE). *BMC Oral Health*, *17*(1). https://doi.org/10.1186/s12903-016-0256-2

Kalu, M. E., Vlachantoni, A., & Norman, K. E. (2019). Knowledge about risk factors for falls and practice about fall prevention in older adults among physiotherapists in Nigeria. *Physiotherapy Research International*, *24*(1), e1742. https://doi.org/10.1002/pri.1742

Kalula, S.Z., Ferreira, M., Thomas, K. G. F., de Villiers, L., Joska, J. A., Geffen, L. N., Kalula, S. Z., Ferreira, M., Thomas, K. G. F., de Villiers, L., Joska, J. A., & Geffen, L. N. (2010). *Profile and management of patients at a memory clinic*. South African Medical Journal. http://www.embase.com/search/results?subaction=viewrecord&from=export&id=L359159661%5Cnhttp://sfx.hul.harvard.edu/sfx_local?sid=EMBASE&issn=02569574&id=doi:&atitle=Profile+and+management+of+patients+at+a+memory+clinic&stitle=S.+Afr.+Med.+J.&title=South+Af

Kalula, Sebastiana Z., Ferreira, M., Swingler, G. H., & Badri, M. (2015). Ethnic differences in rates and causes of falls in an urban community-dwelling older population in South Africa. In *Journal of the American Geriatrics Society* (Vol. 63, Issue 2, pp. 403–404). Blackwell Publishing Inc. https://doi.org/10.1111/jgs.13277

Kalula, Sebastiana Z., Ferreira, M., Swingler, G. H., Badri, M., & Sayer, A. A. (2017). Methodological challenges in a study on falls in an older population of cape town, South Africa. *African Health Sciences*, *17*(3), 912–922. https://doi.org/10.4314/ahs.v17i3.35

Kalula, Sebastiana Zimba, de Villiers, L., Ross, K., & Ferreira, M. (2006). *Management of older patients presenting after a fall - An accident and emergency department audit*. South African Medical Journal. https://doi.org/10.7196/SAMJ.1204

Kalula, Sebastiana Zimba, Ferreira, M., Swingler, G. H., & Badri, M. (2016). Risk factors for falls in older adults in a South African Urban Community Physical functioning, physical health and activity. *BMC Geriatrics*, *16*(1). https://doi.org/10.1186/s12877-016-0212-7

Karstaedt, A. S., & Bolhaar, M. (2014). Tuberculosis in older adults in Soweto, South Africa. *International Journal of Tuberculosis and Lung Disease*, *18*(10), 1220–1222. https://doi.org/10.5588/ijtld.14.0210

Kellett-Wright, J., Flatt, A., Eaton, P., Urasa, S., Howlett, W., Dekker, M., ... & Paddick, S. M. (2020). Screening for HIV-Associated Neurocognitive Disorder (HAND) in Adults Aged 50 and Over Attending a Government HIV Clinic in Kilimanjaro, Tanzania. Comparison of the International HIV Dementia Scale (IHDS) and IDEA Six Item Dementia Screen. *AIDS and Behavior*, 1-12.

Kerr, P. P., & Schulze, S. (2004). Factors that influence retirement self-actualisation. *Health SA Gesondheid*, *9*(4). https://doi.org/10.4102/hsag.v9i4.177

Kimuna, S. R., & Makiwane, M. (2007). Older people as resources in South Africa: Mpumalanga households. *Journal of Aging and Social Policy*, *19*(1), 97–114. https://doi.org/10.1300/J031v19n01_06

Kinyanda, E., Kuteesa, M., Scholten, F., Mugisha, J., Baisley, K., & Seeley, J. (2016). Risk of major depressive disorder among older persons living in HIV-endemic central and southwestern Uganda. *AIDS Care - Psychological and Socio-Medical Aspects of AIDS/HIV*, *28*(12), 1516–1521. https://doi.org/10.1080/09540121.2016.1191601

Kiplagat, J., Mwangi, A., Chasela, C., & Huschke, S. (2019). Challenges with seeking HIV care services: Perspectives of older adults infected with HIV in western Kenya. *BMC Public Health*, *19*(1), 929. https://doi.org/10.1186/s12889-019-7283-2

Klemz, B. R., Boshoff, C., Mazibuko, N. E., & Asquith, J. A. (2015). The Effect of Altruism on the Spending Behavior of Elderly Caregivers of Family Members With HIV/AIDS in South African Townships. *Health Marketing Quarterly*, *32*(1), 81–95. https://doi.org/10.1080/07359683.2015.1000747

Kobayashi, L. C., Mateen, F. J., Montana, L., Wagner, R. G., Kahn, K., Tollman, S. M., & Berkman, L. F. (2019). Cognitive function and impairment in older, rural south african adults: Evidence from “health and aging in Africa: A longitudinal study of an INDEPTH Community in Rural South Africa.” *Neuroepidemiology*, *52*(1–2), 32–40. https://doi.org/10.1159/000493483

Kolbe-Alexander, T. L., Lambert, E. V., Harkins, J. B., & Ekelund, U. (2006). Comparison of two methods of measuring physical activity in South African older adults. *Journal of Aging and Physical Activity*, *14*(1), 98–114. https://doi.org/10.1123/japa.14.1.98

Kolbe-Alexander, T. L., Pacheco, K., Tomaz, S. A., Karpul, D., & Lambert, E. V. (2015). The relationship between the built environment and habitual levels of physical activity in South African older adults: A pilot study Health behavior, health promotion and society. *BMC Public Health*, *15*(1), 518. https://doi.org/10.1186/s12889-015-1853-8

Koyanagi, A., Veronese, N., Stubbs, B., Vancampfort, D., Stickley, A., Oh, H., Shin, J. Il, Jackson, S., Smith, L., & Lara, E. (2019). Food insecurity is

associated with mild cognitive impairment among middle-aged and older adults in south africa: Findings from a nationally representative survey. *Nutrients*, *11*(4), 749. https://doi.org/10.3390/nu11040749

Kretchy, I. A., Koduah, A., Ohene-Agyei, T., Boima, V., & Appiah, B. (2020). The association between diabetes-related distress and medication adherence in adult patients with type 2 diabetes mellitus: a cross-sectional study. *Journal of diabetes research*, *2020*.

Krige, J. E. J. (2010). *Melanoma in black South Africans*. South African Journal of Surgery. https://doi.org/10.7196/sajs.777

Kunna, R., San Sebastian, M., & Stewart Williams, J. (2017). Measurement and decomposition of socioeconomic inequality in single and multimorbidity in older adults in China and Ghana: Results from the WHO study on global AGEing and adult health (SAGE). *International Journal for Equity in Health*, *16*(1). https://doi.org/10.1186/s12939-017-0578-y

Kuteesa, M. O., Seeley, J., Cumming, R. G., & Negin, J. (2012). Older people living with HIV in Uganda: Understanding their experience and needs. *African Journal of AIDS Research*, *11*(4), 295–305. https://doi.org/10.2989/16085906.2012.754829

Kuteesa, M. O., Wright, S., Seeley, J., Mugisha, J., Kinyanda, E., Kakembo, F., Mwesigwa, R., & Scholten, F. (2014). Experiences of HIV-related stigma among HIV-positive older persons in Uganda – a mixed methods analysis. *Sahara J*, *11*(1), 126–137. https://doi.org/10.1080/17290376.2014.938103

Kyobutungi, C., Egondi, T., & Ezeh, A. (2010). The health and well-being of older people in Nairobi's slums. *Global Health Action*, *3*(1), 2138. https://doi.org/10.3402/gha.v3i0.2138

Kyobutungi, C., Ezeh, A. C., Zulu, E., & Falkingham, J. (2009). HIV/AIDS and the health of older people in the slums of Nairobi, Kenya: Results from a cross sectional survey. *BMC Public Health*, *9*(1), 153. https://doi.org/10.1186/1471-2458-9-153

Lambert, S. D., Bowe, S. J., Livingston, P. M., Heckel, L., Cook, S., Kowal, P., & Orellana, L. (2017). Impact of informal caregiving on older adults' physical and mental health in low-income and middle-income countries: A cross-sectional, secondary analysis based on the WHO's Study on global AGEing and adult health (SAGE). *BMJ Open*, *7*(11), 17236. https://doi.org/10.1136/bmjopen-2017-017236

Lartey, S. T., Magnussen, C. G., Si, L., Boateng, G. O., de Graaff, B., Biritwum, R. B., Minicuci, N., Kowal, P., Blizzard, L., & Palmer, A. J. (2019). Rapidly increasing prevalence of overweight and obesity in older Ghanaian adults from 2007-2015: Evidence from Who-sage waves 1 & 2. *PLoS ONE*, *14*(8). https://doi.org/10.1371/journal.pone.0215045

Lasisi, A. O., & Gureje, O. (2014). *Prevalence and correlates of dizziness in the Ibadan Study of Ageing*. Ear, Nose and Throat Journal. https://pubmed.ncbi.nlm.nih.gov/24817240/

Lasisi, A. O., Abiona, T., & Gureje, O. (2010). Tinnitus in the elderly: Profile, correlates, and impact in the Nigerian study of ageing. *Otolaryngology - Head and Neck Surgery*, *143*(4), 510–515. https://doi.org/10.1016/j.otohns.2010.06.817

Lazenby, J. M., & Olshvevski, J. (2012). Place of death among Botswana's oldest old. *Omega*, *65*(3), 173–187. https://doi.org/10.2190/OM.65.3.a

Le Roux, A. A., & Nadvi, S. S. (2007). Acute extradural haematoma in the elderly. *British Journal of Neurosurgery*, *21*(1), 16–20. https://doi.org/10.1080/02688690601170692

Lekalakala-Mokgele, E. (2014). Understanding of the risk of HIV infection among the elderly in Ga-Rankuwa, South Africa. *Sahara J*, *11*(1), 67–75. https://doi.org/10.1080/17290376.2014.931816

Lekalakala-Mokgele, E. (2014). Understanding of the risk of HIV infection among the elderly in Ga-Rankuwa, South Africa. *Sahara J*, *11*(1), 67–75. https://doi.org/10.1080/17290376.2014.931816

Lekalakala-Mokgele, E. (2016). Exploring gender perceptions of risk of HIV infection and related behaviour among elderly men and women of ga-rankuwa, Gauteng province, South Africa. *Sahara J*, *13*(1), 88–95. https://doi.org/10.1080/17290376.2016.1218790

Lekpa, F. K., Ndongo, S., Ka, O., Zeba, D., Compaoré, C., Pouye, A., Ka, M. M., & Diop, T. M. (2013). Socio-demographic and clinical profile of chronic pain with neuropathic characteristics in sub-Saharan African elderly. *European Journal of Pain*, *17*(6), 939–943. https://doi.org/10.1002/j.1532-2149.2012.00243.x

Lenger, V., de Viliers, C., & Louw, S. J. (1996). *Informant questionnaires as screening measures to detect dementia. A pilot study in the South African context.* South African Medical Journal = Suid-Afrikaanse Tydskrif Vir Geneeskunde. https://pubmed.ncbi.nlm.nih.gov/9180766/

Leuning, C., Small, L., & Van Dyk, A. (2000). Meanings and expressions of care and caring for elders in urban Namibian families: *Curationis*, *23*(3). https://doi.org/10.4102/curationis.v23i3.711

Lewis, E. G., Banks, J. E., Paddick, S., Dotchin, C., Gray, W., Walker, R., & Urasa, S. (2017). RISK FACTORS FOR PREVALENT DELIRIUM IN OLDER

MEDICAL INPATIENTS IN TANZANIA. *Innovation in Aging*, *1*(suppl_1), 1283–1284. https://doi.org/10.1093/geroni/igx004.4685

Longdon, A. R., Paddick, S. M., Kisoli, A., Dotchin, C., Gray, W. K., Dewhurst, F., Chaote, P., Teodorczuk, A., Dewhurst, M., Jusabani, A. M., & Walker, R. (2013). The prevalence of dementia in rural Tanzania: A cross-sectional community-based study. *International Journal of Geriatric Psychiatry*, *28*(7), 728–737. https://doi.org/10.1002/gps.3880

Lopes Ibanez-Gonzalez, D., & Tollman, S. M. (2015). Clinics and Churches: Lifeworlds and health-seeking practices of older women with noncommunicable disease in rural South Africa. *BMC International Health and Human Rights*, *15*(1). https://doi.org/10.1186/s12914-015-0051-1

Lowis, M. J., & Hughes, J. (1997). A comparison of the effects of sacred and secular music on elderly people. *Journal of Psychology: Interdisciplinary and Applied*, *131*(1), 45–55. https://doi.org/10.1080/00223989709603503

Lwanga, I., Nabaggala, M. S., Kiragga, A., Calcagno, A., Guaraldi, G., Lamorde, M., & Castelnuovo, B. (2020). Implementing routine physical function screening among elderly HIV-positive patients in Uganda. *AIDS care*, *32*(11), 1467-1470.

Mabaso, R. G., & Oduntan, O. A. (2016). Knowledge and practices related to diabetes mellitus among adults with diabetes in the Mopani District, Limpopo Province, South Africa. *African Vision and Eye Health*, *75*(1). https://doi.org/10.4102/aveh.v75i1.324

Mabeku, L. B. K., Ngamga, M. L. N., & Leundji, H. (2020). Helicobacter pylori infection, a risk factor for Type 2 diabetes mellitus: a hospital-based cross-sectional study among dyspeptic patients in Douala-Cameroon. *Scientific reports*, *10*(1), 1-11.

Macia, E., Duboz, P., & Gueye, L. (2012). Prevalence, awareness, treatment and control of hypertension among adults 50 years and older in Dakar, Senegal. *Cardiovascular Journal of Africa*, *23*(5), 265–269. https://doi.org/10.5830/CVJA-2011-039

Macia, Enguerran, Duboz, P., Montepare, J. M., & Gueye, L. (2012). Age identity, self-rated health, and life satisfaction among older adults in Dakar, Senegal. *European Journal of Ageing*, *9*(3), 243–253. https://doi.org/10.1007/s10433-012-0227-7

Macia, Enguerran, Duboz, P., Montepare, J. M., & Gueye, L. (2015). Exploring Life Satisfaction Among Older Adults in Dakar. *Journal of Cross-Cultural Gerontology*, *30*(4), 377–391. https://doi.org/10.1007/s10823-015-9275-8

Manne-Goehler, J., Kakuhikire, B., Abaasabyoona, S., Bärnighausen, T. W., Okello, S., Tsai, A. C., & Siedner, M. J. (2019). Depressive Symptoms Before and After Antiretroviral Therapy Initiation Among Older-Aged Individuals in Rural Uganda. *AIDS and Behavior*, *23*(3), 564–571.

https://doi.org/10.1007/s10461-018-2273-4

Maritz, M., Fourie, C. M. T., Van Rooyen, J. M., & Schutte, A. E. (2018). Evaluating several biomarkers as predictors of aortic stiffness in young and older Africans, not consuming alcohol based on self-report. *Diabetes Research and Clinical Practice*, *142*, 312–320. https://doi.org/10.1016/j.diabres.2018.05.048

Martinez, P., Lien, L., Landheim, A., Kowal, P., & Clausen, T. (2014). Quality of life and social engagement of alcohol abstainers and users among older adults in South Africa. *BMC Public Health*, *14*(1), 316. https://doi.org/10.1186/1471-2458-14-316

Matlho, K., Randell, M., Lebelonyane, R., Kefas, J., Driscoll, T., & Negin, J. (2019). HIV prevalence and related behaviours of older people in Botswana — secondary analysis of the Botswana AIDS Impact Survey (BAIS) IV. *African Journal of AIDS Research*, *18*(1), 18–26. https://doi.org/10.2989/16085906.2018.1552162

Matovu, S. N., & Wallhagen, M. I. (2020). Perceived Caregiver Stress, Coping, and Quality of Life of Older Ugandan Grandparent-Caregivers. *Journal of Cross-Cultural Gerontology*, *35*(3), 311-328.

Matovu, S., Rankin, S., & Wallhagen, M. (2020). Ugandan Jajjas: Antecedents and rewards of caring for grandchildren in the context of HIV. *International Journal of Older People Nursing*, *15*(2), e12304.

Mbada, C. E., Adeniyi, O. A., Idowu, O. A., Fatoye, C. T., Odole, A. C., & Fatoye, F. (2020). Cross-cultural adaptation and psychometric evaluation of the Yoruba version of the Back beliefs questionnaire among patients with chronic low-back pain. *Health and quality of life outcomes*, *18*(1), 1-9.

Mbui, J. M., Oluka, M. N., Guantai, E. M., Sinei, K. A., Achieng, L., Baker, A., Jande, M., Massele, A., & Godman, B. (2017). Prescription patterns and adequacy of blood pressure control among adult hypertensive patients in Kenya; findings and implications. *Expert Review of Clinical Pharmacology*, *10*(11), 1263–1271. https://doi.org/10.1080/17512433.2017.1371590

McKinnon, B., Harper, S., & Moore, S. (2013). The relationship of living arrangements and depressive symptoms among older adults in sub-Saharan Africa. *BMC Public Health*, *13*(1), 682. https://doi.org/10.1186/1471-2458-13-682

Meiring, P., Blake, A. J., & Grobbelaar, J. P. (1983). *Identification and definition of the geriatric patient in a teaching hospital*. South African Medical Journal.

https://pubmed.ncbi.nlm.nih.gov/6623267/

Menyanu, E., Charlton, K. E., Ware, L. J., Russell, J., Biritwum, R., & Kowal, P. (2017). Salt use behaviours of ghanaians and south africans: A comparative study of knowledge, attitudes and practices. *Nutrients*, *9*(9), 1211. https://doi.org/10.3390/nu9090939

Mhaka-Mutepfa, M., Cumming, R., & Mpofu, E. (2014). Grandparents Fostering Orphans: Influences of Protective Factors on Their Health and Well-Being. *Health Care for Women International*, *35*(7–9), 1022–1039. https://doi.org/10.1080/07399332.2014.916294

Minicuci, N., Biritwum, R. B., Mensah, G., Yawson, A. E., Naidoo, N., Chatterji, S., & Kowal, P. (2014). Sociodemographic and socioeconomic patterns of chronic non-communicable disease among the older adult population in Ghana. *Global Health Action*, *7*(1). https://doi.org/10.3402/gha.v7.21292

Mkhonto, F., & Hanssen, I. (2018). When people with dementia are perceived as witches. Consequences for patients and nurse education in South Africa. *Journal of Clinical Nursing*, *27*(1–2), e169–e176. https://doi.org/10.1111/jocn.13909

Molete, M. P., Yengopal, V., & Moorman, J. (2014). *Oral health needs and barriers to accessing care among the elderly in Johannesburg*. SADJ : Journal of the South African Dental Association = Tydskrif van Die Suid-Afrikaanse Tandheelkundige Vereniging. https://pubmed.ncbi.nlm.nih.gov/26548224/

Moreno-Agostino, D., Stone, A. A., Schneider, S., Koskinen, S., Leonardi, M., Naidoo, N., ... & Chatterji, S. (2019). Are retired people higher in experiential wellbeing than working older adults? A time use approach. *Emotion*.

Mtowa, A., Gerritsen, A. A. M., Mtenga, S., Mwangome, M., & Geubbels, E. (2017). Socio-demographic inequalities in HIV testing behaviour and HIV prevalence among older adults in rural Tanzania, 2013. *AIDS Care - Psychological and Socio-Medical Aspects of AIDS/HIV*, *29*(9), 1162–1168. https://doi.org/10.1080/09540121.2017.1308462

Muchiri, J. W., Gericke, G. J., & Rheeder, P. (2012). Needs and preferences for nutrition education of type 2 diabetic adults in a resource-limited setting in South Africa. *Health SA Gesondheid*, *17*(1), 13. https://doi.org/10.4102/hsag.v17i1.614

Mugisha, J. O., Schatz, E. J., Hansen, C., Leary, E., Negin, J., Kowal, P., & Seeley, J. (2018). Social engagement and survival in people aged 50 years and over living with HIV and without HIV in Uganda: a prospective cohort study. *African Journal of AIDS Research*, *17*(4), 333–340. https://doi.org/10.2989/16085906.2018.1542322

Mugisha, J. O., Schatz, E. J., Negin, J., Mwaniki, P., Kowal, P., & Seeley, J. (2017). Timing of Most Recent Health Care Visit by Older People Living With and

Without HIV. *International Journal of Aging & Human Development*, *85*(1), 18–32. https://doi.org/10.1177/0091415016680071

Mugisha, J. O., Schatz, E. J., Randell, M., Kuteesa, M., Kowal, P., Negin, J., & Seeley, J. (2016). Chronic disease, risk factors and disability in adults aged 50 and above living with and without HIV: findings from the Wellbeing of Older People Study in Uganda. *Global Health Action*, *9*(1). https://doi.org/10.3402/gha.v9.31098

Mugisha, J. O., Schatz, E., Seeley, J., & Kowal, P. (2015). Gender perspectives in care provision and care receipt among older people infected and affected by HIV in Uganda. *African Journal of AIDS Research*, *14*(2), 159–167. https://doi.org/10.2989/16085906.2015.1040805

Mushi, D., Rongai, A., Paddick, S. M., Dotchin, C., Mtuya, C., & Walker, R. (2014). Social representation and practices related to dementia in Hai District of Tanzania. *BMC Public Health*, *14*(1). https://doi.org/10.1186/1471-2458-14-260

Mwanyangala, M., Mayombana, C., Urassa, H., Charles, J., Mahutanga, C., Abdullah, S., & Nathan, R. (2010). Health status and quality of life among older adults in rural Tanzania. *Global Health Action*, *3*(1), 2142. https://doi.org/10.3402/gha.v3i0.2142

Myroniuk, T. W. (2017). Marital Dissolutions and the Health of Older Individuals in a Rural African Context. *Journals of Gerontology - Series B Psychological Sciences and Social Sciences*, *72*(4), 656–664. https://doi.org/10.1093/geronb/gbw077

Naah, F. L., Njong, A. M., & Kimengsi, J. N. (2020). Determinants of Active and Healthy Ageing in Sub-Saharan Africa: Evidence from Cameroon. *International journal of environmental research and public health*, *17*(9), 3038.

Nadasen, K. (2008). Life without line dancing and the other activities would be too dreadful to imagine: An increase in social activity for older women. *Journal of Women and Aging*, *20*(3–4), 329–342. https://doi.org/10.1080/08952840801985060

Naidoo, K., & Van Wyk, J. (2019). What the elderly experience and expect from primary care services in KwaZulu-Natal, South Africa. *African Journal of Primary Health Care & Family Medicine*, *11*(1). https://doi.org/10.4102/phcfm.v11i1.2100

Namale, G., Kamacooko, O., Makhoba, A., Mugabi, T., Ndagire, M., Ssanyu, P., ... & Newton, R. (2020). Predictors of 30-day and 90-day mortality among hemorrhagic and ischemic stroke patients in urban Uganda: a prospective hospital-based cohort study. *BMC Cardiovascular Disorders*, *20*(1), 1-11.

Nanji, K., Kherani, I. N., Damji, K. F., Nyenze, M., Kiage, D., & Tennant, M. T. (2020). The muranga teleophthalmology study: A comparison of virtual

(teleretina) assessment with in-person clinical examination to diagnose diabetic retinopathy and age-related macular degeneration in kenya. *Middle East African Journal of Ophthalmology*, *27*(2), 91.

Nash, E. S., & De Meiring, P. V. (1983). *The Coloured elderly in Cape Town - a psychological, psychiatric and medical community survey. Part III. A survey of physical disorders and disabilities*. South African Medical Journal. https://pubmed.ncbi.nlm.nih.gov/6229889/

Ndou, T., van Zyl, G., Hlahane, S., & Goudge, J. (2013). A rapid assessment of a community health worker pilot programme to improve the management of hypertension and diabetes in Emfuleni sub-district of Gauteng Province, South Africa. *Global Health Action*, *6*, 19228. https://doi.org/10.3402/gha.v6i0.19228

Negin, J., Cumming, R., de Ramirez, S. S., Abimbola, S., & Sachs, S. E. (2011). Risk factors for non-communicable diseases among older adults in rural Africa. *Tropical Medicine and International Health*, *16*(5), 640–646. https://doi.org/10.1111/j.1365-3156.2011.02739.x

Negin, J., Geddes, L., Brennan-Ing, M., Kuteesa, M., Karpiak, S., & Seeley, J. (2016). Sexual Behavior of Older Adults Living with HIV in Uganda. *Archives of Sexual Behavior*, *45*(2), 441–449. https://doi.org/10.1007/s10508-015-0582-5

Negin, J., Martiniuk, A., Cumming, R. G., Naidoo, N., Phaswana-Mafuya, N., Madurai, L., Williams, S., & Kowal, P. (2012). Prevalence of HIV and chronic comorbidities among older adults. *AIDS*, *26*(SUPPL.1). https://doi.org/10.1097/QAD.0b013e3283558459

Negin, J., Nemser, B., Cumming, R., Lelerai, E., Amor, Y. Ben, & Pronyk, P. (2012). HIV attitudes, awareness and testing among older adults in Africa. *AIDS and Behavior*, *16*(1), 63–68. https://doi.org/10.1007/s10461-011-9994-y

Negin, J., Randell, M., Raban, M. Z., Nyirenda, M., Kalula, S., Madurai, L., & Kowal, P. (2017). Health expenditure and catastrophic spending among older adults living with HIV. *Global Public Health*, *12*(10), 1282–1296. https://doi.org/10.1080/17441692.2016.1173717

Negin, J., Wariero, J., Cumming, R. G., Mutuo, P., & Pronyk, P. M. (2010). High rates of aids-related mortality among older adults in rural Kenya. *Journal of Acquired Immune Deficiency Syndromes*, *55*(2), 239–244. https://doi.org/10.1097/QAI.0b013e3181e9b3f2

Njemini, R., Meyers, I., Demanet, C., Smitz, J., Sosso, M., & Mets, T. (2002). The prevalence of autoantibodies in an elderly sub-Saharan African population. *Clinical and Experimental Immunology*, *127*(1), 99–106. https://doi.org/10.1046/j.1365-2249.2002.01713.x

Njemini, R., Smitz, J., Demanet, C., Sosso, M., & Mets, T. (2011). Circulating heat shock protein 70 (Hsp70) in elderly members of a rural population from

Cameroon: Association with infection and nutrition. *Archives of Gerontology and Geriatrics*, *53*(3), 359–363. https://doi.org/10.1016/j.archger.2011.01.005

Nwankwo, H. C., Akinrolie, O., Adandom, I., Obi, P. C., Ojembe, B. U., & Kalu, M. E. (2019). The clinical experiences of Nigerian physiotherapists in managing environmental and socioeconomic determinants of mobility for older adults. *Physiotherapy Theory and Practice*. https://doi.org/10.1080/09593985.2019.1700579

Nwankwo, T. O., Umeh, U. A., Aniebue, U. U., Onu, J. U., & Umeh, C. R. (2020). Impact of neoadjuvant chemotherapy in improving operative intervention in the management of cervical cancer in low resource setting: a preliminary report. *The Pan African Medical Journal*, *36*.

Nyanguru, A. C. (2007). *Migration and Aging: The Case of Zimbabwe: Journal of Aging & Social Policy: Vol 19, No 4*. https://www.tandfonline.com/doi/abs/10.1300/J031v19n04_04

Nyirenda, M., Chatterji, S., Falkingham, J., Mutevedzi, P., Hosegood, V., Evandrou, M., Kowal, P., & Newell, M. L. (2012). An investigation of factors associated with the health and well-being of HIV-infected or HIV-affected older people in rural South Africa. *BMC Public Health*, *12*(1), 259. https://doi.org/10.1186/1471-2458-12-259

Nyirenda, M., Newell, M. L., Mugisha, J., Mutevedzi, P. C., Seeley, J., Scholten, F., & Kowal, P. (2013). Health, wellbeing, and disability among older people infected or affected by HIV in Uganda and South Africa. *Global Health Action*, *6*(1). https://doi.org/10.3402/gha.v6i0.19201

Obi, P. C., Nwankwo, H. C., Emofe, D., Adandom, I., & Kalu, M. E. (n.d.). The Experience and Perception of Physiotherapists in Nigeria re: Fall Prevention in Recurrent-Faller Older Adults. The Internet Journal of Allied Health Sciences and Practice. In *nsuworks.nova.edu* (Vol. 17, Issue 2). Retrieved May 11, 2020, from https://nsuworks.nova.edu/ijahsp

Obuku, E. A., Parikh, S. M., Nankabirwa, V., Kakande, N. I., Mafigiri, D. K., Mayanja-Kizza, H., Kityo, C. M., Mugyenyi, P. N., & Salata, R. A. (2013). Determinants of Clinician Knowledge on Aging and HIV/AIDS: A Survey of Practitioners and Policy Makers in Kampala District, Uganda. *PLoS ONE*, *8*(2), e57028. https://doi.org/10.1371/journal.pone.0057028

Ogunyemi, A. O., Odeyemi, K. A., Kanma-Okafor, O. J., & Ladi-Akinyemi, T. W. (2018). *Health-Related Quality of Life of the Elderly in Institutional Care and Non-Institutional Care in Southwestern Nigeria: A Comparative Study.* West African Journal of Medicine. http://www.ncbi.nlm.nih.gov/pubmed/29607474

Ojagbemi, A., Abiona, T., Luo, Z., & Gureje, O. (2018). Symptomatic and Functional Recovery From Major Depressive Disorder in the Ibadan Study of Ageing. *American Journal of Geriatric Psychiatry*, *26*(6), 657–666. https://doi.org/10.1016/j.jagp.2017.12.011

Ojagbemi, A., Bello, T., & Gureje, O. (2016). Cognitive Reserve, Incident Dementia, and Associated Mortality in the Ibadan Study of Ageing. *Journal of the American Geriatrics Society*, *64*(3), 590–595. https://doi.org/10.1111/jgs.14015

Ojagbemi, A., Bello, T., Luo, Z., & Gureje, O. (2017a). Chronic Conditions, New Onset, and Persistent Disability in the Ibadan Study of Aging. *Journals of Gerontology - Series A Biological Sciences and Medical Sciences*, *72*(7), 997–1005. https://doi.org/10.1093/gerona/glv188

Ojagbemi, A., Bello, T., Luo, Z., & Gureje, O. (2017b). Living Conditions, Low Socioeconomic Position, and Mortality in the Ibadan Study of Aging. *Journals of Gerontology - Series B Psychological Sciences and Social Sciences*, *72*(4), 646–655. https://doi.org/10.1093/geronb/gbv093

Ojagbemi, A., D'Este, C., Verdes, E., Chatterji, S., & Gureje, O. (2015). Gait speed and cognitive decline over 2 years in the Ibadan study of aging. *Gait and Posture*, *41*(2), 736–740. https://doi.org/10.1016/j.gaitpost.2015.01.011

Ojagbemi, A., Oladeji, B., Abiona, T., & Gureje, O. (2013). Suicidal behaviour in old age - results from the Ibadan study of ageing. *BMC Psychiatry*, *13*, 80. https://doi.org/10.1186/1471-244X-13-80

Ojembe, B. U., & Kalu, M. E. (2018). Describing reasons for loneliness among older people in Nigeria. *Journal of Gerontological Social Work*, *61*(6), 640–658. https://doi.org/10.1080/01634372.2018.1487495

Ojembe, B.U. & Kalu, M. E. (2019). *Television, radio, and telephone: Tools for reducing loneliness among older adults in Nigeria*. Gerontechnology Journal. http://journal.gerontechnology.org/currentIssueContent.aspx?aid=1795

Okunade, K. S., Adetuyi, I. E., Adenekan, M., Ohazurike, E., & Anorlu, R. I. (2020). Risk predictors of early recurrence in women with epithelial ovarian cancer in Lagos, Nigeria. *The Pan African Medical Journal*, *36*.

Oladeji, B. D., Makanjuola, V. A., Esan, O. B., & Gureje, O. (2011). Chronic pain conditions and depression in the Ibadan Study of Ageing. *International Psychogeriatrics*, *23*(6), 923–929. https://doi.org/10.1017/S1041610210002322

Olamoyegun, M. A., Raimi, T. H., Ala, O. A., & Fadare, J. O. (2020). Mobile phone ownership and willingness to receive mHealth services among patients

with diabetes mellitus in South-West, Nigeria. *The Pan African Medical Journal*, *37*.

Olatayo, A. A., Kubwa, O. O., & Adekunle, A. E. (2015). Sexuality in Nigerian older adults. *The Pan African Medical Journal*, *22*, 315. https://doi.org/10.11604/pamj.2015.22.315.7617

Ologe, F. E., Segun-Busari, S., Abdulraheem, I. S., & Afolabi, A. O. (2005). Ear diseases in elderly hospital patients in Nigeria. *Journals of Gerontology - Series A Biological Sciences and Medical Sciences*, *60*(3), 404–406. https://doi.org/10.1093/gerona/60.3.404

Okoh, A. E., Akinrolie, O., Bell-Gam, H. I., Adandom, I., Ibekaku, M. C., & Kalu, M. E. (2020). Nigerian healthcare workers' perception of transdisciplinary approach to older adults' care: A qualitative case study. *International Journal of Care Coordination*, *23*(2-3), 92-106.

Okoye, E. C., Akosile, C. O., Maruf, F. A., Onwuakagba, I. U., & Urama, S. T. (2020). Validation of Igbo version of the modified falls efficacy scale among community-dwelling older adults: a validation study. *Health and Quality of Life Outcomes*, *18*(1), 1-9.

Omenai, S. A., Ezenkwa, U. S., & Ajani, M. A. (2020). Mortality patterns in patients with diabetes mellitus at a Nigerian tertiary hospital: A 10-Year autopsy study. *Nigerian Postgraduate Medical Journal*, *27*(2), 83.

Onadja, Y., Atchessi, N., Soura, B. A., Rossier, C., & Zunzunegui, M. V. (2013). Gender differences in cognitive impairment and mobility disability in old age: A cross-sectional study in Ouagadougou, Burkina Faso. *Archives of Gerontology and Geriatrics*, *57*(3), 311–318. https://doi.org/10.1016/j.archger.2013.06.007

Onakpoya, O. H., Adeoye, A. O., Adegbehingbe, B. O., Badmus, S. A., Adewara, B. A., Awe, O. O., & Udonwa, P. A. (2020). Intraocular pressure variation after conventional extracapsular cataract extraction, manual small incision cataract surgery and phacoemulsification in an indigenous black population. *The Pan African Medical Journal*, *36*.

Onwuchewa, A., BellGam, H., & Asekomeh, G. (2009). Stroke at the University of Port Harcourt Teaching Hospital, Rivers State, Nigeria. *Tropical Doctor*, *39*(3), 150–152. https://doi.org/10.1258/td.2008.080285

Onwubiko, S. N., Nwachukwu, N. Z., Muomah, R. C., Okoloagu, N. M., Ngwegu, O. M., & Nwachukwu, D. C. (2020). Factors associated with depression and anxiety among glaucoma patients in a tertiary hospital South-East Nigeria. *Nigerian journal of clinical practice*, *23*(3), 315.

Osberg, L. (2015). The Hunger of Old Women in Rural Tanzania: Can Subjective Data Improve Poverty Measurement? *Review of Income and Wealth*, *61*(4), 723–738. https://doi.org/10.1111/roiw.12128

Oshi, D. C., Oshi, S. N., Alobu, I., & Ukwaja, K. N. (2014). Profile and Treatment Outcomes of Tuberculosis in the Elderly in Southeastern Nigeria, 2011–

2012. *PLoS ONE*, *9*(11), e111910. https://doi.org/10.1371/journal.pone.0111910

Ottie-Boakye, D. (2020). Coverage of non-receipt of cash transfer (Livelihood Empowerment Against Poverty) and associated factors among older persons in the Mampong Municipality, Ghana–a quantitative analysis. *BMC geriatrics*, *20*(1), 1-10.

Otitoola, O. C., Oldewage-Theron, W. N., & Egal, A. A. (2015). Trends in the development of obesity in elderly day care attendees in Sharpeville, South Africa, from 2007-2011. *South African Journal of Clinical Nutrition*, *28*(1), 12–17. https://doi.org/10.1080/16070658.2015.11734520

Padayachey, U., Ramlall, S., & Chipps, J. (2017). Depression in older adults: prevalence and risk factors in a primary health care sample. *South African Family Practice*, *59*(2), 61–66. https://doi.org/10.1080/20786190.2016.1272250

Paddick, S. M., Lewis, E. G., Duinmaijer, A., Banks, J., Urasa, S., Tucker, L., Kisoli, A., Cletus, J., Lissu, C., Kissima, J., Dotchin, C., Gray, W. K., Muaketova-Ladinska, E., Cosker, G., & Walker, R. W. (2018). Identification of delirium and dementia in older medical inpatients in Tanzania: A comparison of screening and diagnostic methods. *Journal of the Neurological Sciences*, *385*, 156–163. https://doi.org/10.1016/j.jns.2017.12.006

Paddick, S.-M., Kisoli, A., Longdon, A., Dotchin, C., Gray, W. K., Chaote, P., Teodorczuk, A., & Walker, R. (2015). The prevalence and burden of behavioural and psychological symptoms of dementia in rural Tanzania. *International Journal of Geriatric Psychiatry*, *30*(8), 815–823. https://doi.org/10.1002/gps.4218

Paddick, Stella-Maria, Kisoli, A., Mkenda, S., Mbowe, G., Gray, W. K., Dotchin, C., Ogunniyi, A., Kisima, J., Olakehinde, O., Mushi, D., & Walker, R. W. (2017). Adaptation and validation of the Alzheimer's Disease Assessment Scale – Cognitive (ADAS-Cog) in a low-literacy setting in sub-Saharan Africa. *Acta Neuropsychiatrica*, *29*(4), 244–251. https://doi.org/10.1017/neu.2016.65

Paquissi, F. C., Cuvinje, A. B. P., & Cuvinje, A. B. (2016). Prevalence of Peripheral Arterial Disease among Adult Patients Attending Outpatient Clinic at a General Hospital in South Angola. *Scientifica*, *2016*. https://doi.org/10.1155/2016/2520973

Parag, Y., & Buccimazza, I. (2016). How long are elderly patients followed up with mammography after the diagnosis of breast cancer? A single-centre experience in a developing country. *South African Medical Journal*, *106*(7), 721–723. https://doi.org/10.7196/SAMJ.2016.v106i7.10405

Parmar, D., Williams, G., Dkhimi, F., Ndiaye, A., Asante, F. A., Arhinful, D. K., & Mladovsky, P. (2014). Enrolment of older people in social health protection programs in West Africa - Does social exclusion play a part? *Social Science and Medicine*, *119*, 36–44. https://doi.org/10.1016/j.socscimed.2014.08.011

Payne, C. F., Gómez-Olivé, F. X., Kahn, K., & Berkman, L. (2017). Physical Function in an Aging Population in Rural South Africa: Findings from HAALSI and Cross-National Comparisons with HRS Sister Studies. *Journals of Gerontology - Series B Psychological Sciences and Social Sciences*, *72*(4), 665–679. https://doi.org/10.1093/geronb/gbx030

Payne, C. F., Mkandawire, J., & Kohler, H.-P. (2013). Disability Transitions and Health Expectancies among Adults 45 Years and Older in Malawi: A Cohort-Based Model. *PLoS Medicine*, *10*(5), e1001435. https://doi.org/10.1371/journal.pmed.1001435

Payne, C. F., Wade, A., Kabudula, C. W., Davies, J. I., Chang, A. Y., Gomez-Olive, F. X., Kahn, K., Berkman, L. F., Tollman, S. M., Salomon, J. A., & Witham, M. D. (2017). Prevalence and correlates of frailty in an older rural African population: Findings from the HAALSI cohort study. *BMC Geriatrics*, *17*(1), 293. https://doi.org/10.1186/s12877-017-0694-y

Peil, M., Ekpenyong, S. K., & Oyeneye, O. Y. (1988). Going Home: Migration Careers of Southern Nigerians <sup/>. *International Migration Review*, *22*(4), 563–585. https://doi.org/10.1177/019791838802200402

Peltzer, K, & Phaswana-Mafuya, N. (2012). Cognitive functioning and associated factors in older adults in South Africa. *South African Journal of Psychiatry*, *18*(4), 7. https://doi.org/10.4102/sajpsychiatry.v18i4.368

Peltzer, K. (2004). Health beliefs and prescription medication compliance among diagnosed hypertension clinic attenders in a rural South African Hospital. *Curationis*, *27*(3), 15–23. https://doi.org/10.4102/curationis.v27i3.994

Peltzer, K. (2012). Sociodemographic and health correlates of sleep problems and duration in older adults in South Africa. *South African Journal of Psychiatry*, *18*(4), 150–156. https://doi.org/10.7196/SAJP.369

Peltzer, K., & Pengpid, S. (2018). Self-reported sleep duration and its correlates with sociodemographics, health behaviours, poor mental health, and chronic conditions in rural persons 40 years and older in South Africa. *International Journal of Environmental Research and Public Health*, *15*(7). https://doi.org/10.3390/ijerph15071357

Peltzer, K., & Phaswana-Mafuya, N. (2013). Problem drinking and associated factors in older adults in South Africa. *African Journal of Psychiatry (South Africa)*, *16*(2), 104–109. https://doi.org/10.4314/ajpsy.v16i2.13

Peltzer, Karl, & Phaswana-Mafuya, N. (2012a). Patient experiences and health system responsiveness among older adults in South Africa. *Global Health*

Action, *5*, 1–11. https://doi.org/10.3402/gha.v5i0.18545

Peltzer, Karl, & Phaswana-Mafuya, N. (2012b). Tobacco use and associated factors in older adults in South Africa. *Journal of Psychology in Africa*, *22*(2), 283–288. https://doi.org/10.1080/14330237.2012.10820532

Peltzer, Karl, & Phaswana-Mafuya, N. (2012c). Fruit and vegetable intake and associated factors in older adults in South Africa. *Global Health Action*, *5*(1), 18668. https://doi.org/10.3402/gha.v5i0.18668

Peltzer, Karl, & Phaswana-Mafuya, N. (2013a). Depression and associated factors in older adults in South Africa. *Global Health Action*, *6*(1). https://doi.org/10.3402/gha.v6i0.18871

Peltzer, Karl, & Phaswana-Mafuya, N. (2013b). Hypertension and associated factors in older adults in South Africa. *Cardiovascular Journal of Africa*, *24*(3), 66–71. https://doi.org/10.5830/CVJA-2013-002

Peltzer, Karl, & Phaswana-Mafuya, N. (2013c). ARTHRITIS AND ASSOCIATED FACTORS IN OLDER ADULTS IN SOUTH AFRICA GÜNEY AFR‹KA’DAK‹ YAfiLI B‹REYLERDE ARTR‹T VE ‹L‹fiK‹L‹ FAKTÖRLER ÖZ. In *Turkish Journal of Geriatrics* (Vol. 16, Issue 4). http://geriatri.dergisi.org/abstract.php?lang=en&id=770

Peltzer, Karl, & Phaswana-Mafuya, N. (2014). Breast and cervical cancer screening and associated factors among older adult women in South Africa. *Asian Pacific Journal of Cancer Prevention*, *15*(6), 2473–2476. https://doi.org/10.7314/APJCP.2014.15.6.2473

Peltzer, Karl, & Phaswana-Mafuya, N. (2017). Association between visual impairment and low vision and sleep duration and quality among older adults in South Africa. *International Journal of Environmental Research and Public Health*, *14*(7). https://doi.org/10.3390/ijerph14070811

Peltzer, Karl. (2017). Differences in sleep duration among four different population groups of older adults in South Africa. *International Journal of Environmental Research and Public Health*, *14*(5). https://doi.org/10.3390/ijerph14050502

Pengpid, S., & Karl, K. P. (2019). Sedentary behaviour and 12 sleep problem indicators among middle-aged and elderly adults in south africa. *International Journal of Environmental Research and Public Health*, *16*(8). https://doi.org/10.3390/ijerph16081422

Perold, A., & Muller, M. (2000). The composition of old age homes in South Africa in relation to the residents and nursing personnel. *Curationis*, *23*(1), 87–94.

https://doi.org/10.4102/curationis.v23i1.615

Pfttifor, J. M., Ross, F. T., & Solomon, L. (1978). Seasonal variation in serum 25-hydroxycholecalciferol concentrations in elderly South African patients with fractures of femoral neck. *British Medical Journal*, *1*(6116), 826–827. https://doi.org/10.1136/bmj.1.6116.826

Phaswana-Mafuya, N., & Peltzer, K. (2018). Racial or ethnic health disparities among older adults in four population groups in South Africa. *Annals of Global Health*, *84*(1), 7–14. https://doi.org/10.29024/aogh.13

Phaswana-Mafuya, N., Peltzer, K., Chirinda, W., Musekiwa, A., & Kose, Z. (2013). Sociodemographic predictors of multiple non-communicable disease risk factors among older adults in South Africa. *Global Health Action*, *6*(1). https://doi.org/10.3402/gha.v6i0.20680

Phaswana-Mafuya, N., Peltzer, K., Chirinda, W., Musekiwa, A., Kose, Z., Hoosain, E., Davids, A., & Ramlagan, S. (2013). Self-reported prevalence of chronic non-communicable diseases and associated factors among older adults in south africa. *Global Health Action*, *6*(1). https://doi.org/10.3402/gha.v6i0.20936

Phillips-Howard, P. A., Laserson, K. F., Amek, N., Beynon, C. M., Angell, S. Y., Khagayi, S., Byass, P., Hamel, M. J., Van Eijk, A. M., Zielinski-Gutierrez, E., Slutsker, L., De Cock, K. M., Vulule, J., & Odhiambo, F. O. (2014). Deaths ascribed to non-communicable diseases among rural kenyan adults are proportionately increasing: Evidence from a health and demographic surveillance system, 2003-2010. *PLoS ONE*, *9*(11). https://doi.org/10.1371/journal.pone.0114010

Phukubye, P., & Oyedele, O. (2011). The incidence and structure of the fabella in a South African cadaver sample. *Clinical Anatomy*, *24*(1), 84–90. https://doi.org/10.1002/ca.21049

Pienaar, E., Stearn, N., & Swanepoel, D. W. (2010). Self-reported outcomes of aural rehabilitation for adult hearing aid users in a South African context. *The South African Journal of Communication Disorders. Die Suid-Afrikaanse Tydskrif Vir Kommunikasieafwykings*, *57*. https://doi.org/10.4102/sajcd.v57i1.44

Pieterse, S., Manandhar, M., & Ismail, S. (2002). The association between nutritional status and handgrip strength in older Rwandan refugees. *European Journal of Clinical Nutrition*, *56*(10), 933–939. https://doi.org/10.1038/sj.ejcn.1601443

Pilleron, S., Clément, J. P., Ndamba-Bandzouzi, B., Mbelesso, P., Dartigues, J. F., Preux, P. M., Guerchet, M., Désormais, I., Lacroix, P., Aboyans, V., Desport, J. C., Jésus, P., Tchalla, A. E., Marin, B., & Lambert, J. C. (2015). Is dependent personality disorder associated with mild cognitive impairment

and dementia in Central Africa? A result from the EPIDEMCA programme. *International Psychogeriatrics*, *27*(2), 279–288. https://doi.org/10.1017/S104161021400180X

Pilleron, Sophie, Aboyans, V., Desormais, I., Lacroix, P., Preux, P. M., Guerchet, M., Mbelesso, P., Ndamba-Bandzouzi, B., Desport, J. C., Jésus, P., Tchalla, A. E., Marin, B., Clément, J. P., Lambert, J. C., & Dartigues, J. F. (2017). Prevalence, awareness, treatment, and control of hypertension in older people in Central Africa: the EPIDEMCA study. *Journal of the American Society of Hypertension*, *11*(7), 449–460. https://doi.org/10.1016/j.jash.2017.04.013

Pilleron, Sophie, Guerchet, M., Ndamba-Bandzouzi, B., Mbelesso, P., Dartigues, J.-F., Preux, P.-M., & Cl�ment, J.-P. (2015). Association between Stressful Life Events and Cognitive Disorders in Central Africa: Results from the EPIDEMCA Program. *Neuroepidemiology*, *44*(2), 99–107. https://doi.org/10.1159/000375462

Pilleron, Sophie, Jésus, P., Desport, J. C., Mbelesso, P., Ndamba-Bandzouzi, B., Clément, J. P., Dartigues, J. F., Preux, P. M., & Guerchet, M. (2015). Association between mild cognitive impairment and dementia and undernutrition among elderly people in Central Africa: Some results from the EPIDEMCA (Epidemiology of Dementia in Central Africa) programme. *British Journal of Nutrition*, *114*(2), 306–315. https://doi.org/10.1017/S0007114515001749

Pilleron, Sophie, Soerjomataram, I., Charvat, H., Chokunonga, E., Somdyala, N. I. M., Wabinga, H., Korir, A., Bray, F., Jemal, A., & Maxwell Parkin, D. (2019). Cancer incidence in older adults in selected regions of sub-Saharan Africa, 2008–2012. *International Journal of Cancer*, *144*(8), 1824–1833. https://doi.org/10.1002/ijc.31880

Preux, P. M., & Desport, J. C. (2014). The nutritional status of older people with and without dementia living in an urban setting in Central Africa: The EDAC study. *Journal of Nutrition, Health and Aging*, *18*(10), 868–875. https://doi.org/10.1007/s12603-014-0483-7

Prinsloo, F. R. (1991). *Health services - Needs of the elderly in two black urban areas of the Cape Peninsula*. South African Medical Journal. https://pubmed.ncbi.nlm.nih.gov/2020892/

Puckree, T., & Uthum, P. (2014). Effectiveness of a community based programme of physiotherapy on stability, balance and function of stroke patients. *South African Journal of Physiotherapy*, *70*(1), 3–8. https://go.gale.com/ps/i.do?p=HRCA&sw=w&issn=03796175&v=2.1&it=r&id=GALE%7CA455784087&sid=googleScholar&linkaccess=fulltext

Puckree, T., Chetty, T. P., Ramlakan, S., Simelane, T. V. S., & Lin, J. (1997). An evaluation of the functional status of the residents of a geriatric residential facility in South Africa. *Disability and Rehabilitation*, *19*(12), 552–555. https://doi.org/10.3109/09638289709166049

Puckree, Threethambal, Mkhize, M., Mgobhozi, Z., & Lin, J. (2002). African traditional healers: What health care professionals need to know. *International Journal of Rehabilitation Research*, *25*(4), 247–251. https://doi.org/10.1097/00004356-200212000-00001

Pupwe, G., Ngalamika, O., & Akudugu, J. (2020). Chemotherapy for elderly colorectal cancer patients at a tertiary hospital in South Africa. *The Pan African Medical Journal*, *37*.

Putnam, H. W. I., Jones, R., Rogathi, J., Gray, W. K., Swai, B., Dewhurst, M., Dewhurst, F., & Walker, R. W. (2018). Hypertension in a resource-limited setting: Is it associated with end organ damage in older adults in rural Tanzania? *The Journal of Clinical Hypertension*, *20*(2), 217–224. https://doi.org/10.1111/jch.13187

Raal, F. J., Blom, D. J., Naidoo, S., Bramlage, P., & Brudi, P. (2013). Prevalence of dyslipidaemia in statin-treated patients in South Africa: Results of the DYSlipidaemia International Study (DYSIS). *Cardiovascular Journal of Africa*, *24*(8), 330–338. https://doi.org/10.5830/CVJA-2013-071

Raal, F., Schamroth, C., Blom, D., Marx, J., Rajput, M., Haus, M., Hussain, R., Cassim, F., Nortjé, M., Vandenhoven, G., & Temmerman, A. M. (2011). CEPHEUS SA: A South African survey on the undertreatment of hypercholesterolaemia. In *Cardiovascular Journal of Africa* (Vol. 22, Issue 5, pp. 234–240). Clinics Cardive Publishing (Pty) Ltd. https://doi.org/10.5830/CVJA-2011-044

Rabie, T., Klopper, H. C., & Watson, M. J. (2016). Relation of socio-economic status to the independent application of self-care in older persons of South Africa. *Health SA Gesondheid*, *21*, 155–161. https://doi.org/10.1016/j.hsag.2015.02.007

Rajak, S. N., Mohamed, M. D., & Pelosini, L. (2009). Further insight into west african crystalline maculopathy. *Archives of Ophthalmology*, *127*(7), 863–868. https://doi.org/10.1001/archophthalmol.2009.129

Ralston, M. (2018). The Role of Older Persons' Environment in Aging Well: Quality of Life, Illness, and Community Context in South Africa. *Gerontologist*, *58*(1), 111–120. https://doi.org/10.1093/geront/gnx091

Ralston, M., Schatz, E., Menken, J., Gómez-Olivé, F. X., & Tollman, S. (2015). Who benefits—or does not—from south africa's old age pension? Evidence from characteristics of rural pensioners and non-pensioners. *International Journal of Environmental Research and Public Health*, *13*(1).

https://doi.org/10.3390/ijerph13010085

Ralston, M., Schatz, E., Menken, J., Gómez-Olivé, F. X., & Tollman, S. (2019). Policy Shift: South Africa's Old Age Pensions' Influence on Perceived Quality of Life. *Journal of Aging and Social Policy*, *31*(2), 138–154. https://doi.org/10.1080/08959420.2018.1542243

Ramjeeth, A., Butkow, N., Raal, F. J., & Maholwana-Mokgatlhe, M. (2008). The evaluation of low-density lipoprotein cholesterol goals achieved in patients with established cardiovascular disease and/or hyperlipidaemia receiving lipid-lowering therapy: the South African Not at Goal study (SA-NAG). *Cardiovascular Journal of Africa*, *19*(2), 88–94. http://www.ncbi.nlm.nih.gov/pubmed/18516354

Ramlagan, S., Peltzer, K., & Phaswana-Mafuya, N. (2013). Social capital and health among older adults in South Africa. *BMC Geriatrics*, *13*(1), 100. https://doi.org/10.1186/1471-2318-13-100

Ramlagan, S., Peltzer, K., & Phaswana-Mafuya, N. (2014). Hand grip strength and associated factors in non-institutionalised men and women 50 years and older in South Africa. *BMC Research Notes*, *7*(1), 8. https://doi.org/10.1186/1756-0500-7-8

Ramlall, S., Chipps, J., Bhigjee, A. I., & Pillay, B. J. (2014). Sensitivity and specificity of neuropsychological tests for dementia and mild cognitive impairment in a sample of residential elderly in South Africa. *South African Journal of Psychiatry*, *20*(4), 153–159. https://doi.org/10.7196/SAJP.558

Ramocha, L. M., Louw, Q. A., & Tshabalala, M. D. (2017). Quality of life and physical activity among older adults living in institutions compared to the community. *South African Journal of Physiotherapy*, *73*(1). https://doi.org/10.4102/sajp.v73i1.342

Rand, D., & Eng, J. J. (2015). Predicting daily use of the affected upper extremity 1 year after stroke. *Journal of Stroke and Cerebrovascular Diseases*, *24*(2), 274–283. https://doi.org/10.1016/j.jstrokecerebrovasdis.2014.07.039

Randall, S. (2016). The quality of demographic data on older Africans. *DEMOGRAPHIC RESEARCH*, *34*. https://doi.org/10.4054/DemRes.2016.34.5

Randall, S., & Coast, E. (2016). The quality of demographic data on older Africans. *Demographic Research*, *34*(1), 143–174. https://doi.org/10.4054/DemRes.2016.34.5

Rangel, E. L., Cooper, Z., Olufajo, O. A., Reznor, G., Lipsitz, S. R., Salim, A., Kwakye, G., Calahan, C., Sarhan, M., & Hanna, J. S. (2015). Mortality after emergency surgery continues to rise after discharge in the elderly. *Journal of Trauma and Acute Care Surgery*, *79*(3), 349–358.

https://doi.org/10.1097/TA.0000000000000773

Ranjith, N., Myeni, N. N., Sartorius, B., & Mayise, C. (2017). Association between Hyperuricemia and Major Adverse Cardiac Events in Patients with Acute Myocardial Infarction. *Metabolic Syndrome and Related Disorders*, *15*(1), 18–25. https://doi.org/10.1089/met.2016.0032

Rayner, B., Ramesar, R., Steyn, K., Levitt, N., Lombard, C., & Charlton, K. (2012). G-protein-coupled receptor kinase 4 polymorphisms predict blood pressure response to dietary modification in Black patients with mild-to-moderate hypertension. *Journal of Human Hypertension*, *26*(5), 334–339. https://doi.org/10.1038/jhh.2011.33

Rayner, B., Ramesar, R., Steyn, K., Levitt, N., Lombard, C., & Charlton, K. (2012). G-protein-coupled receptor kinase 4 polymorphisms predict blood pressure response to dietary modification in Black patients with mild-to-moderate hypertension. *Journal of Human Hypertension*, *26*(5), 334–339. https://doi.org/10.1038/jhh.2011.33

Rayner, Brian, Blockman, M., Baines, D., & Trinder, Y. (2007). *A survey of hypertensive practices at two community health centres in Cape Town*. South African Medical Journal. https://doi.org/10.7196/SAMJ.789

Reddy, J., Parker, J. R., Africa, C. W., & Stephen, L. X. G. (1985). Prevalence and severity of periodontitis in a high fluoride area in South Africa. *Community Dentistry and Oral Epidemiology*, *13*(2), 108–112. https://doi.org/10.1111/j.1600-0528.1985.tb01688.x

Reiger, S., Jardim, T. V., Abrahams-Gessel, S., Crowther, N. J., Wade, A., Gomez-Olive, F. X., Salomon, J., Tollman, S., & Gaziano, T. A. (2017). Awareness, treatment, and control of dyslipidemia in rural South Africa: The HAALSI (Health and Aging in Africa: A Longitudinal Study of an INDEPTH Community in South Africa) study. *PLoS ONE*, *12*(10). https://doi.org/10.1371/journal.pone.0187347

Resnikoff, S. (1988). *Bietti's keratopathy. A study of risk factors in Central Africa*. Journal Francais d'Ophtalmologie. https://pubmed.ncbi.nlm.nih.gov/3266940/

Rhoda, A., Cunningham, N., Azaria, S., & Urimubenshi, G. (2015). Provision of inpatient rehabilitation and challenges experienced with participation post discharge: Quantitative and qualitative inquiry of African stroke patients. In *BMC Health Services Research* (Vol. 15, Issue 1). BioMed Central Ltd. https://doi.org/10.1186/s12913-015-1057-z

Richards, E., Zalwango, F., Seeley, J., Scholten, F., & Theobald, S. (2013). Neglected older women and men: Exploring age and gender as structural drivers

of HIV among people aged over 60 in Uganda. *African Journal of AIDS Research*, *12*(2), 71–78. https://doi.org/10.2989/16085906.2013.831361

Rishworth, A., Elliott, S. J., & Kangmennaang, J. (2020). Getting old well in sub saharan Africa: Exploring the social and structural drivers of subjective wellbeing among elderly men and women in Uganda. *International journal of environmental research and public health*, *17*(7), 2347.

Robb, L., Walsh, C. M., Nel, M., Nel, A., Odendaal, H., & van Aardt, R. (2017). Malnutrition in the elderly residing in long-term care facilities: a cross sectional survey using the Mini Nutritional Assessment (MNA®) screening tool. *South African Journal of Clinical Nutrition*, *30*(2), 34–40. https://doi.org/10.1080/16070658.2016.1248062

Rodriguez, H., Brathwaite, D., & Dorsey, S. (2002). *Depression and social support in the elderly population: a study of rural South African elders.* The ABNF Journal : Official Journal of the Association of Black Nursing Faculty in Higher Education, Inc. https://pubmed.ncbi.nlm.nih.gov/12017113/

Rohr, J. K., Xavier Gómez-Olivé, F., Rosenberg, M., Manne-Goehler, J., Geldsetzer, P., Wagner, R. G., Houle, B., Salomon, J. A., Kahn, K., Tollman, S., Berkman, L., & Bärnighausen, T. (2017). Performance of self-reported HIV status in determining true HIV status among older adults in rural South Africa: A validation study: A. *Journal of the International AIDS Society*, *20*(1). https://doi.org/10.7448/IAS.20.1.21691

Roos, V., & Klopper, H. (2010). Older Persons' Experiences of Loneliness: A South African Perspective. *Journal of Psychology in Africa*, *20*(2), 281–289. https://doi.org/10.1080/14330237.2010.10820377

Roos, V., & Malan, L. (2012). The role of context and the interpersonal experience of loneliness among older people in a residential care facility. *Global Health Action*, *5*(1), 18861. https://doi.org/10.3402/gha.v5i0.18861

Roos, V., & Wheeler, A. (2016). Older people's experiences of giving and receiving empathy in relation to middle adolescents in rural South Africa. *South African Journal of Psychology*, *46*(4), 517–529. https://doi.org/10.1177/0081246316638563

Roos, V., Kolobe, P. S., & Keating, N. (2014). (Re)creating community: Experiences of Older Women Forcibly Relocated During Apartheid. *Journal of Community & Applied Social Psychology*, *24*(1), 12–25. https://doi.org/10.1002/casp.2177

Roos, V., Silvestre, S., & De Jager, T. (2017). Intergenerational Care Perceptions of Older Women and Middle Adolescents in a Resource-Constrained Community in South Africa. *Journal of Gerontological Social Work*, *60*(2), 104–119. https://doi.org/10.1080/01634372.2016.1268231

Rosenberg, M., Gómez-Olivé, F. X., Wagner, R. G., Rohr, J., Payne, C. F., Berkman, L., ... & Kobayashi, L. C. (2020). The relationships between cognitive

function, literacy and HIV status knowledge among older adults in rural South Africa. *Journal of the International AIDS Society*, *23*(3), e25457.

Rossouw, L., & Smith, A. (2017). A comparable yardstick: Adjusting for education bias in South African health system responsiveness ratings. *Health Policy and Planning*, *32*(suppl_3), iii67–iii74. https://doi.org/10.1093/heapol/czx107

Rotchford, A. P., & Johnson, G. J. (2000). *Rapid assessment of cataract surgical coverage in rural Zululand*. South African Medical Journal. https://pubmed.ncbi.nlm.nih.gov/11081113/

Rotchford, A. P., Rotchford, K. M., Mthethwa, L. P., & Johnson, G. J. (2002). Reasons for poor cataract surgery uptake - A qualitative study in rural south africa. *Tropical Medicine and International Health*, *7*(3), 288–292. https://doi.org/10.1046/j.1365-3156.2002.00850.x

Rotchford, Alan P., & Johnson, G. J. (2002). Glaucoma in Zulus: A population-based cross-sectional survey in a rural district in South Africa. *Archives of Ophthalmology*, *120*(4), 471–478. https://doi.org/10.1001/archopht.120.4.471

Rotchford, Alan P., Kirwan, J. F., Johnson, G. J., & Roux, P. (2003). Exfoliation syndrome in black South Africans. *Archives of Ophthalmology*, *121*(6), 863–870. https://doi.org/10.1001/archopht.121.6.863

Rotchford, Alan P., Kirwan, J. F., Muller, M. A., Johnson, G. J., & Roux, P. (2003). Temba glaucoma study: A population-based cross-sectional survey in urban South Africa. *Ophthalmology*, *110*(2), 376–382. https://doi.org/10.1016/S0161-6420(02)01568-3

Rutagumirwa, S. K., & Bailey, A. (2019). "I Have to Listen to This Old Body": Femininity and the Aging Body. *Gerontologist*, *59*(2), 368–377. https://doi.org/10.1093/geront/gnx161

Saeed, B. I. I., Yawson, A. E., Nguah, S., Agyei-Baffour, P., Emmanuel, N., & Ayesu, E. (2016). Effect of socio-economic factors in utilization of different healthcare services among older adult men and women in Ghana. *BMC Health Services Research*, *16*(1), 390. https://doi.org/10.1186/s12913-016-1661-6

Sanuade, O. A., Dodoo, F. N.-A., Koram, K., & de-Graft Aikins, A. (2019). Prevalence and correlates of stroke among older adults in Ghana: Evidence from the Study on Global AGEing and adult health (SAGE). *PLOS ONE*, *14*(3), e0212623. https://doi.org/10.1371/journal.pone.0212623

Sanya, E. O., Abiodun, A. A., Kolo, P. O., Olanrewaju, T., & Adekeye, K. (2011). Profile and causes of mortality among elderly patients seen in a tertiary care hospital in Nigeria. *Annals of African Medicine*, *10*(4), 278–283. https://doi.org/10.4103/1596-3519.87043

Sarfo, F. S., Akassi, J., Agyei, M., Kontoh, S., & Ovbiagele, B. (2020). Risk Factor Control in Stroke Survivors with Diagnosed and Undiagnosed Diabetes: A Ghanaian Registry Analysis. *Journal of Stroke and Cerebrovascular Diseases*, *29*(12), 105304.

Sarkodie, B. D., Botwe, B. O., & Brakohiapa, E. K. K. (2020). Percutaneous transhepatic biliary stent placement in the palliative management of malignant obstructive jaundice: initial experience in a tertiary center in Ghana. *The Pan African Medical Journal*, *37*.

Schatz, E. J. (2007). “Taking care of my own blood”: Older women’s relationships to their households in rural South Africa. *Scandinavian Journal of Public Health*, *35*(SUPPL. 69), 147–154. https://doi.org/10.1080/14034950701355676

Schatz, E. J. (2009). Reframing vulnerability: Mozambican refugees’ access to state-funded pensions in rural South Africa. *Journal of Cross-Cultural Gerontology*, *24*(3), 241–258. https://doi.org/10.1007/s10823-008-9089-z

Schatz, E., & Gilbert, L. (2014). “My Legs Affect Me a Lot.... I Can No Longer Walk to the Forest to Fetch Firewood”: Challenges Related to Health and the Performance of Daily Tasks for Older Women in a High HIV Context. *Health Care for Women International*, *35*(7–9), 771–788. https://doi.org/10.1080/07399332.2014.900064

Schatz, E., & Knight, L. (2018). “I was referred from the other side”: Gender and HIV testing among older South Africans living with HIV. *PLOS ONE*, *13*(4), e0196158. https://doi.org/10.1371/journal.pone.0196158

Schatz, E., Gómez-Olivé, X., Ralston, M., Menken, J., & Tollman, S. (2012). The impact of pensions on health and wellbeing in rural South Africa: Does gender matter? *Social Science and Medicine*, *75*(10), 1864–1873. https://doi.org/10.1016/j.socscimed.2012.07.004

Schatz, E., Madhavan, S., Collinson, M., Gómez-Olivé, F. X., & Ralston, M. (2015). Dependent or Productive? A New Approach to Understanding the Social Positioning of Older South Africans Through Living Arrangements. *Research on Aging*, *37*(6), 581–605. https://doi.org/10.1177/0164027514545976

Schatz, E., Ralston, M., Madhavan, S., Collinson, M. A., & Gómez-Olivé, F. X. (2018). Living arrangements, disability and gender of older adults among rural South Africa. *Journals of Gerontology - Series B Psychological Sciences and Social Sciences*, *73*(6), 1112–1122. https://doi.org/10.1093/geronb/gbx081

Schatz, E., Seeley, J., Negin, J., Weiss, H. A., Tumwekwase, G., Kabunga, E., Nalubega, P., & Mugisha, J. (2019). “for us here, we remind ourselves”: Strategies and barriers to ART access and adherence among older Ugandans. *BMC Public Health*, *19*(1), 131. https://doi.org/10.1186/s12889-019-6463-4

Schmidlin, E. J., Steyn, M., Houlton, T. M. R., & Briers, N. (2018). Facial ageing in South African adult males. *Forensic Science International*, *289*, 277–286. https://doi.org/10.1016/j.forsciint.2018.05.006

Schnaid, E., MacPhail, A. P., & Sweet, M. B. E. (2000). Fractured neck of femur in black patients. *Journal of Bone and Joint Surgery - Series B*, *82*(6), 872–875. https://doi.org/10.1302/0301-620X.82B6.10589

Schoeman, B. J. (1996). *[Squamous cell carcinoma in neuropathic plantar ulcers in leprosy: another example of Marjolin's ulcer]*. S Afr Med J. https://pubmed.ncbi.nlm.nih.gov/8966648/

Scholten, F., Mugisha, J., Seeley, J., Kinyanda, E., Nakubukwa, S., Kowal, P., Naidoo, N., Boerma, T., Chatterji, S., & Grosskurth, H. (2011). Health and functional status among older people with HIV/AIDS in Uganda. *BMC Public Health*, *11*(1), 886. https://doi.org/10.1186/1471-2458-11-886

Segal, I., Reinach, S. G., & De Beer, M. (1988). Factors associated with oesophageal cancer in soweto, south africa. *British Journal of Cancer*, *58*(5), 681–686. https://doi.org/10.1038/bjc.1988.286

Segal, Isidor, & Walker, A. R. P. (1982). Diverticular Disease in Urban Africans in South Africa. *Digestion*, *24*(1), 42–46. https://doi.org/10.1159/000198773

Segal, Isidor, Solomon, A., Tim, L. O., Rabin, M., & Walker, A. R. P. (1980). Hiatus hernia in Johannesburg blacks. *Undefined*.

Semeere, A. S., Lwanga, I., Sempa, J., Parikh, S., Nakasujja, N., Cumming, R., Kambugu, A., & Mayanja-Kizza, H. (2014). Mortality and immunological recovery among older adults on antiretroviral therapy at a Large Urban HIV clinic in Kampala, Uganda. *Journal of Acquired Immune Deficiency Syndromes*, *67*(4), 382–389. https://doi.org/10.1097/QAI.0000000000000330

Simo, N., Kuate-Tegueu, C., Ngankou-Tchankeu, S., Doumbe, J., Maiga, Y., Cesari, M., ... & Tabue-Teguo, M. (2020). Correlates of diabetic polyneuropathy of the elderly in Sub-Saharan Africa. *PloS one*, *15*(10), e0240602.

Sidloyi, S. S., & Bomela, N. J. (2016). Survival strategies of elderly women in Ngangelizwe Township, Mthatha, South Africa: Livelihoods, social networks and income. *Archives of Gerontology and Geriatrics*, *62*, 43–52. https://doi.org/10.1016/j.archger.2015.09.006

Silbert, M. V. (1977). *Medical and psychosocial problems of the aged*. South African Medical Journal. https://pubmed.ncbi.nlm.nih.gov/857322/

Singo, V. J., Lebese, R. T., Maluleke, T. X., & Nemathaga, L. H. (2015). The views of the elderly on the impact that HIV and AIDS has on their lives in the

Thulamela Municipality, Vhembe District, Limpopo province. *Curationis*, *38*(1). https://doi.org/10.4102/curationis.v38i1.1166

Sissolak, G., Wood, L., Smith, L., Chan, J. (Wing C., Armitage, J., & Jacobs, P. (2013). Tissue microarray in a subset of South African patients with DLBCL. *Transfusion and Apheresis Science*, *49*(2), 120–132. https://doi.org/10.1016/j.transci.2013.07.013

Skidmore, E. R., Whyte, E. M., Butters, M. A., Terhorst, L., & Reynolds, C. F. (2015). Strategy Training During Inpatient Rehabilitation May Prevent Apathy Symptoms After Acute Stroke. *PM and R*, *7*(6), 562–570. https://doi.org/10.1016/j.pmrj.2014.12.010

Skovdal, M., Campbell, C., Madanhire, C., Nyamukapa, C., & Gregson, S. (2011). Challenges faced by elderly guardians in sustaining the adherence to antiretroviral therapy in HIV-infected children in Zimbabwe. In *AIDS Care - Psychological and Socio-Medical Aspects of AIDS/HIV* (Vol. 23, Issue 8, pp. 957–964). AIDS Care. https://doi.org/10.1080/09540121.2010.542298

Sliwa, K., Carrington, M. J., Klug, E., Opie, L., Lee, G., Ball, J., & Stewart, S. (2010). Predisposing factors and incidence of newly diagnosed atrial fibrillation in an urban African community: Insights from the Heart of Soweto Study. *Heart*, *96*(23), 1878–1882. https://doi.org/10.1136/hrt.2010.206938

Smith, A. J., Hall, D. R., & Grové, D. (2005). Current patient perceptions on the menopause: A South African perspective. In *Climacteric* (Vol. 8, Issue 4, pp. 327–332). Taylor & Francis. https://doi.org/10.1080/13697130500196817

Sobnach, S., Khosa, S. A., Pather, S., Longhurst, S., Kahn, D., & Raubenheimer, P. J. (2009). First case report of pharyngeal cysticercosis. *Transactions of the Royal Society of Tropical Medicine and Hygiene*, *103*(2), 206–208. https://doi.org/10.1016/j.trstmh.2008.08.017

Solomon, A., Christian, B. F., Woodiwiss, A., Norton, G., & Dessein, P. (2011). Burden of depressive symptoms in South African public healthcare patients with established rheumatoid arthritis: a case-control study. *Undefined*.

Solomon, Ahmed, Christian, B. F., Dessein, P. H., & Stanwix, A. E. (2005). The need for tighter rheumatoid arthritis control in a South African public health care center. *Seminars in Arthritis and Rheumatism*, *35*(2), 122–131. https://doi.org/10.1016/j.semarthrit.2005.05.005

Solomon, L., Schnitzler, C. M., & Browett, J. P. (1982). Osteoarthritis of the hip: The patient behind the disease. *Annals of the Rheumatic Diseases*, *41*(2), 118–125. https://doi.org/10.1136/ard.41.2.118

Solomons, K. (1984). *Malignant mesothelioma - clinical epidemiological features. A report of 80 cases*. South African Medical Journal.

http://www.embase.com/search/results?subaction=viewrecord&from=export&id=L14014232

Somdyala, N. I., Bradshaw, D., Gelderblom, W. C., & Parkin, D. M. (2010). Cancer incidence in a rural population of South Africa, 1998-2002. *International Journal of Cancer*, *127*(10), 2420–2429. https://doi.org/10.1002/ijc.25246

Ssengonzi, R. (2007). The Plight of Older Persons as Caregivers to People Infected/Affected by HIV/AIDS: Evidence from Uganda. *Journal of Cross-Cultural Gerontology*, *22*(4), 339–353. https://doi.org/10.1007/s10823-007-9043-5

Ssonko, M., Stanaway, F., Mayanja, H. K., Namuleme, T., Cumming, R., Kyalimpa, J. L., Karamagi, Y., Mukasa, B., & Naganathan, V. (2018). Polypharmacy among HIV positive older adults on anti-retroviral therapy attending an urban clinic in Uganda. *BMC Geriatrics*, *18*(1), 125. https://doi.org/10.1186/s12877-018-0817-0

Surka, J., & Hussain, S. (2001). *Outcome of high-volume cataract surgery at an academic hospital*. South African Medical Journal. https://pubmed.ncbi.nlm.nih.gov/11680328/

Swart, L., Dreyer, W., van Zyl, P., & Blignaut, R. (2014). Early Loading of Mandibular Implants Placed Immediately After Extraction: A 10-year Prospective Study of Eight Patients. *The International Journal of Oral & Maxillofacial Implants*, *29*(6), 1388–1396. https://doi.org/10.11607/jomi.3670

Tannor, E. K., Archer, E., Kapembwa, K., Van Schalkwyk, S. C., & Davids, M. R. (2017). Quality of life in patients on chronic dialysis in South Africa: A comparative mixed methods study. *BMC Nephrology*, *18*(1). https://doi.org/10.1186/s12882-016-0425-1

Tanyi, P. L., Pelser, A., & Okeibunor, J. (2018). Hiv/aids and older adults in cameroon: Emerging issues and implications for caregiving and policy-making. *Sahara J*, *15*(1), 7–19. https://doi.org/10.1080/17290376.2018.1433059

Tarekegne, F. E., Padyab, M., Schröders, J., & Williams, J. S. (2018). Sociodemographic and behavioral characteristics associated with selfreported diagnosed diabetes mellitus in adults aged 50+ years in Ghana and South Africa: Results from the WHOSAGE wave 1. *BMJ Open Diabetes Research and Care*, *6*(1), 449. https://doi.org/10.1136/bmjdrc-2017-000449

Till, H., & Till, G. (1999). *Experience in a Hospital-Based Clinic as Part of Chiropractic Undergraduate Training*. The Journal of Chiropractic Education. https://www.researchgate.net/publication/230601837_Experience_in_a_hospital-based_clinic_as_part_of_chiropractic_undergraduate_training

Tipping, B., Kalula, S., & Badri, M. (2006). The burden and risk factors for adverse drug events in older patients - A prospective cross-sectional study. *South African Medical Journal*, *96*(12 I), 1255–1259. https://doi.org/10.7196/SAMJ.1359

Togonu-Bickersteth, F. (1986). Age identification among Yoruba aged. *Journals of Gerontology*, *41*(1), 110–113. https://doi.org/10.1093/geronj/41.1.110

Tolani, M. A., Suleiman, A., Awaisu, M., Abdulaziz, M. M., Lawal, A. T., & Bello, A. (2020). Acute urinary tract infection in patients with underlying benign prostatic hyperplasia and prostate cancer. *The Pan African Medical Journal*, *36*.

Tomás, J. M., Gutiérrez, M., Sancho, P., & Galiana, L. (2012). Predicting perceived health in Angolan elderly: The moderator effect of being oldest old. *Archives of Gerontology and Geriatrics*, *55*(3), 605–610. https://doi.org/10.1016/j.archger.2012.06.010

Tomita, A., & Burns, J. K. (2013). Depression, disability and functional status among community-dwelling older adults in South Africa: Evidence from the first South African National Income Dynamics Study. *International Journal of Geriatric Psychiatry*, *28*(12), 1270–1279. https://doi.org/10.1002/gps.3954

Torgersen, J., Bellamy, S. L., Ratshaa, B., Han, X., Mosepele, M., Zuppa, A. F., Vujkovic, M., Steenhoff, A. P., Bisson, G. P., & Gross, R. (2019). Impact of Efavirenz Metabolism on Loss to Care in Older HIV+ Africans. *European Journal of Drug Metabolism and Pharmacokinetics*, *44*(2), 179–187. https://doi.org/10.1007/s13318-018-0507-5

Toure, K., Coume, M., Ndiaye, M., Zunzunegui, M. V., Bacher, Y., Diop, A. G., & Ndiaye, M. M. (2012). Risk Factors for Dementia in a Senegalese Elderly Population Aged 65 Years and Over. *Dementia and Geriatric Cognitive Disorders Extra*, *2*(1), 160–168. https://doi.org/10.1159/000332022

Udjo, E. O. (2006). Demographic impact of HIV/AIDS on the young and elderly populations in South Africa. *Journal of Intergenerational Relationships*, *4*(2), 23–41. https://doi.org/10.1300/J194v04n02_03

Udvardy, M., & Cattell, M. (1992). Gender, aging and power in sub-Saharan Africa: Challenges and puzzles. *Journal of Cross-Cultural Gerontology*, *7*(4), 275–288. https://doi.org/10.1007/BF01848695

Uwakwe, R., Ibeh, C. C., Modebe, A. I., Bo, E., Ezeama, N., Njelita, I., Ferri, C. P., & Prince, M. J. (2009). The epidemiology of dependence in older people in Nigeria: Prevalence, determinants, informal care, and health service utilization. A 10/66 Dementia Research Group cross-sectional survey. *Journal of the American Geriatrics Society*, *57*(9), 1620–1627. https://doi.org/10.1111/j.1532-5415.2009.02397.x

Uys, L. R., & Hunt, B. N. (1990). Standards for the nursing care of the frail aged. *Curationis*, *13*(1–2), 19–20. https://doi.org/10.4102/curationis.v13i1/2.277

Van Biljon, L., Nel, P., & Roos, V. (2015). A partial validation of the WHOQOL-OLD in a sample of older people in South Africa. *Global Health Action*, *8*(1), 28209. https://doi.org/10.3402/gha.v8.28209

van Biljon, L., Roos, V., & Botha, K. (2015). A Conceptual Model of Quality of Life for Older People in Residential Care Facilities in South Africa. *Applied Research in Quality of Life*, *10*(3), 435–457. https://doi.org/10.1007/s11482-014-9322-6

Van Der Geest, S. (2004). “They don’t come to listen”: the experience of loneliness among older people in Kwahu, Ghana. *Journal of Cross-Cultural Gerontology*, *19*(2), 77–96. https://doi.org/10.1023/B:JCCG.0000027846.67305.f0

van der Pas, S., Ramklass, S., O’Leary, B., Anderson, S., Keating, N., & Cassim, B. (2015). Features of home and neighbourhood and the liveability of older South Africans. *European Journal of Ageing*, *12*(3), 215–227. https://doi.org/10.1007/s10433-015-0343-2

Van Der Wielen, N., Channon, A. A., & Falkingham, J. (2018). Universal health coverage in the context of population ageing: What determines health insurance enrolment in rural Ghana? *BMC Public Health*, *18*(1), 657. https://doi.org/10.1186/s12889-018-5534-2

Van Dongen, E. (2003). “Die lewe vat ek net soos ek dit kry.” Life stories and remembrance of older coloured people on farms in the western cape province. *Journal of Cross-Cultural Gerontology*, *18*(4), 303–335. https://doi.org/10.1023/B:JCCG.0000004900.01896.ac

van Dongen, E. (2005). Remembering in times of misery: Can older people in South Africa “get through”? *Ageing and Society*, *25*(4), 525–541. https://doi.org/10.1017/S0144686X05003806

van Rensburg, A. J., Kotze, I., Lubbe, M. S., & Cockeran, M. (2017). An elderly, urban population: Their experiences and expectations of pharmaceutical services in community pharmacies. *Health SA Gesondheid*, *22*, 241–251. https://doi.org/10.1016/j.hsag.2016.12.002

Van Sjaak Geest, D. E. R. (2002). “I want to go!” How older people in Ghana look forward to death. *Ageing and Society*, *22*(1), 7–28. https://doi.org/10.1017/S0144686X02008541

Van Staden, A. M., & Weich, D. J. V. (2007). Profile of the geriatric patient hospitalised at Universitas Hospital, South Africa. *South African Family Practice*, *49*(2), 14. https://doi.org/10.1080/20786204.2007.10873508

van Vuuren, A., Rheeder, P., & Hak, E. (2009). Effectiveness of influenza vaccination in the elderly in South Africa. *Epidemiology and Infection*, *137*(7), 994–

1002. https://doi.org/10.1017/S0950268808001386

Vlantis, A. C., Gregor, R. T., Elliot, H., & Oudes, M. (2003). Conversion from a non-indwelling to a Provox®2 indwelling voice prosthesis for speech rehabilitation: Comparison of voice quality and patient preference. *Journal of Laryngology and Otology*, *117*(10), 815–820. https://doi.org/10.1258/002221503770716278

Von Klemperer, A., Bateman, K., Owen, J., & Bryer, A. (2014). Thrombolysis risk prediction: Applying the SITS-SICH and SEDAN scores in South African patients. *Cardiovascular Journal of Africa*, *25*(5), 224–227. https://doi.org/10.5830/CVJA-2014-043

Vorobiof, D. A., Rapoport, B. L., Chasen, M. R., Slabber, C., McMichael, G., Eek, R., & Mohammed, C. (2004). First line therapy with paclitaxel (Taxol®) and pegylated liposomal doxorubicin (Caelyx®) in patients with metastatic breast cancer: A multicentre phase II study. *Breast*, *13*(3), 219–226. https://doi.org/10.1016/j.breast.2004.01.006

Vorster, M., Modiselle, M., Ebenhan, T., Wagener, C., Sello, T., Zeevaart, J. R., Moshokwa, E., & Sathekge, M. M. (2015). *Fluorine-18-Fluoroethylcholine PET/CT in the detection of prostate cancer: A South African experience*. Hellenic Journal of Nuclear Medicine. https://pubmed.ncbi.nlm.nih.gov/25840573/

Wachira, B. W., & Tyler, M. D. (2015). Characterization of in-hospital cardiac arrest in adult patients at a tertiary hospital in Kenya. *African Journal of Emergency Medicine*, *5*(2), 70–74. https://doi.org/10.1016/j.afjem.2014.10.006

Walker, A. R., Walker, B. F., Funani, L. S., & Segal, I. (1989). *Risk factors and survival from colorectal cancer in black patients in Soweto, South Africa.* Tropical Gastroenterology : Official Journal of the Digestive Diseases Foundation. https://pubmed.ncbi.nlm.nih.gov/2626780/

Walker, A. R., Walker, B. F., Manetsi, B., Molefe, O., & Walker, J. A. (1991). Serum Albumin Levels in Elderly Rural Africans. *International Journal for Vitamin and Nutrition Research*, *61*(4), 339–345. https://jglobal.jst.go.jp/en/detail?JGLOBAL_ID=200902081288495701

Walker, A. R., Walker, B. F., Manetsi, B., Tsotetsi, N. G., & Walker, A. J. (1990). Obesity in black women in Soweto, South Africa: minimal effects on hypertension, hyperlipidaemia and hyperglycaemia. *Journal of the Royal Society of Health*, *110*(3), 101–103. https://doi.org/10.1177/146642409011000311

Walker, A. R.P., & Walker, B. F. (2005). Lung cancer in Africans in a South African city population in transition. *European Journal of Cancer Prevention*,

14(2), 187–189. https://doi.org/10.1097/00008469-200504000-00017

Walker, A. R.P., Walker, B. F., Isaacson, C., Doodha, M. I., & Segal, I. (1986). Survival of black men with prostatic cancer in Soweto, Johannesburg, South Africa. *Journal of Urology*, *135*(1), 58–59. https://doi.org/10.1016/S0022-5347(17)45516-9

Walker, A. R.P., Walker, B. F., Tsotetsi, N. G., Sebitso, C., Siwedi, D., & Walker, A. J. (1992). Case-control study of prostate cancer in black patients in soweto, south africa. *British Journal of Cancer*, *65*(3), 438–441. https://doi.org/10.1038/bjc.1992.89

Walker, Alexander R. P., Segal, I., Posner, R. D., Shein, H. M., Tsotetsi, N. G., & Walker, A. J. (1989). Prevalence of gallstones in elderly black women in Soweto, Johannesburg, as assessed by ultrasound. *Undefined*.

Wallrauch, C., Bärnighausen, T., & Newell, M. L. (2010). *HIV prevalence and incidence in people 50 years and older in rural South Africa*. South African Medical Journal. https://doi.org/10.7196/samj.4181

Wandera, Stephen O., Ntozi, J., & Kwagala, B. (2014). Prevalence and correlates of disability among older Ugandans: evidence from the Uganda National Household Survey. *Global Health Action*, *7*(1), 25686. https://doi.org/10.3402/gha.v7.25686

Wandera, Stephen Ojiambo, Golaz, V., Kwagala, B., & Ntozi, J. (2015). Factors associated with self-reported ill health among older Ugandans: A cross sectional study. *Archives of Gerontology and Geriatrics*, *61*(2), 231–239. https://doi.org/10.1016/j.archger.2015.05.006

Wandera, Stephen Ojiambo, Kwagala, B., & Ntozi, J. (2015). Prevalence and risk factors for self-reported non-communicable diseases among older Ugandans: a cross-sectional study. *Global Health Action*, *8*(1), 27923. https://doi.org/10.3402/gha.v8.27923

Ware, L. J., Charlton, K., Schutte, A. E., Cockeran, M., Naidoo, N., & Kowal, P. (2017). Associations between dietary salt, potassium and blood pressure in South African adults: WHO SAGE Wave 2 Salt & Tobacco. *Nutrition, Metabolism and Cardiovascular Diseases*, *27*(9), 784–791. https://doi.org/10.1016/j.numecd.2017.06.017

Wasserman, L. J., Apffelstaedt, J. P., & de V. Odendaal, J. (2007). Conservative management of breast cancer in the elderly in a developing country. *World Journal of Surgical Oncology*, *5*(1), 108. https://doi.org/10.1186/1477-7819-5-108

Wasserman, S., & Bryer, A. (2012). Early outcomes of thrombolysis for acute ischaemic stroke in a South African tertiary care centre. *South African Medical*

Journal, *102*(6), 541–544. https://doi.org/10.7196/samj.5403

Wasserman, S., de Villiers, L., & Bryer, A. (2009). *Community-based care of stroke patients in a rural African setting*. South African Medical Journal. https://doi.org/10.7196/SAMJ.3284

Waterhouse, P., Van Der Wielen, N., Banda, P. C., & Channon, A. A. (2017). The impact of multi-morbidity on disability among older adults in South Africa: Do hypertension and socio-demographic characteristics matter? *International Journal for Equity in Health*, *16*(1). https://doi.org/10.1186/s12939-017-0537-7

Watkins, D., Brouwer, E., & Nugent, R. (2014). PT321 Economic consequences of cardiovascular disease in South African households: an analysis of the WHO Study on Global Aging (SAGE). *Global Heart*, *9*(1), e229. https://doi.org/10.1016/j.gheart.2014.03.2053

Watson, M. J., Klopper, H. C., & Kruger, A. (2013). Community-based collaboration in support of older persons. *Journal of Psychology in Africa*, *23*(3), 515–518. https://doi.org/10.1080/14330237.2013.10820661

Webb, E. M., Rheeder, P., & Van Zyl, D. G. (2015). Diabetes care and complications in primary care in the Tshwane district of South Africa. *Primary Care Diabetes*, *9*(2), 147–154. https://doi.org/10.1016/j.pcd.2014.05.002

Wentink, M. Q., Räkers, M., Stupart, D. A., Algar, U., Ramesar, R., & Goldberg, R. A. (2010). *Incidence and histological features of colorectal cancer in the Northern Cape province, South Africa*. South African Journal of Surgery. https://doi.org/10.7196/sajs.672

Werfalli, M., Kassanjee, R., Kalula, S., Kowal, P., Phaswana-Mafuya, N., & Levitt, N. S. (2018). Diabetes in South African older adults: prevalence and impact on quality of life and functional disability–as assessed using SAGE Wave 1 data. *Global Health Action*, *11*(1). https://doi.org/10.1080/16549716.2018.1449924

Wessels, P. F., & Riback, W. J. (2012). *DVT prophylaxis in relation to patient risk profiling - the TUNE-IN study*. South African Medical Journal. https://www.ajol.info/index.php/samj/article/view/74596

Westaway, M. S., Rheeder, P., & Gumede, T. (2001). The effect of type 2 diabetes mellitus on health-related quality of life (HRQOL). *Curationis*, *24*(1), 74–78. https://doi.org/10.4102/curationis.v24i1.805

Westaway, Margaret S. (2010a). *Effects of ageing, chronic disease and co-morbidity on the health and well-being of older residents of Greater Tshwane*. South African Medical Journal. https://doi.org/10.7196/SAMJ.3473

Westaway, Margaret S. (2010b). The impact of chronic diseases on the health and well-being of South Africans in early and later old age. *Archives of Gerontology and Geriatrics*, *50*(2), 213–221. https://doi.org/10.1016/j.archger.2009.03.012

Westaway, Margaret S., Jordaan, E. R., & Tsai, J. (2015). Investigating the Psychometric Properties of the Rosenberg Self-Esteem Scale for South African Residents of Greater Pretoria. *Evaluation and the Health Professions*, *38*(2), 181–199. https://doi.org/10.1177/0163278713504214

Westaway, Margaret S., Olorunju, S. A. S., & Rai, L.-C. J. (2007). Which Personal Quality of Life Domains Affect the Happiness of Older South Africans? In *Quality of Life Research* (Vol. 16, pp. 1425–1438). Springer. https://doi.org/10.2307/40212619

Whigham, B. T., Williams, S. E. I., Liu, Y., Rautenbach, R. M., Carmichael, T. R., Wheeler, J., Ziskind, A., Qin, X., Schmidt, S., Ramsay, M., Hauser, M. A., & Allingham, R. R. (2011). Myocilin mutations in black South Africans with POAG. *Molecular Vision*, *17*, 1064–1069. http://www.ncbi.nlm.nih.gov/pubmed/21552496

Whitelaw, D. A., Meyer, C. J., Bawa, S., & Jennings, K. (1994). *Post-discharge follow-up of stroke patients at Groote Schuur Hospital - A prospective study*. South African Medical Journal. https://www.ajol.info/index.php/samj/article/view/149020

Whitelaw, David A., Rayner, B. L., & Willcox, P. A. (1992). Community-Acquired Bacteremia in the Elderly: A Prospective Study of 121 Cases. *Journal of the American Geriatrics Society*, *40*(10), 996–1000. https://doi.org/10.1111/j.1532-5415.1992.tb04475.x

Whittaker, S., Prinsloo r., F., Wicht, C. L., & Janse van Rensburg, M. P. (1991). *Frail aged persons residing in South African homes for the aged who require hospitalisation. Part I. Urban areas*. South African Medical Journal. https://www.ajol.info/index.php/samj/article/view/157934

Wilkinson, M., & Vember, H. (2013). Family members' perceptions and expectations of the use of syringe drivers: A South African study. *International Journal of Palliative Nursing*, *19*(10), 488–494. https://doi.org/10.12968/ijpn.2013.19.10.488

Williams, S. E. I., Carmichael, T. R., Wainstein, T., Hobbs, A., & Ramsay, M. (2015). MYOC mutations in black South African patients with primary open-angle glaucoma: Genetic testing and cascade screening. *Ophthalmic Genetics*, *36*(1), 31–38. https://doi.org/10.3109/13816810.2014.972520

Williams, S. E. I., Whigham, B. T., Liu, Y., Carmichael, T. R., Qin, X., Schmidt, S., Ramsay, M., Hauser, M. A., & Allingham, R. R. (2010). Major LOXL1 risk allele is reversed in exfoliation glaucoma in a black South African population. *Molecular Vision*, *16*, 705–712. http://www.molvis.org/molvis/v16/a80

Wilunda, B., Ng, N., & Stewart Williams, J. (2015). Health and ageing in Nairobi's informal settlements-evidence from the International Network for the Demographic Evaluation of Populations and Their Health (INDEPTH): A cross sectional study Global health. *BMC Public Health*, *15*(1), 1231. https://doi.org/10.1186/s12889-015-2556-x

Wolff, E. M. P. (1978). *Health needs of geriatric patients discharged from hospital*. South African Medical Journal. https://journals.co.za/content/m_samj/54/3/AJA20785135_18933

Wood, L., Robinson, R., Gavine, L., Juritz, J., & Jacobs, P. (2007). A single unit lymphoma experience - Outcome in a Cape Town academic centre. *Transfusion and Apheresis Science*, *37*(1), 93–102. https://doi.org/10.1016/j.transci.2007.06.002

Wyk, C. W., Farman, A. G., & Staz, J. (1977). Oral health status of institutionalized elderly Cape Coloreds from the Cape Peninsula of South Africa. *Community Dentistry and Oral Epidemiology*, *5*(4), 179–184. https://doi.org/10.1111/j.1600-0528.1977.tb01636.x

Wyk, G. W., Farman, A. G., & Staz, J. (1977). Tooth survivial in institutionalized elderly Cape Coloreds from the Cape Peninsula of South Africa. *Community Dentistry and Oral Epidemiology*, *5*(4), 185–189. https://doi.org/10.1111/j.1600-0528.1977.tb01637.x

Xavier Gómez-Olivé, F., Thorogood, M., Clark, B. D., Kahn, K., & Tollman, S. M. (2010). Assessing health and well-being among older people in rural South Africa. *Global Health Action*, *3*(1), 2126. https://doi.org/10.3402/gha.v3i0.2126

Yawson, A. E., Ackuaku-Dogbe, E. M., Seneadza, N. A. H., Mensah, G., Minicuci, N., Naidoo, N., Chatterji, S., Kowal, P., & Biritwum, R. B. (2014). Self-reported cataracts in older adults in Ghana: Sociodemographic and health related factors. *BMC Public Health*, *14*(1), 949. https://doi.org/10.1186/1471-2458-14-949

Yawson, A. E., Baddoo, A., Hagan-Seneadza, N. A., Calys-Tagoe, B., Hewlett, S., Dako-Gyeke, P., Mensah, G., Minicuci, N., Naidoo, N., Chatterji, S., Kowal, P., & Biritwum, R. (2013). Tobacco use in older adults in Ghana: Sociodemographic characteristics, health risks and subjective wellbeing. *BMC Public Health*, *13*(1), 979. https://doi.org/10.1186/1471-2458-13-979

Yorston, D., Gichuhi, S., Wood, M., & Foster, A. (2002). Does prospective monitoring improve cataract surgery outcomes in Africa? *British Journal of*

Ophthalmology, *86*(5), 543–547. https://doi.org/10.1136/bjo.86.5.543

Zengin, A., Fulford, A. J., Sawo, Y., Jarjou, L. M., Schoenmakers, I., Goldberg, G., Prentice, A., & Ward, K. A. (2017). The Gambian Bone and Muscle Ageing Study: Baseline Data from a Prospective Observational African Sub-Saharan Study. *Frontiers in Endocrinology*, *8*(AUG), 31. https://doi.org/10.3389/fendo.2017.00219

Zengin, A., Jarjou, L. M., Prentice, A., Cooper, C., Ebeling, P. R., & Ward, K. A. (2018). The prevalence of sarcopenia and relationships between muscle and bone in ageing West-African Gambian men and women. *Journal of Cachexia, Sarcopenia and Muscle*, *9*(5), 920–928. https://doi.org/10.1002/jcsm.12341

Zimba Kalula, S., Ferreira, M., Swingler, G., Badri, M., & Aihie Sayer, A. (2015). Prevalence of falls in an urban community-dwelling older population of Cape Town, South Africa. *Journal of Nutrition, Health and Aging*, *19*(10), 1024–1031. https://doi.org/10.1007/s12603-015-0664-z

Zimmer, Z., & Dayton, J. (2005). Older adults in sub-Saharan Africa living with children and grandchildren. *Population Studies*, *59*(3), 295–312. https://doi.org/10.1080/00324720500212255

Zwi, A. B., Reid, G., Landau, S. P., Kielkowski, D., Sitas, F., & Becklake, M. R. (1989). Mesothelioma in South Africa, 1976-84: Incidence and case characteristics. *International Journal of Epidemiology*, *18*(2), 320–329. https://doi.org/10.1093/ije/18.2.320
